# Characterizing COVID-19 and Influenza Illnesses in the Real World via Person-Generated Health Data

**DOI:** 10.1016/j.patter.2020.100188

**Published:** 2020-12-13

**Authors:** Allison Shapiro, Nicole Marinsek, Ieuan Clay, Benjamin Bradshaw, Ernesto Ramirez, Jae Min, Andrew Trister, Yuedong Wang, Tim Althoff, Luca Foschini

**Affiliations:** 1 Evidation Health, Inc., San Mateo, CA 94401, USA; 2 Bill and Melinda Gates Foundation, Seattle, WA 98109, USA; 3 Department of Statistics and Applied Probability, University of California Santa Barbara, Santa Barbara, CA 93106, USA; 4 Allen School of Computer Science & Engineering, University of Washington, Seattle, WA 98115, USA

**Keywords:** COVID-19, SARS-CoV-2, influenza-like illness, flu, person-generated health data, digital health, symptom presentation, Fitbit, activity tracker

## Abstract

The fight against COVID-19 is hindered by similarly presenting viral infections that may confound detection and monitoring. We examined person-generated health data (PGHD), consisting of survey and commercial wearable data from individuals' everyday lives, for 230 people who reported a COVID-19 diagnosis between March 30, 2020, and April 27, 2020 (n = 41 with wearable data). Compared with self-reported diagnosed flu cases from the same time frame (n = 426, 85 with wearable data) or pre-pandemic (n = 6,270, 1,265 with wearable data), COVID-19 patients reported a distinct symptom constellation that lasted longer (median of 12 versus 9 and 7 days, respectively) and peaked later after illness onset. Wearable data showed significant changes in daily steps and prevalence of anomalous resting heart rate measurements, of similar magnitudes for both the flu and COVID-19 cohorts. Our findings highlight the need to include flu comparator arms when evaluating PGHD applications aimed to be highly specific for COVID-19.

## Highlights

• We use data from smartphones and wearables from ~7,000 people to compare flu and COVID-19
• While symptoms have some overlap, patients report longer COVID-19 illnesses than flu
• Elevated resting heart rate measures are more frequent around ILI symptoms onset
• It is important to consider flu as a confounder in COVID-19 real-world studies

## The Bigger Picture

In this study, we integrate longitudinal symptoms reports and continuous data from commercial wearables to compare and contrast flu and COVID-19 presentations. We found that, while symptoms constellation between COVID-19 and flu have large overlap, symptoms are significantly more prolonged and severe for COVID-19 than for flu. Similarly, physiological data from commercial wearables showed increased resting heart rate around symptoms onset date that were more severe for COVID-19, but present in milder form for flu as well.

Person-generated health data (PGHD), including data from smartphones and other connected sensors, has the potential to enable applications ranging from individual-level early warnings or population-level hotspot detection for COVID-19. However, for these applications to become a reality, our findings suggest that it is crucial to develop and validate them in the context of other potentially confounding respiratory illnesses, such as the flu.

## Teaser

Person-generated health data (PGHD), including data from smartphones and other connected sensors, has the potential to enable applications ranging from individual-level early warnings or population-level hotspot detection for COVID-19. We show that, when studied outside the clinic, COVID-19 and flu may have overlapping, yet distinct, symptom presentations. For COVID-19 PGHD applications to become a reality, our findings suggest that it is crucial to develop and validate them in the context of other potentially confounding respiratory illnesses, such as the flu.

## Introduction

The emergence of the novel SARS-CoV-2 (COVID-19) pandemic necessitates an understanding of symptom prevalence and progression among individuals with COVID-19, as well as how COVID-19 symptoms compare with those of other infectious diseases. Self-reported data collected at the point of care are being used to help answer key questions around the management of COVID-19 patients,^1^ and real-world data collected via smartphone apps from individuals participating in COVID-19 syndromic surveillance programs^2^, ^3^, ^4^ are being used to perform population-level hotspot detection,^5^ and show promise in understanding symptom presentation outside clinic walls. In addition to self-report, data from commercial sensors may be used for large-scale surveillance of influenza-like illnesses (ILI), given that resting heart rate (RHR)^6^, ^7^, ^8^, ^9^, ^10^ and temperature^11^ change in the presence of an infection. Benefits may come from integrating different digital data sources. For example, a hotspot detection system, including smart thermometers and internet searches, has been shown to provide accurate early-warning indicators of increasing or decreasing state-level US COVID-19.^12^ Syndromic surveillance based on symptom self-report has recently been shown to scale to tens of thousands of responses per day,^5^ and wearables sensors, being worn by one in five Americans,^13^ could further increase the volume of daily feeds of person-generated health data (PGHD) used at the aggregate level for syndromic surveillance and hotspot detection.

In addition to being used in aggregate form for population-level hotspot detection, PGHD is also being proposed as a candidate for individual-level applications, such as to support detection and monitoring of COVID-19 and other respiratory viruses.^14^, ^15^, ^16^, ^17^, ^18^

Along these lines, several efforts are currently underway to explore the potential of using wearable technology to detect COVID-19 onset,^19^, ^20^, ^21^ and preliminary results have shown that wearables may be able to predict COVID-19 symptoms before onset,^22^, ^23^, ^24^ with potential application to large-scale, low-sensitivity/high-frequency testing to enable reopening in the wait for a vaccine.^14^, ^15^, ^16^

However, the lack of a canonical COVID-19 symptom presentation, including how symptoms progress over time,^24^^,^^25^ undermines our ability to track, predict, and control disease progression and manage critical care. In addition, to evaluate performances of any detection system, being that for individual-level early warnings or population-level hotspot detection, it is crucial to compare and contrast symptoms, behavioral, and physiological manifestation with other ILIs, especially flu. Most current COVID-19 research has been developed outside of flu season, but will have to withstand confounding from a surge of flu cases as flu season escalates. This remains true even in spite of the fact that the flu season is expected to be milder due to lockdown measures,^26^ as lockdowns are merely bringing flu prevalence within the same order of magnitude as COVID-19 prevalence.

To address this challenge, we present PGHD contemporaneously shared by individuals who self-reported being diagnosed by a medical provider with either flu or COVID-19. We also present PGHD from a comparator group who were diagnosed with flu before the COVID-19 pandemic. The PGHD consists of self-reported surveys describing symptoms and experiences, and sensor-derived continuous data describing behavior and physiology. The PGHD were collected as part of a large-scale digital participatory surveillance study designed to monitor ILI over the 2019–2020 influenza season. Wearable sensor PGHD (including daily RHR, step counts, and nightly sleep hours), allows us to link continuously measured behavioral and physiological patterns to illness onset.

Our contributions are 2-fold: first, we examine the presentation of COVID-19 symptoms outside of strictly clinical settings both in terms of constellation and time course, and contextualize them with comparisons with seasonal influenza; second, by analyzing wearable data around symptoms onset we show that physiological signals, such as RHR change significantly near symptom onset, as do physical activity measures, such as step counts, although these changes appear to be similar in timing and magnitude across ILI and COVID-19 cohorts. Beyond furthering understanding of mild-to-moderate COVID-19 symptom presentation in the real world as compared with flu, our work suggests that applications leveraging PGHD for COVID-19 detection should be validated not only in cohorts comprised of COVID-19-positive and healthy cases, but also on flu cases, as intermingling the two may significantly increase false positive rates.

## Results

### Data Collection and Cohort Definitions

**Figure 1:**
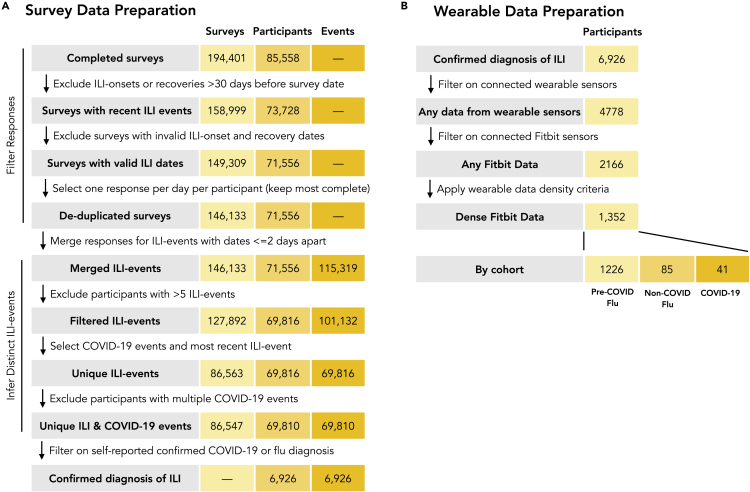
Data Preparation Schema (A) Flow diagram for preparing survey data for analysis. Data preparation consisted of filtering survey responses and merging responses that correspond to the same ILI event. (B) Flow diagram for preparing Fitbit wearable data. Participants with insufficiently dense data were filtered out.

**Figure 2:**
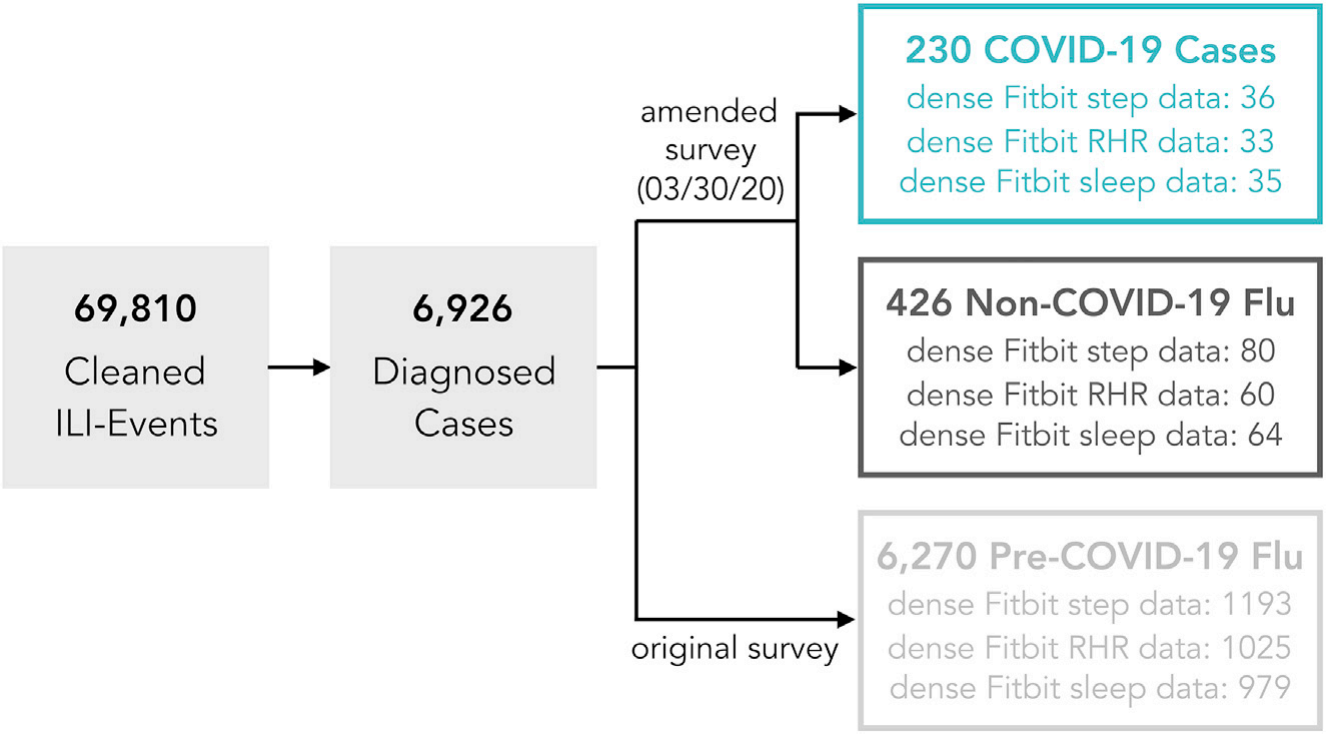
Definition of Analysis Cohorts All participants included in the analyses reported that they sought medical care and were diagnosed with either flu or COVID-19 by a healthcare provider. Participants who indicated they were diagnosed with both flu and COVID-19 (n = 83) were assigned to the non-COVID-19 flu cohort.

We compare a cohort of self-reported diagnosed COVID-19 cases (n = 230) to two groups of diagnosed flu cases: non-COVID-19 flu cases (n = 426), which occurred in the same time frame as the COVID-19 cases, and pre-COVID-19 flu (n = 6,270), which occurred earlier in the 2019–2020 flu season before the outbreak of COVID-19. All cases were identified through curation of a data set of 194,401 responses to a longitudinal survey about ILI symptoms (Figure 1). The rationale of splitting flu comparators into two separate groups is to be able to account for behavioral and physiological confounding factors brought about by lockdown and other measures. In addition, these three primary cohorts were filtered to those participants in each cohort with wearable sensor data and a low fraction of missing data (dense data), focusing on Fitbit wearable sensors (all models). In the COVID-19 cohort, 33 have dense RHR, 35 have dense sleep data, 36 have dense step data, and 41 participants have dense Fitbit data in any of the data channels. In the non-COVID-19 flu cohort, 85 have any dense Fitbit data (60 RHR, 64 sleep, 80 steps), and in the pre-COVID-19 flu cohorts, 1,226 have dense data in any channel (1,025 in RHR, 979 in sleep, 1,193 in steps; Figure 2). The lower counts for RHR stem from the fact that some Fitbit models do not support RHR, while the lower counts for sleep are due to the fact that some participants do not wear the sensor while sleeping. The three primary cohorts, filtered to account for sensor data availability and density are used to estimate changes in wearable data in the neighborhood of self-reported symptoms.

### Survey Results

#### Demographic Differences between COVID-19 and Flu Cases

A demographic summary of the three cohorts is provided in Table 1. A chi-square test of independence was performed for each demographic category to test for significant differences across cohorts. Age group and race differed significantly among the cohorts after applying a Bonferroni correction to adjust for performing five comparisons (age group, p = 0.008; race, p < 0.001), while gender, education, and body mass index (BMI) did not differ significantly. Compared with the pre-COVID-19 flu cohort, the COVID-19 cohort was less likely to be white/caucasian (63.9% versus 70.0%, follow-up two-proportion z test, p = 0.047) and more likely to be Asian or Pacific Islander (9.6% versus 4.6%, p < 0.001). A greater proportion of the non-COVID-19 flu cohort was aged 55 years or older compared with the pre-COVID-19 flu cohort (7.5% versus 3.8%, two-proportion z test, p = 0.001). The demographics of the analyzed cohorts tended to be younger and there were more females compared with those described in the literature for medically attended ILI events for the general US population,^27^ and should be reweighed^28^ before meaningful comparisons can be made with a target population with different demographic characteristics. The cohorts in this study are based on convenience sampling and are not representative of the US population.

**Table 1:** Demographic Summaries for the Full COVID-19 (COVID), Non-COVID-19 Flu (NCF), and Pre-COVID-19 Flu (PCF) Cohorts, as well as the Subset of Each Cohort with Dense Steps, RHR, and/or Sleep Data

	Full Cohorts			Sub-cohorts with Dense Sensor Data		
	COVID (n = 230), %	NCF (n = 426), %	PCF (n = 6,270), %	COVID (n = 41), %	NCF (n = 85), %	PCF (n = 1,226), %
Gender						
Female	70.0	74.4	78.2	80.5	76.5	82.8
Male	28.7	24.6	20.8	17.1	21.2	16.4
Other	0.4	0.7	0.3	0.0	1.2	0.2
Unavailable	0.9	0.2	0.6	2.4	1.2	0.7
Race and ethnicity						
White/caucasian	63.9	66.4	70.0	56.1	75.3	74.8
Hispanic or Latino	7.0	6.8	8.3	9.8	2.4	5.2
Black or African American	3.5	7.0	6.0	7.3	5.9	3.2
Asian or Pacific Islander	9.6	8.0	4.6	9.8	7.1	3.1
American Indian or Alaskan Native	1.3	0.7	0.8	0.0	1.2	0.6
Prefer not to answer	4.8	1.6	1.4	7.3	0.0	1.1
Unavailable	10.0	9.4	8.8	9.8	8.2	12.1
Education						
Did not finish high school	2.2	1.4	1.8	0.0	0.0	1.1
High school diploma or GED	8.7	11.5	12.2	7.3	15.3	9.9
Some college, no degree	21.3	24.2	23.0	19.5	20.0	22.0
Trade/technical/vocational training	4.3	5.4	5.3	7.3	7.1	5.6
College degree	34.8	30.3	36.9	29.3	31.8	39.0
Graduate degree	17.4	18.3	14.3	22.0	20.0	15.8
Doctorate degree or MD	3.0	3.1	1.5	2.4	0.0	1.6
Prefer not to answer	0.0	0.2	0.1	0.0	1.2	0.1
Unavailable	8.3	5.6	4.7	12.2	4.7	4.9
Age (years)						
<25	20.9	16.4	17.9	14.6	11.8	9.2
25–34	40.0	37.3	39.7	41.5	37.6	38.4
35–44	22.6	23.9	25.8	26.8	21.2	28.2
45–54	10.4	14.8	12.2	9.8	16.5	17.0
55+	5.2	7.5	3.8	4.9	12.9	6.7
Unavailable	0.9	0.0	0.5	2.4	0.0	0.4
BMI						
<18.5	3.5	4.2	2.6	2.4	4.7	2.4
18.5–24.9	27.0	21.8	24.0	22.0	21.2	21.9
25.0–29.9	24.3	22.3	24.3	22.0	28.2	24.6
30+	32.2	37.6	38.4	43.9	34.1	42.1
Unavailable	13.0	14.1	10.8	9.8	11.8	9.0

Note that table percentages may not add up to exactly 100% due to rounding applied during formatting.

#### Healthcare Interactions Differ between COVID-19 and Flu Cases

Although all patients had to report seeking medical care and being diagnosed by a medical provider with either flu or COVID-19 to be included the analyses, locations of medical care, hospitalization rates, and medication prescription rates differed significantly across the three cohorts (chi-square tests of independence with a Bonferroni correction, all p < 0.001; summarized in Table 2). Compared with non-COVID-19 flu and pre-COVID-19 flu patients, COVID-19 patients were less likely to seek care at a primary care clinic (37.4% versus 50.2% for non-COVID-19 flu, p = 0.002, versus 45.7% for pre-COVID-19 flu, p < 0.001) or urgent care facility (16.1% versus 23.5% for non-COVID-19 flu, p = 0.026, versus 39.1% for pre-COVID-19 flu, p < 0.001) and more likely to seek care in an emergency room (17.0% versus 8.2% for non-COVID-19 flu, p < 0.001, versus 6.9% for pre-COVID-19 flu, p < 0.001) or other location (37.4% versus 50.2% for non-COVID-19 flu, p = 0.002, versus 45.7% for pre-COVID-19 flu, p < 0.001). Informal review of the text responses provided for "other" locations indicated that COVID-19 patients were more likely to seek care via telehealth services.

**Table 2:** Summaries of Medical Care-Seeking Behaviors and Outcomes for the COVID-19 (COVID), Non-COVID-19 Flu (NCF), and Pre-COVID-19 Flu (PCF) Groups

	COVID (n = 230), %	NCF(n = 426), %	PCF (n = 6,270), %
Medical care location			
Primary care clinic	37.4	50.2	45.7
Urgent care facility	16.1	23.5	39.1
Emergency room	17.0	8.2	6.9
Ear, nose, and throat clinic	2.2	2.1	0.8
Infectious disease clinic	1.7	1.2	0.4
Other	10.9	4.7	4.3
Multiple locations	14.8	10.1	2.8
Hospitalized			
Yes	36.1	15.7	7.1
No	63.9	83.6	92.6
Unavailable	0.0	0.7	0.3
Prescribed medication			
Yes	62.2	67.1	79.4
No	37.0	30.8	19.2
Do not know/remember	0.9	1.4	1.1
Unavailable	0.0	0.7	0.3

Overall statistics are shown, including where medical care was sought, whether patients were hospitalized, and whether they were prescribed medication.

COVID-19 patients were more likely to be hospitalized (36.1%) than non-COVID-19 flu (15.7%, p < 0.001) and pre-COVID-19 flu (7.1%, p < 0.001) patients. Interestingly, a greater proportion of patients with recent flu events were hospitalized than those with flu events earlier in the season (15.7% non-COVID-19 flu versus 7.1% pre-COVID-19 flu, p < 0.001). This result may relate to the fact that a higher proportion of individuals in the non-COVID-19 cohort were older than 55 years. In addition, provider behavior during the initial stages of the COVID-19 pandemic when rapid diagnostic testing was not widely available may have prompted medical providers to admit patients seeking care with ILI symptoms to hospitals at a higher rate than would have otherwise been observed.

The COVID-19 and non-COVID-19 flu cohorts were less likely to be prescribed medication (baloxavir marboxil, oseltamivir, zanamivir, antibiotics, and/or other) than the pre-COVID-19 flu cohort (p < 0.001 and p < 0.001), but the medication rates between COVID-19 and non-COVID-19 flu patients did not differ significantly (p = 0.202).

#### Differing Presentation of COVID-19 and Flu Symptoms

A summary of self-reported symptom prevalences for the COVID-19 and flu cohorts is reported in Table 3. The most common symptoms across all groups included cough, headache, body muscle ache, fatigue, and fever. Symptoms prevalence was significantly different across the three cohorts (chi-square test of independence, p < 0.001). All follow-up pairwise symptom comparisons were tested with two-proportion z tests and a Bonferroni correction was applied for performing 33 tests.

**Table 3:** Summary of Self-reported Symptoms for the COVID-19 (COVID), Non-COVID-19 Flu (NCF), and Pre-COVID-19 Flu (PCF) Cohorts

	Symptom Prevalence			Peak Symptom Day Relative to Illness Onset		
	COVID (n = 230), %	NCF (n = 426), %	PCF (n = 6,270), %	COVID	NCF	PCF
Cough	84.3	71.6	85.1	5	3	3
Headache	71.3	68.1	74.3	4	3	3
Body muscle ache	66.1	67.1	80.8	4	2	2
Shortness of breath	65.7	24.2	N/A	6	8	N/A
Fatigue	61.7	54.7	70.9	5	3	3
Fever	61.3	62.0	74.6	5	2	2
Chills or shivering	53.5	55.4	69.3	4	3	2
Sore throat	51.7	48.8	61.1	3	2	3
Nasal congestion	49.6	49.3	65.4	5	2	3
Chest pain/pressure	49.6	19.7	N/A	6	2	N/A
Sweats	42.2	47.4	56.3	5	2	2
Anosmia	38.3	15.5	N/A	7	3	N/A
Sneezing	37.0	36.9	48.2	4	2	3

Symptom prevalence refers to the percentage of the cohort reporting the symptom at any time during the ILI event and symptoms are sorted by most (top) to least (bottom) prevalence in the COVID-19 cohort. The day of peak symptom occurrence relative to illness onset corresponds to the maximum of a centered 5-day rolling mean of day-by-day symptom prevalence for each cohort. Some symptoms (i.e., shortness of breath, chest pain/pressure, and anosmia) were only included in the updated survey and therefore are not available for the pre-COVID-19 flu cohort. N/A, not applicable.

Compared with the non-COVID-19 flu cohort, patients with COVID-19 were significantly more likely to report experiencing cough (84.3% versus 71.6%, p < 0.001), loss of sense of smell (anosmia; 38.3% versus 15.5%, p < 0.001), persistent pain or pressure in the chest (49.6% versus 19.7%, p < 0.001), and shortness of breath or difficulty breathing (65.7% versus 24.2%, p < 0.001). These are generally accepted as the canonical symptoms of COVID-19.^29^ Although it is important to note that, with the exception of cough, while these symptoms have moderate positive predictive value (higher relative prevalence in COVID-19 cases as compared with flu), they are still relatively insensitive markers of COVID-19 (low absolute prevalence in COVID-19 cases).

Compared with the pre-COVID-19 flu cohort, the COVID-19 cohort was significantly less likely to report experiencing body muscle ache, fever or feeling feverish, nasal congestion or runny nose, sneezing, chills or shivering, and sweats (all p < 0.001). Several symptoms (i.e., shortness of breath, anosmia, and chest pain) could not be compared between the COVID-19 and pre-COVID-19 flu cohorts because they were not included in the original survey.

With the exception of headache, all symptoms were significantly less prevalent in the non-COVID-19 flu cohort relative to the pre-COVID-19 flu cohort. One possible reason for the difference in symptom presentations in the two flu cohorts is that the 2019–2020 flu season consisted of two waves of different flu strains: strain B (Victoria lineage) appeared earlier on and was followed by strain A (H1N1-pdm09).^30^ Vaccines for the 2019–2020 season were well-matched against circulating strain A but not as well-matched against strain B,^31^ which could account for more mild symptom presentation in the recent flu cases in the non-COVID-19 flu cohort compared with cases in the pre-COVID-19 flu cohort.

**Figure 3:**
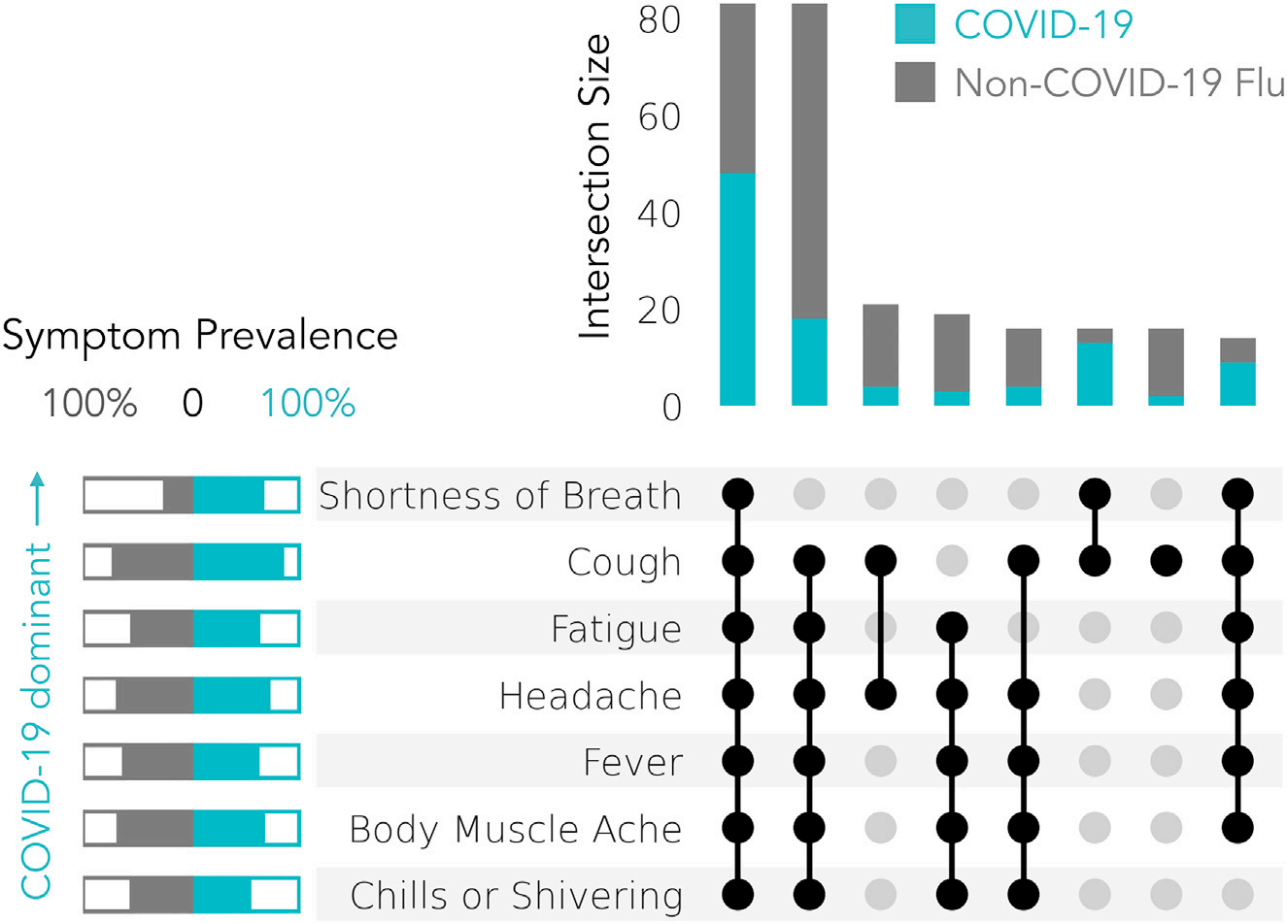
Co-occurrence of Self-Reported Symptoms in COVID-19 Cases or Non-COVID-19 Flu Cases Only the top 5 most prevalent symptoms in each cohort are included in the symptom sets and only symptom sets that represent 2% or more of total COVID-19 (n = 230, blue) and non-COVID-19 flu cases (n = 426, gray) are plotted. Symptoms are sorted by their relative prevalence in COVID-19 (top) versus non-COVID-19 flu (bottom) cases.

We also examined the prevalence of co-occurring sets of symptoms for the COVID-19 and non-COVID-19 flu cohorts (Figure 3). The pre-COVID-19 flu cohort was excluded from this analysis for two reasons. First, comparing the COVID-19 and non-COVID-19 flu cohorts provides two cohorts that are comparable contemporaneously. Second, due to the fact that the pre-COVID-19 flu cohort utilized a survey requesting only a subset of symptoms, adding this cohort was of limited utility in assessing symptom constellations. For simplicity of illustration, we unioned only the five most prevalent symptom sets in each cohort, which resulted in a subset of seven individual symptoms: cough, headache, fever, fatigue, body muscle ache, chills or shivering, and shortness of breath. The two most common symptom sets consisted of all symptoms, which was predominated by COVID-19 cases, and all symptoms except for shortness of breath, which was predominated by non-COVID-19 flu cases. The symptom pair of shortness of breath and cough, and the set of all symptoms other than chills or shivering were also more indicative of COVID-19 than non-COVID-19 flu.

#### COVID-19 Symptoms Tend to Peak Later and Last Longer than Flu

**Figure 4:**
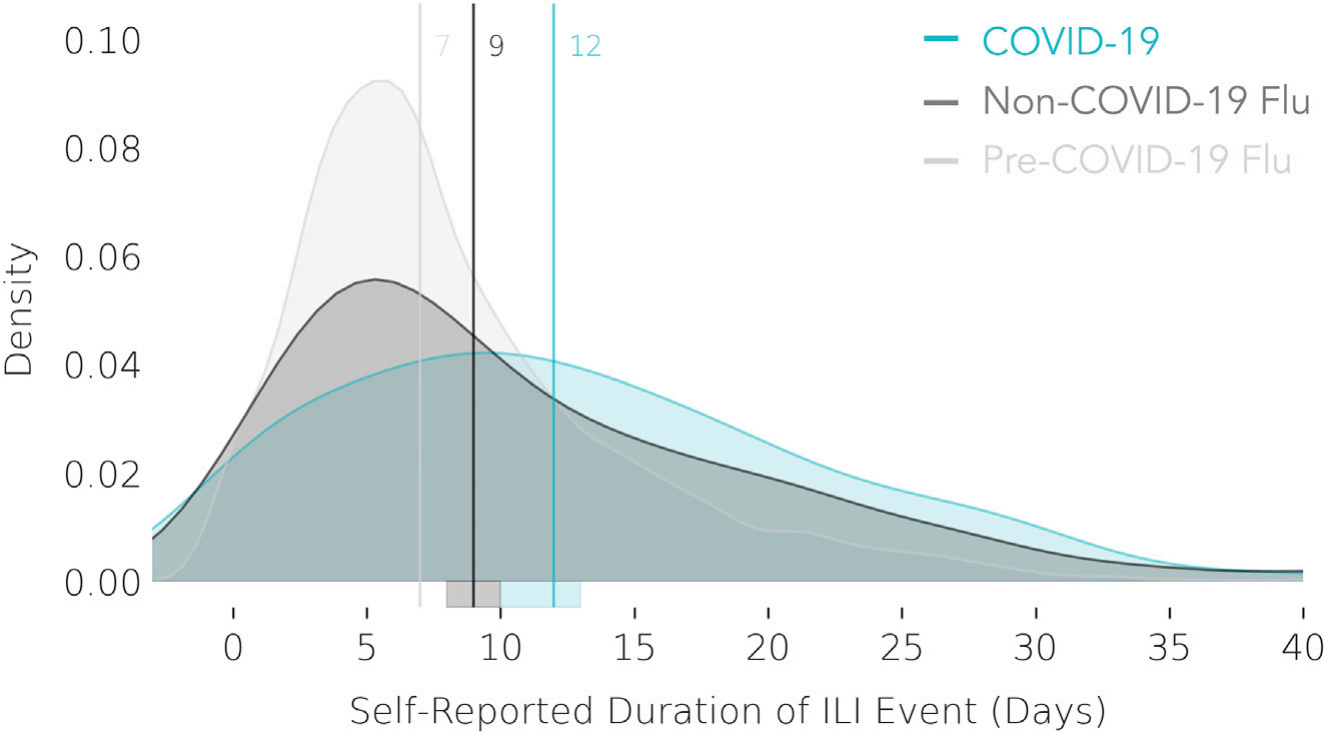
Self-Reported Illness Duration in Days for COVID-19, Non-COVID-19 Flu, and Pre-COVID-19 Flu Cases Vertical lines denote the median illness duration. COVID-19 (n = 230, blue); non-COVID-19 flu (n = 426, gray); pre-COVID-19 flu (n = 6,270, light gray). Error bands around foot of vertical median lines represent bootstrapped 95% confidence intervals.

The duration of each ILI event (illness period) in days was calculated from self-reported dates of illness onset and illness recovery (Figure 4). COVID-19 illnesses tended to last longer than flu illnesses, lasting a median of 12 days, compared with a median of 9 days for non-COVID-19 flu illnesses and 7 days for the pre-COVID-19 flu illnesses. Compared with the non-COVID-19 and pre-COVID-19 flu cohorts, most of the COVID-19 cohort experienced a longer duration of illness (Mood’s median test, p = 0.003 and p < 0.001, respectively). The observed slightly longer duration of non-COVID-19 flu as compared with pre-COVID-19 flu, despite the overall milder symptoms of non-COVID-19 flu, may depend on potential contamination of the non-COVID-19 flu with COVID-19 cases, which have a longer duration on average, with symptoms mild enough and non-specific enough to not warrant a test, and thus get reported as flu.

**Figure 5:**
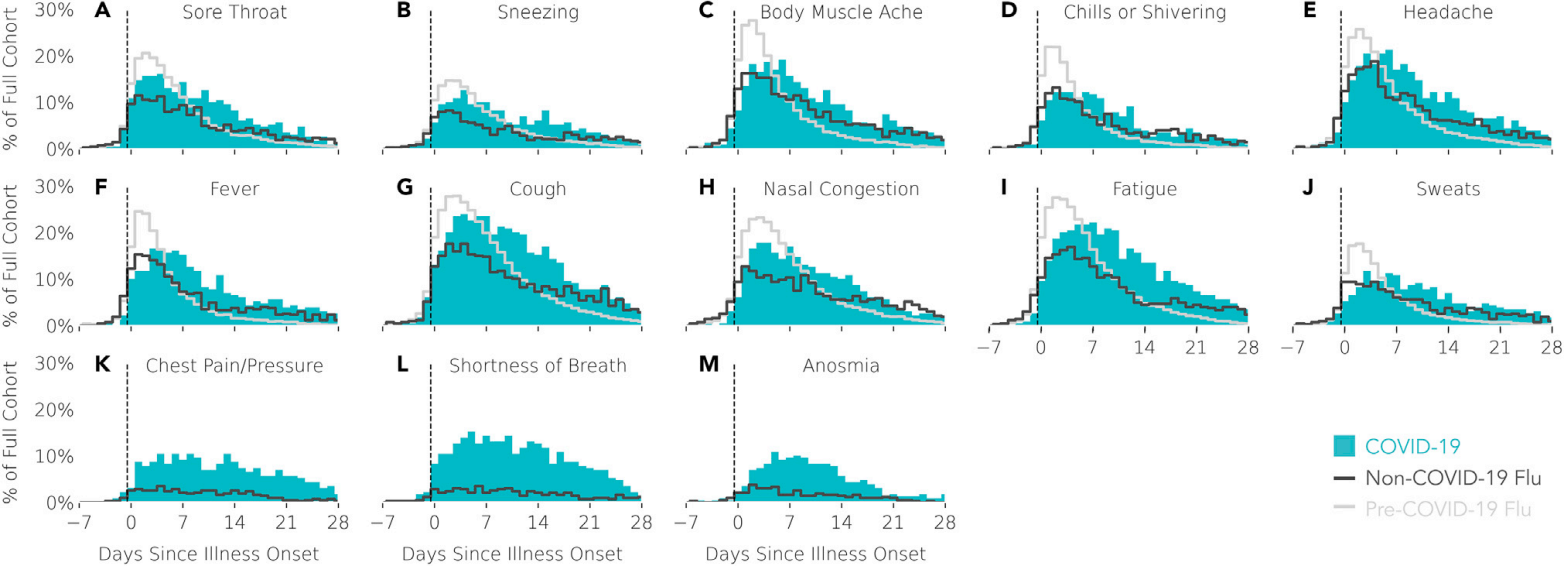
Self-Reported Symptom Prevalence over Time Relative to Illness Onset (Day 0; Also Self-Reported) For a Figure360 author presentation of this figure, see https://doi.org/10.1016/j.patter.2020.100188. Prevalence is reported as a percentage of the full cohort of COVID-19 cases (n = 230; blue), non-COVID-19 flu cases (n = 426; dark gray), or pre-COVID-19 flu cases (n = 6,270; light gray trace). (A–M) Each subplot contains data for one symptom and symptoms are sorted by peak symptom occurrence (earliest to latest) for the COVID-19 cases. Note that negative values of “Days since illness onset” reflect the number of days preceding the self-reported illness onset.

Symptom prevalence across each cohort for each day after the date of self-reported illness onset is illustrated in Figure 5, and the days of peak symptom occurrence for each cohort are reported in Table 3. The peak days of symptom occurrence were significantly different across the three cohorts for all symptoms (Mood’s median test: p < 0.001). The peak days were significantly different for all symptoms when comparing non-COVID-19 flu and pre-COVID-19 flu cohorts as well as when comparing COVID-19 and pre-COVID-19 flu cohorts. Compared with the non-COVID flu cohort, the COVID-19 cohort had significantly different peak symptom days for fever (p < 0.001), cough (p = 0.014), nasal congestion (p < 0.001), fatigue (p < 0.001), sweats (p = 0.011), chest pain (p = 0.007), shortness of breath (p = 0.006), and anosmia (p = 0.007), but not for other symptoms. In general, day-by-day symptom prevalence peaked later for the COVID-19 cases compared with the two groups of flu cases. With the exception of shortness of breath for the non-COVID-19 flu cohort, all symptoms peaked 2–3 days after illness onset in both flu cohorts. In contrast, COVID-19 symptoms peaked 3–7 days after illness onset, with most symptoms peaking 4–5 days after illness onset. Some of the latest peaking symptoms are those that are most tightly associated with COVID-19, including fever, cough, nasal congestion, fatigue, shortness of breath, chest pain or pressure, and anosmia (Figures 5F–5M, respectively).^29^

### Wearable Data Results

#### Demographics of Participants with Dense Wearable Sensor PGHD

Data from commercial Fitbit sensors were available for at least 1 day between 2019-11-01 and 2020-05-13 for approximately 31% of all participants. A smaller subset of these participants met the criteria for sensor data density (see Experimental Procedures) and were included in the analysis, including 41 (18%) COVID-19 patients, 85 (20%) non-COVID-19 flu patients, and 1,226 (20%) pre-COVID-19 flu patients. The demographics of the cohorts with dense sensor data are described in Table 1. We tested for demographic differences among participants with and without dense sensor data using the same two-step statistical testing procedure described previously. Pooling across the three cohorts, compared with participants without dense Fitbit data (n = 5,574), those with dense Fitbit data (n = 1,352) were more likely to be female (p < 0.001), white (p < 0.001), obese (30+ BMI, p = 0.003), and in an older age group (45–54, p < 0.001; 55+, p < 0.001).

#### COVID-19 Illness Onset Was Associated with Elevated RHR

**Figure 6:**
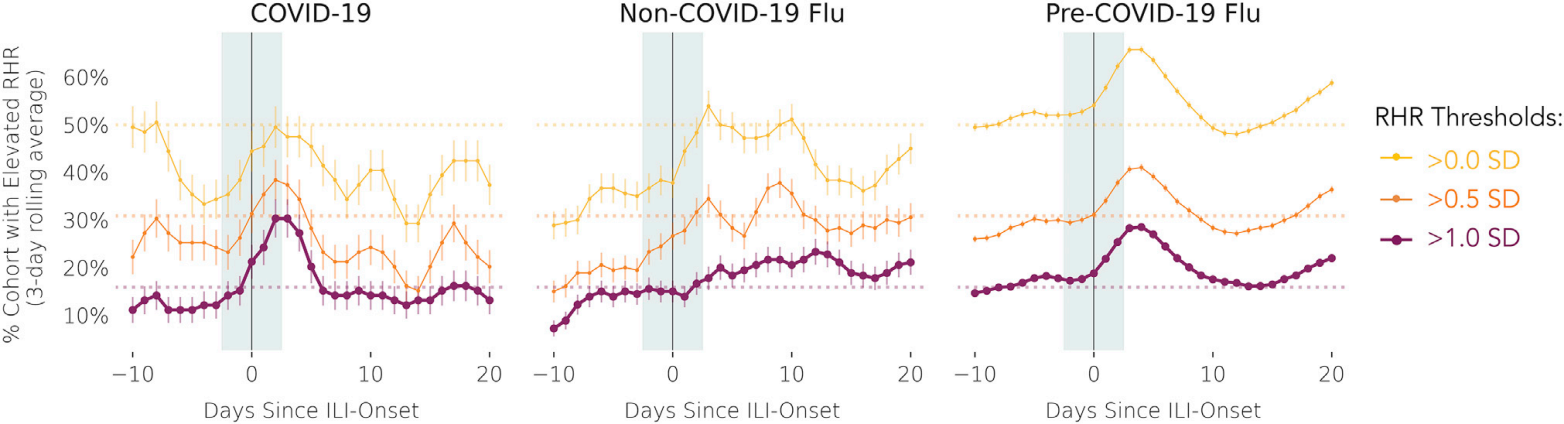
Fraction of Participants with Elevated Daily RHR on Days Surrounding Illness Onset (Day 0) Elevated RHR is defined as being greater than 1, 0.5, or 0 standard deviations (SD) over all daily RHRs observed during the combined baseline and symptomatic periods. Cohorts refer to the sub-cohorts with dense RHR data: COVID-19 (n = 33; left), non-COVID-19 flu (n = 60; middle), and pre-COVID-19 flu (n = 1,025; right). Error bars represent the SD of the sample proportions.

Given the known association between elevated RHR and the inflammatory immune system response,^9^^,^^10^ we examined the prevalence of elevated RHR around ILI events. RHR is computed by commercial Fitbit sensors. While the exact algorithm estimating RHR is proprietary to Fitbit,^32^ it approximately coincides with heart rate observed during periods of deep sleep or inactivity. Following a previously described methodology,^8^ we define an RHR measurement as elevated for a participant if they are 0 (minimally elevated), 0.5 (moderately elevated), or 1 (highly elevated) standard deviation(s) above mean RHR, where both mean and standard deviation are computed for each participant over the entire 189-day-long observation window, consisting of both the baseline and ILI event windows. Figure 6 illustrates the fraction of each cohort with elevated RHR, for different elevation thresholds, for each day relative to symptom onset for each of the three cohorts. In both the COVID-19 and pre-COVID-19 flu cohorts, the prevalence of highly elevated RHR was highest in the first days after illness onset. In particular, the percent of COVID-19 patients with highly elevated RHR was higher around the onset of COVID-19 from days −2 to 2 (25%), when infectivity is at its peak and isolation interventions could have maximum effectiveness,^16^ as compared with days −10 to −5 (13%, two-proportion z test p = 0.005) when transmissibility is less likely.^16^ Remarkably, the percentage of the COVID-19 cohort with highly elevated RHR around illness onset was greater than that of the non-COVID-19 cohort (16%, p = 0.026), but did not differ from that of the pre-COVID-19 cohort (22%, p = 0.454), which suggests that RHR elevation alone may not be a specific marker of infection. Non-COVID-19 flu appears to display a noticeable peak only for the moderately elevated RHR, which corroborates the hypothesis of milder symptom severity of non-COVID-19 flu as compared with pre-COVID-19 flu.

#### Activity Decrease during COVID-19 and Flu Illnesses

**Figure 7:**
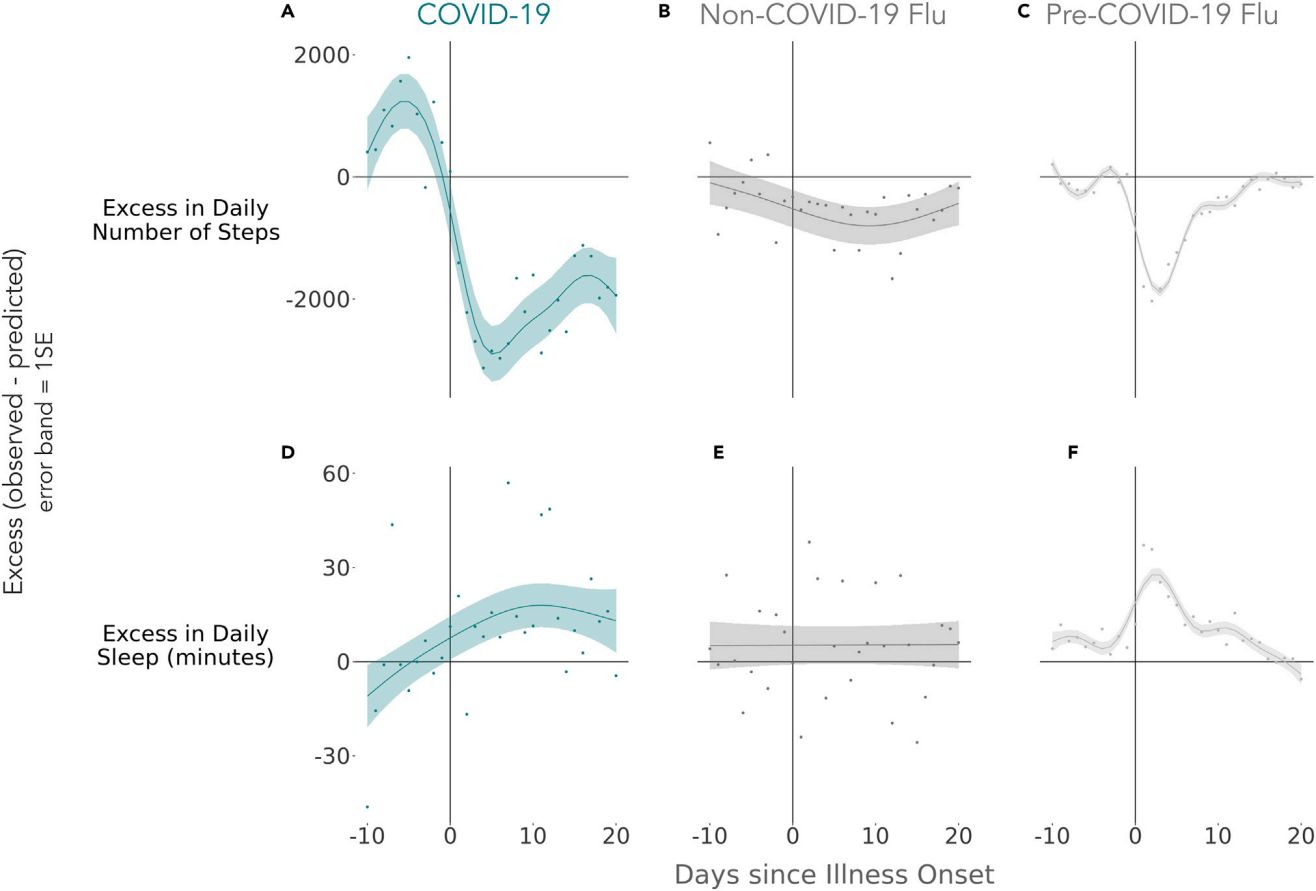
Deviations from Typical Healthy Behavior and Physiology Observed during ILI Events Three measurement channels were studied: daily number of steps, daily mean RHR, and daily sleep minutes. Deviation from the norm was quantified as difference (excess) between observed values and estimates from a model fit only to symptom-free days (i.e., days outside the window of −10 through +20 days surrounding ILI onset). Greater excess indicates greater deviations from typical behavior. Sample sizes across cohorts and channels: steps analysis: (A) COVID-19, n = 36; (B) non-COVID-19, n = 80; (C) pre-COVID-19, n = 1,193; sleep analysis: (D) COVID-19, n = 35; (E) non-COVID-19, n = 64; (F) pre-COVID-19, n = 979. Error bands represent 1 SE.

To quantify the impact of COVID-19 and flu on objective, sensor-based measures of behavior, we examined the extent to which daily steps and sleep deviated from expected measurements before and after illness onset. To this end, we first estimated for each participant what their expected measurement (total sleep duration, step counts) for a given day would be during illness *had they not been sick*, based on models fit on a pre- and post-illness baseline period (see Experimental Procedures for details). Subsequently, we computed excess activity during the ILI event as the difference between the unobserved estimated "healthy day" measurement and the observed one during a sick day. Generalized additive models were fit to the excess values separately for each cohort (mgcv package for R) and the resulting regression splines were used for visualizations. Figure 7 illustrates the impact of illness in terms of excess daily steps lost and additional minutes of sleep. Reductions in daily step counts were more marked for pre-COVID-19 flu than they were for non-COVID-19 flu, supporting the hypothesis of milder symptoms of non-COVID-19 flu, and the fact that mobility-reducing shelter-in-place measures during non-COVID-19 flu limit the maximum amount of lost step counts. Reduction in daily steps were also more marked and prolonged for COVID-19 patients as compared with non-COVID-19 flu and pre-COVID-19 flu patients. This may be explained by the adoption of more stringent self-imposed quarantine measures after a COVID-19 diagnosis (89% of the COVID-19 cohort reported being told by a medical provider to self-quarantine compared with only 57% of the non-COVID-19 flu cohort), but given the fact that excess is computed taking into consideration reduced mobility caused by shelter-in-place measures, the observed reduction could also be a reflection of the more prolonged illness durations in the COVID-19 patients as captured by self-reported symptoms and discussed in previous sections. In addition, the step reduction appears to persist beyond the 2-week default quarantine mandate period, and if confirmed on longer time horizons it could be an indication of COVID-19 patients experiencing "long COVID"^33^: a phenomenon in which symptoms persist for many weeks or months after the illness is meant to subside.

Sleep changes are largely inconclusive, as the post-onset total sleep time increase observed for pre-COVID-19 flu may be explained by changes in sleeping schedules during sick days that would be less prominent for the COVID-19 and non-COVID-19 flu cases, which are concurrent with widespread shelter-in-place measures. When directly comparing the non-COVID-19 flu and COVID-19 cohorts, post-onset sleep excess seems to persist for prolonged periods, hinting once again to a longer duration of symptoms and disruption of behavior brought about by a COVID-19 infection.

## Discussion

We present a report on PGHD, including longitudinal symptoms reports and linked physiologic and behavior data from commercial wearables collected remotely in real-life settings for 6,696 diagnosed flu and 230 diagnosed COVID-19 patients.

Chest pain, shortness of breath, and anosmia, as well as combinations of these symptoms (e.g., shortness of breath and coughing) were more prevalent in COVID-19 as compared with non-COVID-19 flu. Other symptoms, including fatigue and cough, were more prevalent later after illness onset for COVID-19 cases relative to flu cases. Similarly, patients reported longer illness duration for COVID-19 (median of 12 days) as compared with non-COVID-19 and pre-COVID-19 flu illnesses (9 and 7 days, respectively).

Differences in self-reported symptoms are supported by data from wearable sensors. We observed larger, more prolonged reductions in daily step counts for COVID-19 patients as compared with other groups. This is consistent with the observed longer illness durations for COVID-19 generally, sometimes lasting weeks or months in what is now being referred to as "long COVID.”^33^ Wearable sensors could be particularly useful in monitoring recovery from long COVID, as their unobtrusive nature may guarantee steady data collection for longer time horizons for which daily self-reporting of symptoms may become too burdensome for the participant.^34^

We observed a significantly increased fraction of participants with elevated RHR measurements in the 2 days surrounding ILI symptom onset. This has previously been observed for other ILIs^7^ and is now also observed for COVID-19 patients. Several recent works have explored use of wearable data, including RHR, to detect symptoms of COVID before they appear toward applications that can prompt users to intervene in pre-symptomatic disease phases and curb the spread of infection (e.g., self-quarantine while waiting for a confirmatory test).^22^, ^23^, ^24^^,^^35^ While these systems have shown moderate discriminative ability between COVID-19 patients versus healthy persons in retrospective cohorts, our findings suggest that the specificity of those systems should also be measured as compared with flu patients, as they will be the overwhelming majority as flu season starts. If specificity versus flu and other respiratory viruses cannot be demonstrated, early-warning systems triggered on wearable data should be considered as more non-specific “infection screening,” and therefore be coupled with appropriate confirmatory testing mechanisms that can help to quickly relieve self-imposed quarantine of non-COVID-19 infections.^17^^,^^18^

From a methodological perspective, we note that we found it helpful to be able to compare two different flu comparator arms, one of which was contemporaneous with COVID-19, as it allows partial control for the society-scale shifts in behavior that the pandemic has brought about. For example, we noted that medication prescription rates were lower for the COVID-19 and non-COVID flu cohorts as compared with the pre-COVID flu cohorts. This may suggest that, during the pandemic, there may be additional barriers to treatment that have arisen due to lockdown measures and changes in attitudes toward risks in seeking care. Without two flu comparator arms, including one that is pre-pandemic, it would have been impossible to disentangle reduction of prescriptions coming from lockdown versus reduction coming from fewer available treatments for the novel COVID-19. While we do not have additional data to investigate these hypotheses, they may be worthwhile directions for further research.

### Biases and Limitations

The studied cohorts come from convenience samples that are not representative of the US population at large. In particular, we note that African Americans and males, alongside older individuals, are underrepresented in our cohort, thus limiting the generalizability of our findings. Increasing access and usage of these tools in these risk groups is of critical importance.^36^

Differences in the rate of the COVID-19 occurrence across demographic groups and disease severity levels have received attention recently,^37^, ^38^, ^39^, ^40^, ^41^, ^42^ with increasing evidence that some racial and ethnic minority groups are being disproportionately affected by COVID-19, and preliminary findings point to possible differences with other ILIs as well.^43^, ^44^, ^45^ Large-scale connected populations could be a key tool in examining the impact COVID-19 is having across demographic and geographic groups, helping to highlight vulnerable populations and target care delivery.^4^ However, the cohort of individuals utilizing wearable data in this study may not reflect the heterogeneity of the general populace and, as such, results relying on wearable data may not generalize to all new populations.

Hospitalization rates for the COVID-19 and non-COVID-19 flu participants in this study were higher than national estimates from the same time frame,^46^, ^47^, ^48^ which could be due to the fact that participants were required to report that they both sought medical attention *at a clinic or urgent care facility*, and were diagnosed by a medical provider to be included in the analysis. Due to shortages in testing availability and stringent testing criteria at the early stage of the pandemic and at the time of these surveys, our cohorts of diagnosed individuals may be composed of people who had more severe symptoms and thus were more likely to seek medical care, those with greater access to healthcare resources, or those who were already hospitalized when tested. Higher hospitalization rates in the post-pandemic cohorts may also be attributed to elevated monitoring and quarantining efforts in an attempt to control spreading. In addition, given that only individuals who sought medical care were included in the analysis, this may skew the symptom presentation toward more severe symptoms, such as shortness of breath or difficulty breathing, as opposed to less worrisome symptoms (e.g., sneezing). In parallel, the medication prescription rate is likely to vary by patient symptom presentation and severity upon healthcare visit and were not explored further by medication type or adherence in this study.

Another limitation comes from the fact that the analysis considers self-reported symptoms and self-reported diagnostic test confirmation. During the early period of the pandemic, diagnostic tests for COVID-19 suffered from highly heterogeneous administration policies and inaccuracies, which may have biased cohort composition. Finally, the surveys used to capture symptoms between the earlier pre-COVID flu cohort and the COVID-19 and non-COVID flu cohorts used slightly different symptom sets, making direct comparison of symptom prevalence infeasible in some cases.

### Outlook

Using PGHD from self-reported symptoms, in combination with physiological and behavioral measures continuously and unobtrusively tracked by commercial wearable sensors, allows us to confirm and contextualize learnings that may be otherwise lost when considering each data stream in isolation. As more PGHD is collected and evidence is created, it is important to keep in mind that inclusion of additional data streams may increase utility, but does not immediately yield increased representativeness, especially when new streams continue to be digitally mediated and are thus biased toward access to digital technologies.^49^ Making sure that PGHD generation is not restricted to a niche of the population is perhaps the current biggest limitation, and the most important agenda item on which we need to continue to make progress.

In the specific context of COVID-19, our findings support the case made by recent work that data from wearable sensors may provide low-sensitivity testing capability with daily frequency.^22^, ^23^, ^24^^,^^35^ Low-sensitivity/high-frequency testing when combined with a low-delay confirmatory testing strategy has been shown by computation models to significantly reduce prevalence of spreading with minimal burden on pre-emptive quarantine for false positives.^14^^,^^16^ Therefore, wearables could potentially support use cases, such as return to work and college reopening,^18^ where most of the cohort can be asked to wear the sensors frequently.^17^ To better understand feasibility, however, further research is needed. First, it is important to accurately quantify the sensitivity of wearable-based alert systems in prospective validation, and especially for asymptomatic/pre-symptomatic patients (who collectively seem to be responsible for more than 40% of the total infections).^50^ To this end, an understanding of the ability of PGHD to detect pre-symptomatic and asymptomatic spreading cannot be derived from data based solely on symptoms. Therefore, studies designed to combine PGHD with direct measures of infectivity (e.g., PCR tests) constitute a necessary next step to understand sensitivity to asymptomatic/pre-symptomatic infections, and they are currently under development.^51^

Second, as highlighted by the current work, it is important to understand the specificity of any PGHD-based early-warning system as compared with other respiratory diseases, such as the seasonal flu, that may have similar physiological and behavioral fingerprint in addition to a large symptom overlap. We encourage researchers presenting results of COVID-19 early-warning systems based on PGHD in real-world settings to contextualize their findings, taking the confounding effect of flu into account, instead of assuming "non-COVID-19" to be a synonym of healthy controls. Despite the expectation of reduced impact due to lockdown measures, flu is still seen as a confounding factor for population-level estimates of the burden of respiratory illness when COVID-19 and flu coexist.^52^ Analogously, flu must be taken into serious consideration as a confounding factor for any PGHD-based applications, being that at population or individual level. An accurate understanding of detection parameters (sensitivity, specificity, lag) in true real-world settings is crucial not only to understand feasibility, but also to understand how to shape the interaction between the system and its users. For example, we must consider how to incorporate confirmatory tests that test on multiple pathogens, not only COVID-19. The usability, including perceptions around effectiveness, burden, and privacy, will ultimately define adoption of such systems at scale.^49^^,^^53^

As care becomes more decentralized and telehealth becomes more widespread,^54^ PGHD can become a valuable tool on an individual level as patients transition in and out of care.^55^ In addition to providing support to individual-level early-warning systems and population-level hotspot detections, PGHD could enable monitoring of recovery from symptoms, as the unobtrusive nature of sensor-based PGHD makes consistent monitoring possible over the weeks and months of long-COVID recovery.

The vast majority of learning about COVID-19 has come from real-world data sources, such as health records and claims.^56^ PGHD can be a crucial addition, adding a large-scale understanding of early signals, several days before impact is seen at centers of care. As the COVID-19 pandemic continues to develop, and as future annual ILI waves arrive, understanding and correctly reacting to symptom presentation will be critically important. These results support not only an emerging picture that COVID-19 has a distinct presentation, but highlight the power of PGHD, digital health, and connected populations in broadly and remotely monitoring health status.

## Experimental Procedures

### Resource Availability

#### Lead Contact

Luca Foschini, luca@evidation.com.

#### Materials Availability

The amended questionnaire is available in Note S2. The original questionnaire consisted of a subset of questions from the amended questionnaire; the original questionnaire included all questions except for questions 1, 8–9, 14–16, 18, 28–30, 32, 36–38.

#### Data and Code Availability

The completed coded curated study data can be requested by qualified researchers via the Sage Synapse platform^57^ here: https://www.synapse.org/#!Synapse:syn22891469/.

Code for reproducing all analyses in the manuscript can be found here:

https://github.com/evidation-datascience/COVID19_baseline_paper.

### Data Collection

Achievement is a mobile consumer application that rewards and enables members to participate in research by completing questionnaires and sharing data from commercial-grade wearable sensors.^58^, ^59^, ^60^ Since 2017, Achievement has been used to run a participatory ILI surveillance program, examining annual waves of influenza virus infections.^7^ The 2019–2020 version of the program recruits individuals who have experienced ILI symptoms in the past 7 days to collect information on the date of illness onset and/or recovery, detailed symptoms, healthcare interactions and outcomes, medications, and household characteristics. The questionnaire was designed with inspiration from Flu Near You,^6^ as well as input from public health and clinical infectious disease experts. On 2020-03-30, the questionnaire was updated to include questions that specifically address COVID-19, including questions about COVID-19 diagnosis, testing, and social distancing measures, and an expanded list of symptoms, including shortness of breath, chest pain, and anosmia. The contents of the original and updated questionnaires are included in Note S2.

Participants agreed to share survey responses and activity data from connected wearable sensors. Responses to the original and updated surveys, collected between 2019-12-02 and 2020-04-27, comprised the initial survey dataset and included a total of 194,401 responses from 85,558 unique participants. The sensor data analyzed in this project consisted of minute-by-minute step counts, RHR recordings, and sleep states from 2019-11-01 to 2020-05-13 for the subset of participants with Fitbit sensors connected to the Achievement platform.

### Survey Preparation

Survey preparation methods are described in detail in Note S3, and summarized here. Survey cleaning reduced the initial dataset to 146,133 responses from 71,556 unique participants. Since participants could submit multiple survey responses for the same ILI event, distinct ILI events were inferred by merging survey responses from the same participant when the dates encompassing self-reported illness onset through recovery overlapped or were separated by no more than 2 days (chosen to account for potential misreporting of symptom onset date). After excluding participants who reported five or more distinct ILI events and participants who reported multiple distinct COVID-19 events (to remove participants with possible erroneous or fraudulent responses; removed 1,740 participants, bringing the sample to 69,816 among whom 49,397 reported one distinct ILI event throughout the flu season and 20,419 reported more than one ILI event—for whom the most recent ILI event was selected), the analysis set was reduced to a subset of 6,926 ILI events with self-reported clinical diagnoses. Note that applying the thresholding specified above 6 (2.5%) individuals were removed from the COVID-19 cohort. Survey responses were supplemented with demographic information from another survey, including gender, age, BMI, ethnicity, race, and pre-existing health conditions. This survey could have been completed at a different time than the ILI survey, and as such there may be slight discrepancies in time-variant demographic data, such as age, BMI, and health conditions.

### Cohort Definition

To be included in the analysis, participants had to answer “yes” to survey question 10, indicating that they sought medical attention from a healthcare provider for their illness, and either question 12 or 14, indicating that the healthcare provider diagnosed them with either the flu or COVID-19 (see Note S2 for question wordings). Participants who self-reported seeking medical care and being diagnosed with flu and/or COVID-19 by a healthcare provider (n = 6,926) were divided into three cohorts (Figure 2). Participants who completed the amended survey and self-reported being diagnosed with COVID-19 or flu were assigned to the COVID-19 cohort (n = 230) or non-COVID-19 flu cohort (n = 426), respectively. The non-COVID-19 flu cohort offers a comparison of COVID-19 and flu cases that is not confounded by time of year, survey content, time since survey completion, or recent large-scale societal changes, such as shelter-in-place orders or changes within the healthcare system. Participants who reported being diagnosed with flu in the original survey were assigned to the pre-COVID-19 flu cohort (n = 6,270). These cases spanned the 2019–2020 flu season before the COVID-19 outbreak and are included to provide a large comparison group of canonical flu events.

### Wearable Sensor Data Preparation and Analysis

The pipeline for preparing and analyzing the sensor data is described in detail in Note S3 and summarized here. Of the 6,926 participants with diagnosed ILI events, 4,778 (69%) connected at least one wearable sensor to the Achievement platform: 2,582 (37%) participants connected Apple Watches, 2,166 (31%) connected Fitbit sensors, 420 (6%) connected Garmin sensors, 123 (2%) connected Withings sensors, and 17 (0.2%) connected Misfit sensors. We focused the analysis of sensor data on the subset of participants with connected Fitbit sensors, consisting of minute-by-minute steps, heart rate recordings, and sleep states. These data were collected from 2019-11-01 through 2020-05-13. Analyses focused on two different periods: an ILI-event period, conservatively defined as days −10 through +20 relative to self-reported symptom onset (day 0), and a baseline period—all other days before and after the ILI-event period. Given the sparse and often conflicting literature regarding the incubation period and illness duration for COVID-19 that was available at the time analysis was conducted,^61^, ^62^, ^63^, ^64^ the ILI-event period was intentionally wide to capture potential asymptomatic days during the incubation period of the virus (days [−10, −1]) and a potentially long recovery (days [0, 20]). Valid days were defined as those with 10 or more hours of sensor wear time or at least one main sleep period.^65^ The analysis set was restricted to participants with “dense” sensor data, with at least 10% of valid days in the baseline period, and at least 50% of valid days within the ILI event. Dense sensor data were available for 41 COVID-19 patients (36 with steps, 33 with RHR, and 35 with sleep), 85 non-COVID-19 flu patients (80 with steps, 60 with RHR, and 64 with sleep), and 1,226 pre-COVID-19 flu patients (1,193 with steps, 1,025 with RHR, and 979 with sleep). For participants included in the dense wearable analysis cohort, the mean number of missing days was 10.2%, 9.5%, and 8.9% for steps, sleep, and heart rate data, respectively. Sensitivity analysis on the valid day thresholds was conducted and results did not change significantly when removing the requirement of having 10% of valid days for each day of the week, or lowering the percentage of individual valid days to as low as 30%. The pipeline for preparing the wearable data for analysis is illustrated in Figure 1B. While wear time is estimated using minute-by-minute data, all analysis is conducted on day level variables consisting of daily RHR, daily step sum, and nightly sleep hours.

We used a mixed-effects regression model to estimate expected activity levels and adopted the model to impute gaps in RHR values for the RHR-related analysis and to provide counterfactual estimates of “typical” activity during ILI events for the excess analysis. The model specified fixed-effects for the week of the year to control for time of year effects (more specifically, this consisted of three terms for the first, second, and third expansions of an ordinal variable for week of flu season), a categorical fixed-effect for the day of the week to account for differences in activity patterns by day of week, a fixed-effect for the average daily activity level in the participants' state of residence to control for different state-wide shelter-in-place and social distancing measures, and a random-intercept for each participant's baseline activity level to control for individual differences in activity levels. Three models were specified to predict daily total steps, daily RHR, and nightly total sleep time:

$$feature=\beta_{0}+\beta_{1}*week+\beta_{2}*week^{2}+\beta_{3}*week^{3}+\beta_{4}*day\;of\;week+\beta_{5}*state\;mean+u_{0}+\varepsilon .$$

We examined the fraction of each cohort with elevated RHR in the days preceding and following ILI onset.^8^ First, missing RHR values were imputed by fitting the mixed-effects model described previously to all participant-days with an RHR recording. Model estimates were used for days when RHR was not recorded, and observed RHRs were used otherwise. Individualized thresholds for elevated RHR were defined as 1 standard deviation above each participant's mean RHR across all days. The fraction of each cohort with elevated RHR was computed for the days surrounding the ILI event (defined as 10 days before and 20 days after ILI onset). Two-proportion z tests were performed to determine (1) if a greater proportion of the COVID-19 cohort had elevated RHR in the days surrounding ILI onset (days −2 to 2) compared with baseline days before ILI onset (days −10 to −5) and (2) if the proportion of participants with elevated RHR surrounding ILI onset differed among cohorts. Sensitivity analysis was conducted for the elevated RHR analysis, with imputed days being entirely dropped. Results did not change materially, both with respect to the proportion of individuals with elevated RHR, and the trajectory of the elevated RHR traces.

To quantify how COVID-19 and flu impact physical activity, RHR, and sleep, we measured deviations from expected behavior during illness events. We generated individualized estimates of daily measurements that would have been recorded in the counterfactual scenario that the participant did not fall ill (that is, on a typical healthy day) by fitting the mixed-effects model to all participant-days *outside* of the ILI events (all days outside the range of −10 to 20 days from illness onset). Then we computed the *excess*, defined as the difference (observed − estimated), on the days within each ILI event. To visualize the time course of behavioral changes during COVID-19 and flu events (Figure 7), we fit generalized additive mixed models with spline smoothing functions (a thin plate regression spline for days since symptom onset with random effects for participants) to the daily excess time series for each cohort using the mgcv package for R.^66^

## Supplemental Information

Document S1. Figures S1–S3, Tables S1 and S2, and Notes S1–S8

Figure360. An Author Presentation of Figure 5

Document S2. Article plus Supplemental Information
