# Supplementary material for: Characterizing COVID-19 and Influenza Illnesses in the Real World via Person-Generated Health Data
Source: Patterns (N Y). 2020 Dec 13;2(1):100188. doi: 10.1016/j.patter.2020.100188 (PMC7815963; doi:10.1016/j.patter.2020.100188)
Supplement: Document S1. Figures S1–S3, Tables S1 and S2, and Notes S1–S8 [file mmc1.pdf]

PATTER, Volume *2*

Supplemental Information

Characterizing COVID-19 and Influenza Illnesses in the Real World via Person-Generated Health Data

Allison Shapiro, Nicole Marinsek, Ieuan Clay, Benjamin Bradshaw, Ernesto Ramirez, Jae Min, Andrew Trister, Yuedong Wang, Tim Althoff, and Luca Foschini

Supplementary Note 1: Institutional Review Board

This study received expedited review and IRB approval from Solutions IRB (Protocol ID #2018/11/8). Waiver of informed consent was granted by the IRB. Prior to each questionnaire, participants were notified about how their survey responses and behavioral data will be used for research purposes through a disclosure.

Supplementary Note 2: Questionnaire

Weekly 1-Click Item

1. Have you experienced flu-like symptoms in the past 7 days (such as fever, chills, cough, shortness of breath, and/or headache)? If you had flu-like symptoms in the past 7 days, but have recovered, please still answer YES.

   (a) Yes [Symptom Experience Survey]

   (b) No [Infection Risk Factors Survey]

Symptom Experience Survey

1. What is your current zip code? (Where you live and spend the majority of your time) *If you are currently staying in a different location for an extended period of time, please enter your current zip code*.

   (a) numeric 5-digit entry

2. When did you first begin experiencing flu-like symptoms? If you don't recall the exact date, please provide the best estimate.

   (a) calendar date selection

3. As of today, do you feel that you have completely recovered from your illness?

   (a) Yes

   (b) No

[IF Q3 = A, THEN Q4]
[IF Q3 = B, THEN THEN SKIP TO Q5]

4. When did you feel you were completely recovered from your illness? If you don't recall the exact date, please provide the best estimate.

   (a) calendar date selection

5. We'd like to know more about the symptoms you experienced. Looking back over the past 7 days, did you have any of the following symptoms? Please select all that apply.

   (a) Cough

   (b) Body/Muscle Ache

   (c) Fever or feeling feverish

   (d) Chills or shivering

(e) Sweats
(f) Headache
(g) Sore throat or itchy/scratchy throat
(h) Feeling more tired than usual
(i) Nasal congestion or runny nose
(j) Sneezing
(k) I did not experience any flu-like symptoms
(l) Other

[IF Q4 = K, SURVEY END]

6. We'd like to know more about the symptoms you experienced. Looking back over the past 7 days, please indicate on which days you felt the following symptoms.

   (a) Matrix carry forward symptoms; check all that apply: Today, Yesterday, 2 days ago, 3 days ago, 4 days ago, 5 days ago, 6 days ago

7. Looking back over the past 7 days, did you have any of the additional symptoms below

   (a) Shortness of breath and/or difficulty breathing
   (b) Persistent pain or pressure in the chest
   (c) Loss of sense of smell
   (d) None of the above

[IF Q7 = A-C, THEN Q8]
[IF Q7 = D, THEN THEN SKIP TO Q9]

8. Looking back over the past 7 days, please indicate on which days you felt the following additional symptoms.

   (a) Matrix carry forward other symptoms; check all that apply: Today, Yesterday, 2 days ago, 3 days ago, 4 days ago, 5 days ago, 6 days ago

9. Thinking about your flu-like symptoms over the last 7 days, on what day did you feel the worst?

   (a) Today
   (b) Yesterday
   (c) 2 days ago
   (d) 3 days ago
   (e) 4 days ago
   (f) 5 days ago
   (g) 6 days ago

10. Did you seek medical attention from a healthcare provider at a clinic or urgent care facility for this flu or flu-like illness?

   (a) Yes
   (b) No

[IF Q10 = A, THEN Q11]
[IF Q10 = B, THEN SKIP TO Q23]

11. Where did you seek care from a healthcare provider?

    (a) Primary care clinic (e.g. family medicine, internal medicine)
    (b) Urgent care facility
    (c) Emergency room (ER)
    (d) Ear, nose, and throat (otolaryngology) clinic
    (e) Infectious disease clinic
    (f) Other

12. Did the healthcare provider diagnose you as having the flu?

    (a) Yes
    (b) No
    (c) I don't know / I can't remember

13. Did the healthcare provider perform any of the following tests? Select all that apply.

    (a) Nasal swab
    (b) Throat swab
    (c) Symptoms only (no lab test)
    (d) I don't know / I can't remember
    (e) Other (please specify)

14. Did the healthcare provider diagnose you as having coronavirus disease (also known as COVID-19)?

    (a) Yes
    (b) No
    (c) I am waiting for my diagnosis
    (d) I don't know / I can't remember

15. Did you take any of the following tests for your coronavirus diagnosis? Select all that apply.

    (a) Nasal swab
    (b) Throat swab (c) Blood test
    (d) Spit test / kit
    (e) Symptoms only (no lab test)
    (f) I don't know / I can't remember
    (g) Other (please specify)

16. Where did you take the COVID-19 diagnostic test?

    (a) In a clinic or hospital
    (b) At a drive through testing facility
    (c) At home testing kit
    (d) Other (please specify)
    (e) None of the above

17. Were you hospitalized as a consequence of this flu or flu-like illness? *Hospitalization is when you leave the emergency room (ER) and are admitted to the inpatient hospital based on a doctor's order. Even if you stayed overnight in the ER, this is not considered a hospitalization*.

    (a) Yes (b) No

18. Were you told to self-quarantine (stay in your home without leaving for any reason) by a medical professional?

    (a) Yes
    (b) No
    (c) I don't know / I can't remember

19. Did a healthcare provider prescribe any medications to treat or manage your current symptoms?

    (a) Yes
    (b) No
    (c) I don't know / I can't remember

[IF Q19 = A, THEN Q20]

[IF Q19 = B, THEN SKIP TO Q23]

20. Which of the following medications were you prescribed to treat or manage your symptoms? Select all that apply.

    (a) Xofluza (baloxavir marboxil)
    (b) Tamiflu (oseltamivir)
    (c) Relenza (zanamivir)
    (d) Antibiotics (Z-pak, amoxicillin, Augmentin, doxycycline) (e) Other

21. When did you take your first dose of [CARRY FORWARD MEDICATION NAME]? Please enter the date in MM/DD/YYYY format.

    (a) Date entry

22. Did you ever miss any doses or decide not to take [CARRY FORWARD MEDICATION NAME]? a.

    (a) I missed at least one dose of this medication
    (b) I did not take any doses of this medication
    (c) I did not miss any doses of medication
    (d) I don't know / I can't remember

23. Did you take any over-the-counter (non-prescription) medications to treat or manage your current symptoms in the past 7 days?

    (a) Yes
    (b) No
    (c) I don't know / I can't remember

[IF Q23 = A, THEN Q24]

[IF Q23 = B, THEN SKIP TO Q25]

24. Which of the following over-the-counter (non-prescription) medications did you personally decide to take to treat or manage your current symptoms in the past 24 hours? Select all that apply.

(a) Fever reducers or pain relievers (ibuprofen, aspirin, Advil, Tylenol, Aleve, acetaminophen)

(b) Cough suppressants (Delsym, Robitussin, dextromethorphan)

(c) Chest or mucus decongestants (Mucinex, guaifenesin)

(d) Nasal decongestants (Sudafed, Sudafed PE, Afrin, Flonase, phenylephrine, pseudoephedrine, fluticasone propionate)

(e) I don't know / can't remember

(f) Other

25. How many people (other than yourself) live in your household?

(a) 0

(b) 1

(c) 2

(d) 3

(e) 4 (f) 5

(g) 6

(h) 7

(i) 8

(j) 9

(k) 10

(l) >10

26. Have any members of your household (other than yourself) experienced flu-like illness this flu season?

(a) Yes

(b) No

(c) I live alone

[IF Q26 = A, THEN Q27]

[IF Q26 = B or C, THEN SKIP TO Q29]

27. How many members of your household, by age group listed below, have experienced flu-like symptoms during this flu season (September 2019 to today)? If no household member in your household experienced symptoms within an age group please enter 0. [numeric entry]

(a) Number of household members 0-4 years old experiencing flu-like symptoms

(b) Number of household members 5-17 years old experiencing flu-like symptoms

(c) Number of household members 18-49 years old experiencing flu-like symptoms

(d) Number of household members 50-64 years old experiencing flu-like symptoms

(e) Number of household members 65+ years old experiencing flu-like symptoms

28. Have any members of your household been diagnosed with coronavirus disease (also known as COVID-19?

(a) Yes

(b) No

29. Have you been in close contact with anyone outside your household (e.g., family members, friends, coworkers, acquaintances) who has experienced flu-like symptoms recently? *Close contact can include*

direct physical contact, face-to-face contact for longer than 15 minutes, exchange of bodily fluids, or being within 6 feet of the person for more than 15 minutes.

(a) Yes, within the last 7 days
(b) Yes, within the last 14 days
(c) Yes, over 14 days ago
(d) No
(e) I don't know / I'm not sure

30. Have you recently been in contact with someone who was diagnosed with coronavirus? *Close contact can include direct physical contact, face-to-face contact for longer than 15 minutes, exchange of bodily fluids, or being within 6 feet of the person for more than 15 minutes*.

    (a) Yes, within the last 7 days
    (b) Yes, within the last 14 days
    (c) Yes, over 14 days ago
    (d) No
    (e) I don't know / I'm not sure

31. Did you miss school or work due to your illness?

    (a) No, I did not miss any school or work during my illness
    (b) I missed 1 day of school or work
    (c) I missed 2 days of school or work
    (d) I missed 3 days of school or work
    (e) I missed more than 3 days of school or work
    (f) Illness occurred on a weekend or other day(s) off
    (g) I am retired and/or school or work days don't apply to me
    (h) I don't know / I don't remember

32. Looking back over the past 7 days, which days have you practiced social distancing or isolation behaviors (e.g., working remotely, limited the time spent in crowds, increasing the amount of time spent at home)? Please select all that apply.

    (a) Today
    (b) Yesterday
    (c) Two days ago
    (d) Three days ago
    (e) Four days ago
    (f) Five days ago
    (g) Six days ago
    (h) I did not practice social distancing in the last 7 days

33. Did you receive the flu vaccine (sometimes called the flu shot) this flu season (September 2019 to today)?

    (a) Yes
    (b) No

(c) I don't know / I can't remember

34. Did you receive the flu vaccine last flu season? (September 2018 - March 2019)

    (a) Yes
    (b) No
    (c) I don't know / I can't remember

35. Please select the statement below that describes whether you typically get a flu shot (or another form of flu vaccine).

    (a) I never have gotten a flu shot
    (b) I rarely get a flu shot
    (c) I get a flu shot every year
    (d) I sometimes get a flu shot

36. Have you recently traveled on an airplane?

    (a) Yes, within the last 7 days
    (b) Yes, within the last 14 days
    (c) Yes, over 14 days ago
    (d) No

37. Have you recently participated in any large public gatherings of over 250 people (e.g., concerts, sporting events, amusement parks)?

    (a) Yes, within the last 7 days
    (b) Yes, within the last 14 days
    (c) Yes, over 14 days ago
    (d) No

38. Are you or one of your household members a healthcare worker (i.e., doctor, dentist, nurse, nurse's aid, paramedic, physician's assistant, home healthcare aid, hospital worker, pharmacist, or other type of healthcare worker)? Please select all that apply.

    (a) I am, and I am currently working
    (b) I am, but I am NOT currently working
    (c) One of my household members is, and they are currently working
    (d) One of my household members is, but they are NOT currently working
    (e) No one in my household is a healthcare worker

39. Were you diagnosed by a healthcare provider with any of the following health problems either during your flu illness or since you recovered from your flu illness? Please select all that apply. [CHECKBOX]

    a. Chest infection (pneumonia, acute lung injury)
    b. Worsening of asthma or COPD
    c. Bloodstream infection (sepsis)
    d. Ear infection (otitis media)
    e. Sinus infection (sinusitis)
    f. Brain inflammation (encephalitis or encephalopathy)

g. Stroke
h. Inflammation of the heart or the covering of the heart (myocarditis and/or pericarditis)
i. Worsening of heart failure
j. Heart attack (myocardial infarction)
k. Muscle inflammation (myositis or rhabdomyolysis)
l. No, I was not diagnosed with any of these conditions

[IF Q38 = YES TO ANY → GO TO Q39 for each complication selected
IF Q38 = “l” —> end survey]]

40. Approximate date that the [CARRY FORWARD COMPLICATION SELECTIONS] first occurred. Please enter the date in MM/DD/YYYY format.
    a. Date text entry

Supplementary Note 3: Methods

Survey Filtering.

Survey responses with self-reported illness onset dates or recovery dates that occurred 30 or more days before the survey completion date were excluded, leaving 158,999 survey responses from 73,728 unique participants. Survey responses with invalid illness onset and/or recovery dates (defined as dates occurring after the survey date or responses in which the illness recovery date occurred before the illness onset date) were also removed, leaving 149,309 survey responses from 71,556 unique individuals. Finally, the set of survey responses was restricted to one survey per participant per day. If one participant attempted more than one survey in a given day, the less complete survey was excluded.

Inference of Distinct ILI Events.

Participants could submit new survey responses as frequently as once per week, with no maximum limit. Therefore, individual symptom trajectories for an ILI event had to be inferred by concatenating and reconciling multiple surveys responses, for example, if the participant were midway through their illness when they submitted their first survey their next survey could describe the second half of their illness.

We inferred ILI events by merging multiple surveys from the same participant with date ranges encompassing symptoms onset and recovery that overlapped or were separated by no more than 2 days. Participants with more than 5 ILI events were removed, eliminating 16,878 surveys and 1,639 participants, and leaving 126,014 survey responses, corresponding to 99,604 distinct ILI events and 69,034 participants.

This gives the set of discrete ILI events per participant, from which we will select only 1 for analysis. If a participant has a diagnosed COVID-19 ILI event, that event is selected, otherwise the most recent ILI event is selected. This process removes a further 40,357 surveys and 30,567 distinct ILI events, and 0 participants. Participants reporting multiple non-overlapping

diagnosed COVID-19 events were then excluded (excluding 7 survey responses, 6 distinct ILI events, and 3 participants).

Reconciliation of Merged Survey Responses.

At this point, we have one ILI event per participant, corresponding to 85,650 surveys for 69,031 distinct ILI events across 69,031 participants. We then reconcile responses to derive a single value per item. For example, the date of onset and recovery are taken as the earliest and latest reported date for that ILI event, respectively.

Participants who reported being diagnosed with both flu and COVID-19 (N=83) were assigned to the COVID-19 cohort, under the rationale that some individuals may consider COVID-19 to be a type of flu, and the relative order in the questionnaire (flu preceding COVID-19). Among the 41 COVID-19 cases with dense Fitbit data, 14 also reported having the flu (12 with dense HR data, 12 with dense sleep data, 11 with dense steps data.

Flu events drawing from multiple surveys responses may have differing symptoms reports for the same calendar date. Such day-level values (e.g., symptoms reported for a specific day) were collapsed if identical, and if not, the survey submitted on the date closest to the calendar date was used. Participants were also allowed to report annotations, for example "the worst day", during a given event. These are highly subjective, thus all responses were retained, with a given date coded as "one of the worst days" if the participant indicated as such in any survey. For event-level categorical features, the algorithm described in Figure S1 was used to collapse surveys to a single response. Numerical event-level features, for example the number of household members who have experienced ILI symptoms, were aggregated by taking the maximum value reported. All other features which could not be reconciled were simply aggregated as concatenated unique values.

In our selection of ILI events,we select COVID-19 events or the most recent ILI event for each participant. This biases our analysis towards later calendar dates when sensor data is most affected by social distancing. For this reason, we have included a chronologically parallel group of Non-COVID-19 Flu patients. A second issue is that we could be missing participants' most severe ILI events, which could have happened earlier in the season. We will continue to monitor symptomatic and behavioral changes associated with COVID-19 and non-COVID-19 ILIs as more events are captured and as guidance on social distancing and stay-at-home measures are relaxed. Further analysis will focus on how strongly these measures confound our observations.

Statistical Testing.

A two-step statistical testing procedure was used to test for differences in demographics, healthcare care-seeking behavior, medical outcomes, and symptoms among the three cohorts. First, for each sub-analysis (i.e., demographics, medical care-seeking, and symptom prevalence), a series of chi-squared tests of independence were performed to test for an association between the three cohorts and the different possible outcomes for each category. A Bonferroni correction was applied to adjust for running multiple chi-squared tests in each sub-analysis. Second, follow-up two-proportion z-tests were performed to test for differences in proportions for each outcome and each pair of cohorts. These follow-up tests were only

performed for the categories with significant cohort differences as determined by the chi-squared tests.

Wearable Sensor Data Preparation.

The pipeline for preparing the surveys for analysis is described in detail in Supplementary Note 3 and summarized here.

Of the 6,926 participants with diagnosed ILI events, 4,778 (69%) have shared at least one wearable device connected to the Achievement platform: 2,582 (37%) participants had connected Apple Watches, 2,166 (31%) had connected Fitbit devices, 420 (6%) had connected Garmin devices, 123 (2%) had connected Withings devices, and 17 (0·2%) had connected Misfit devices. We focus the analysis of sensor data on the subset of participants with connected Fitbit devices, consisting of minute-by-minute steps, heart rate recordings, and sleep states, available for a subset of study participants. This data was collected from 2019-11-01 through 2020-05-13 and analyzed to investigate the impact of COVID-19 and flu on everyday behavior and physiology.

Since the sensor data was collected passively in real-world settings, daily sensor wear-time varied across participants and study days. We implemented a three step procedure to enforce adequate data density around each ILI event prior to analysis. First, we estimated if the sensor was worn for each participant for each minute in the study period. Periods of non-wear-time were defined as 180 or more consecutive minutes of zero steps or null heart rate recordings. Second, days with 10 or more hours of sensor wear-time were tagged as valid for analysis. For the sleep data, days with at least one main sleep period recorded by Fitbit were considered valid. Third, the analysis set was restricted to only include participants with 1) at least 10% of valid days for each day of the week in the baseline period (defined as all participant-days that occurred outside the window of 10 days prior to and 20 days after illness onset) and 2) at least 50% valid days in the time period surrounding the ILI event (defined as all days within the window of 10 days prior to 20 days after illness onset). Dense sensor data was available for 41 COVID-19 patients (36 with steps, 33 with RHR, and 35 with sleep), 85 Non-COVID-19 Flu patients (80 with steps, 60 with RHR, and 64 with sleep), and 1226 Pre-COVID-19 Flu patients (1193 with steps, 1025 with RHR, and 979 with sleep). Sensitivity analysis on the valid day thresholds was conducted and results did not change significantly when removing the requirement of having 10% of valid days for each day of week or lowering the percentage of individual valid days to as low as 30%. The pipeline for preparing the wearable data for analysis is illustrated in Supplementary Figure S1 (b).

Elevated RHR Prevalence.

Similarly to previous work,[1] we examined the fraction of each cohort with elevated RHR in the days preceding and following ILI onset. First, days without RHR recordings were imputed in order to ensure that the cohorts were the same across days of interest. Imputed RHR values were generated from predictions of a mixed effects regression model that was fit to all participant-days that RHR was recorded. The model specified fixed effects for the week of the year to control for time of year effects (more specifically, this consisted of three terms for the

1st, 2nd, and 3rd expansions of an ordinal variable for week of flu season), a categorical fixed effect for the day of the week to account for differences in activity patterns by day of week, a fixed effect for the average activity level in the participants' state of residence to control for different state-wide shelter-in-place and social distancing measures, and a random intercept for each participant's baseline activity level to control for individual differences in activity levels. The model was fit to all participant-days with a RHR recording using the lme4 package for R.[2] Note that the imputed values were used only to fill days when RHR was not recorded, the observed value was used on all other days .
Next, in order to account for individual differences in RHR when defining thresholds for elevated RHR, RHR values were converted to z-scores using each participant's RHR mean and standard deviation across all days. The fraction of each cohort with elevated RHR was computed for the days surrounding the ILI event, defined as 10 days prior to 20 days after ILI onset. Elevated RHR was defined as being greater than 1 standard deviation above the participant's mean RHR. Two-proportion z-tests were performed to answer the following two questions: 1. Does a greater fraction of the COVID-19 cohort have elevated RHR in the days surrounding ILI onset compared to days prior to ILI onset and 2. Does the fraction of participants with elevated RHR surrounding ILI onset differ between COVID-19 and Flu cohorts? The time window surrounding ILI onset was defined as starting two days prior to self-reported illness onset and ending two days afterward (Days -2 to 2). We conservatively allowed 2 days for physiological changes before any symptom was reported, and 2 days after the onset of symptoms as the time horizon within which actions could be taken that would not otherwise be taken without information from the wearable device. For the purpose of the first statistical test, the time period prior to ILI onset was defined as Days -10 to -5 relative to self-reported illness onset. This time window was selected because it fell within the time period that data density was enforced, it was the same duration as the time window surrounding ILI onset, and the median time from exposure to COVID-19 to the development of symptoms is 5 days[3].

Behavioral and Physiological Changes During ILI Events.

In order to characterize daily changes associated with COVID-19 and flu events, we measured deviations from typical healthy measurements (RHR, step count, sleep hours) that occurred while participants were ill. We used a model on symptom-free days (conservatively assumed all days excluding the 10 days before symptoms onset and within 20 days after symptoms onset) to generate individualized estimates of daily measurements that would have been recorded in the counterfactual scenario that the participant did not fall ill, and then computed the excess, defined as the difference (observed - estimated), on the days surrounding symptoms onset, and finally report the excess as a measure of deviations from expected typical measurements. The symptom-free day model was a mixed effects regression model with the same specification as what was used to impute missing RHR values in the previous analysis. The key difference in this analysis was that, in order to generate estimates based only on assumed symptom-free days, we excluded all data within 10 days before symptoms onset and within 20 days after symptoms onset when fitting the model. In order to visualize the time course of behavioral changes during COVID-19 and flu events, we fit generalized additive mixed models with spline

smoothing functions and random intercepts to the daily excess time series for each cohort using the mgcv package for R.[4] This procedure was performed three separate times, for each of the channels considered: daily total step counts, daily RHR, and total daily sleep minutes.

Supplementary Note 4: Comorbidity Prevalence

Table S1 describes self-reported comorbidities observed in our ILI cohorts.

	COVID-19	Non-COVID-19 Flu	Pre-COVID-19 Flu
Anxiety	65 (28.3%)	122 (28.6%)	1915 (30.5%)
Depression	62 (27.0%)	104 (24.4%)	1868 (29.8%)
Asthma	56 (24.3%)	79 (18.5%)	1247 (19.9%)
Migraines	50 (21.7%)	75 (17.6%)	1225 (19.5%)
Chronic Pain	30 (13.0%)	40 (9.4%)	572 (9.1%)
Hypertension	17 (7.4%)	46 (10.8%)	718 (11.5%)
PCOS	16 (7.0%)	16 (3.8%)	315 (5.0%)
GERD	15 (6.5%)	46 (10.8%)	599 (9.6%)
Mental Health (Excluding Depression/Anxiety)	15 (6.5%)	32 (7.5%)	499 (8.0%)
Insomnia	15 (6.5%)	37 (8.7%)	541 (8.6%)
Sleep Apnea	11 (4.8%)	20 (4.7%)	335 (5.3%)
Restless Leg Syndrome	10 (4.3%)	12 (2.8%)	231 (3.7%)
Type 2 Diabetes	10 (4.3%)	10 (2.3%)	254 (4.1%)
Hypo- or Hyperthyrodism	9 (3.9%)	32 (7.5%)	418 (6.7%)
Fibromyalgia	9 (3.9%)	16 (3.8%)	215 (3.4%)
High Cholesterol	9 (3.9%)	21 (4.9%)	352 (5.6%)
Gestational Diabetes	8 (3.5%)	13 (3.1%)	187 (3.0%)
Cancer	6 (2.6%)	13 (3.1%)	165 (2.6%)

Arrhythmia	6 (2.6%)	5 (1.2%)	165 (2.6%)
Psoriasis	5 (2.2%)	14 (3.3%)	147 (2.3%)
Type 1 Diabetes	4 (1.7%)	6 (1.4%)	63 (1.0%)
Rheumatoid Arthritis	4 (1.7%)	7 (1.6%)	135 (2.2%)
Stroke	3 (1.3%)	3 (0.7%)	31 (0.5%)
Heart Attack	2 (0.9%)	3 (0.7%)	28 (0.4%)
IBS or IBD	2 (0.9%)	9 (2.1%)	94 (1.5%)
COPD	2 (0.9%)	4 (0.9%)	61 (1.0%)
Seasonal Allergies	2 (0.9%)	5 (1.2%)	109 (1.7%)
Lupus	1 (0.4%)	0 (0.0%)	7 (0.1%)
Coronary Heart Disease	1 (0.4%)	0 (0.0%)	13 (0.2%)
Multiple Sclerosis	1 (0.4%)	4 (0.9%)	30 (0.5%)
Alzheimer’s Disease	1 (0.4%)	1 (0.2%)	3 (0.0%)
Heart Failure	0 (0.0%)	5 (1.2%)	27 (0.4%)
Neurodegenerative	0 (0.0%)	1 (0.2%)	4 (0.1%)
Arthritis	0 (0.0%)	3 (0.7%)	37 (0.6%)
Osteoporosis	0 (0.0%)	6 (1.4%)	51 (0.8%)

Table S1. Prevalence of self-reported co-morbidities for the COVID-19 (N=230), Non-COVID-19 Flu (N=426), and Pre-COVID-19 Flu (N=6270) cohorts.

Supplementary Note 5: Symptom Labels

Table S2 describes the labels and associated descriptions used in our surveys for this work.

Symptom Label	Symptom Description in Survey
Cough	Cough
Headache	Headache

Body Muscle Ache	Body/Muscle Ache
Fatigue	Feeling more tired than usual
Fever	Fever or feeling feverish
Chills or Shivering	Chills or shivering
Sore Throat	Sore throat or itchy/scratchy throat
Nasal Congestion	Nasal congestion or runny nose
Sweats	Sweats
Sneezing	Sneezing
Chest Pain/Pressure	Persistent pain or pressure in the chest
Shortness of Breath	Shortness of breath and/or difficulty breathing
Anosmia	Loss of sense of smell

Table S2. Full symptom descriptions included in the survey for each abbreviated symptom label. The Chest Pain/Pressure, Shortness of Breath, and Anosmia symptoms were only included in the updated survey.

Supplementary Note 6: Symptom Reporting

Figure S1 describes the percentage of each ILI cohort reporting daily symptoms between one week prior and 4 weeks post symptom onset. Figure S2 describes the percentage of observed symptom reporting for hospitalized and non-hospitalized COVID-19 cohorts, between one week prior and 4 weeks post symptom onset.

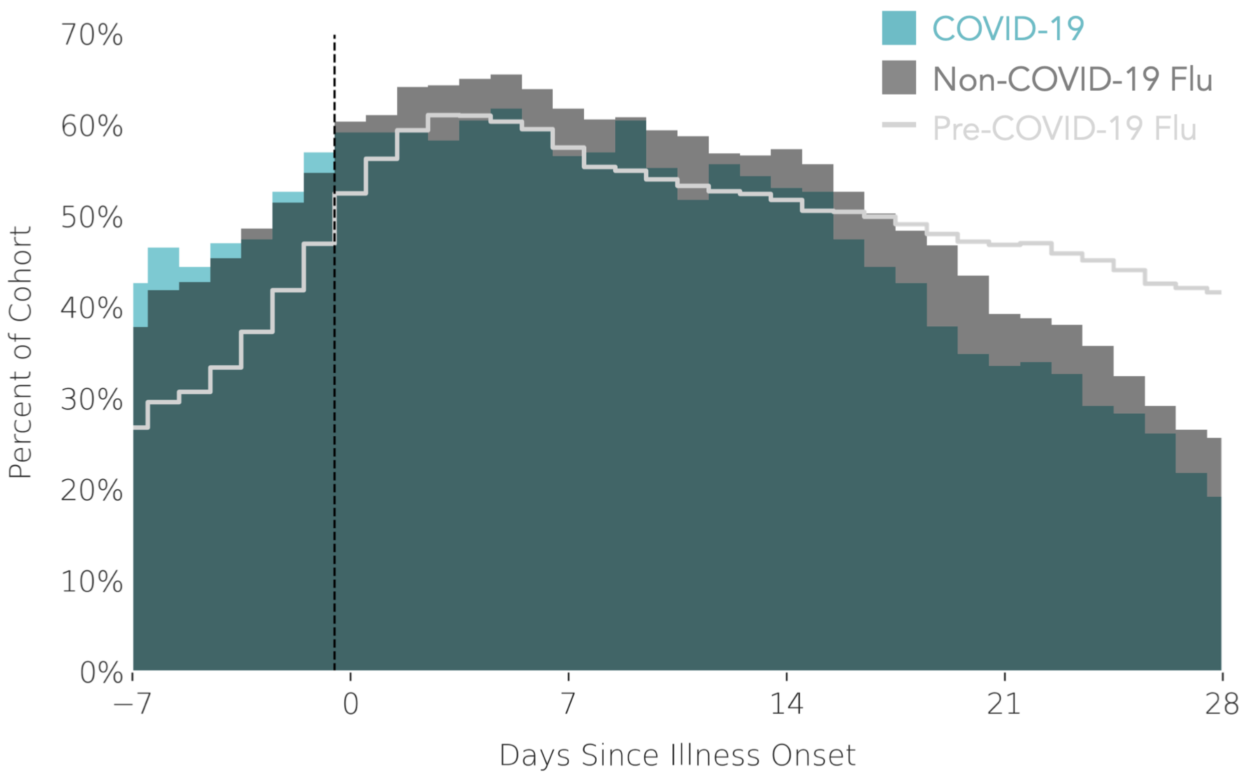

Fig. S1. Percentage of COVID-19 (N=230; blue), Non-Covid Flu (N=426; gray), and Pre-Covid Flu (N=6270, light gray trace) cohorts with symptom reports for days -7 to 28 since illness onset.

We note that our approach may underestimate disease severity, due to participants not reporting symptoms, not wearing sensors in days when symptoms are most severe, or during hospitalization events (see Figure S3).

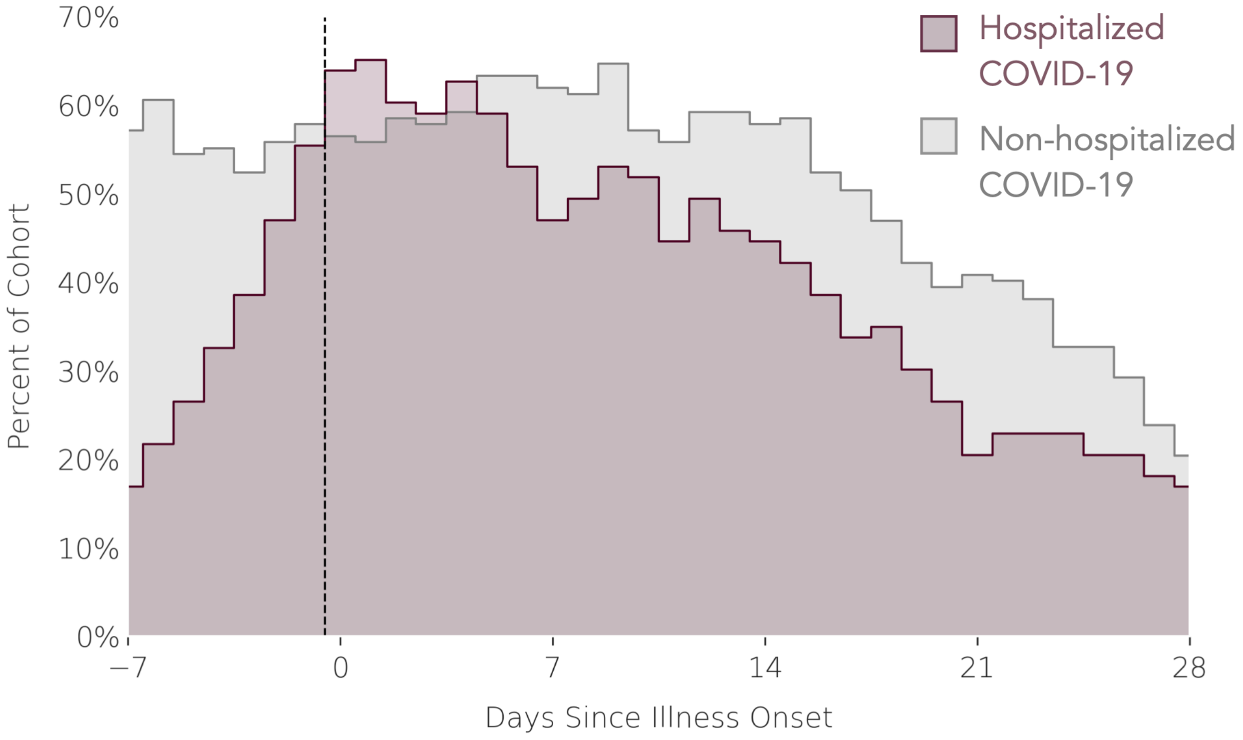

Fig. S2. Percentage of the Hospitalized (N=83, purple) and Non-hospitalized (N=147, gray) COVID-19 sub-cohorts with symptom reports for days -7 to 28 since illness onset.

Supplementary Note 7: Sensor Data Coverage

A summary of coverage of wearable sensor data over the course of the study is visualized in Figure S3.

We recognize that our analyses do not immediately translate to real-time implementation of COVID-19 monitoring, due to lag in data collection that comes from sensor and data synchronization. Lags in our dataflow are nevertheless small compared to the gains in symptom detection and reporting compared to canonical practice.

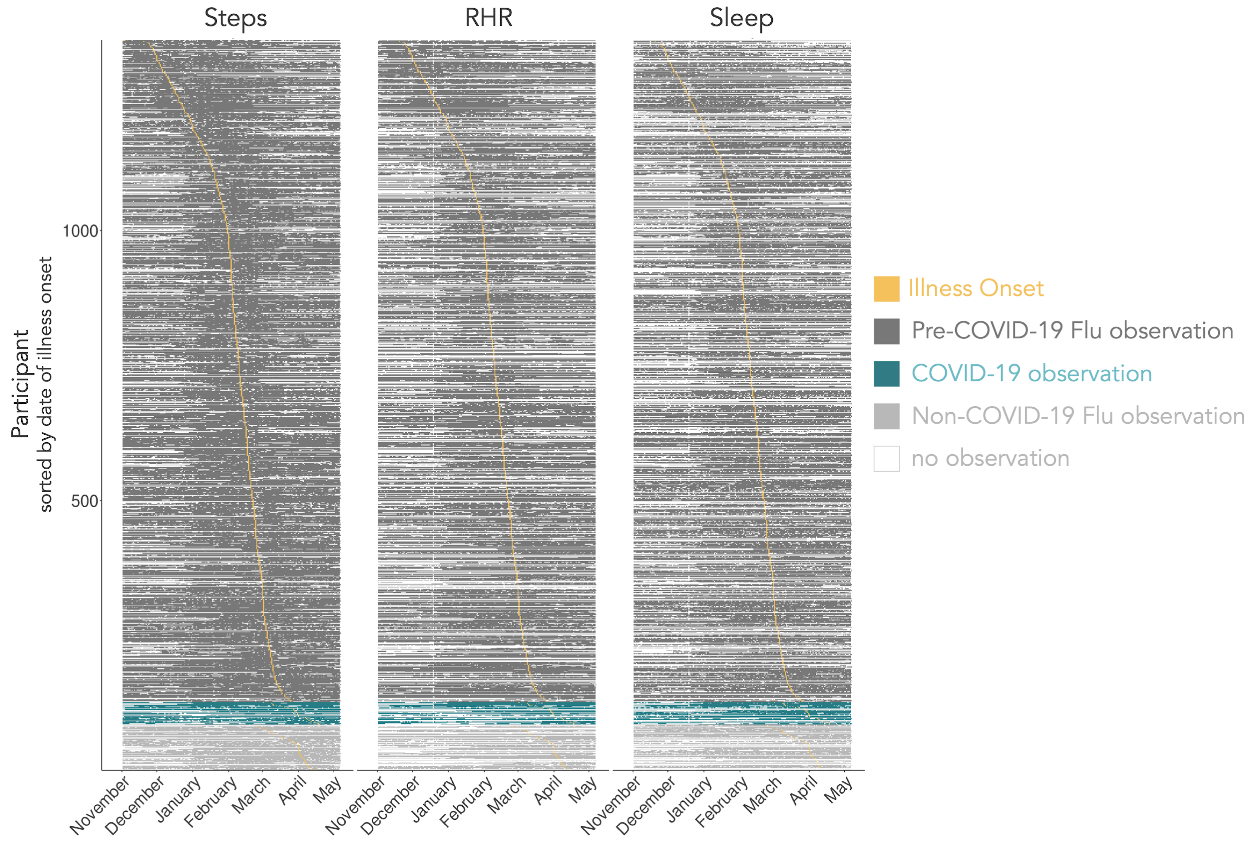

Fig. S3. Coverage of Fitbit steps, sleep, and RHR data on each calendar date of the study, color-coded by cohort. Each row is one participant (ordered by date of ILI-onset) and each column is one calendar date. Shaded days indicate that wearable data was recorded on that day from that participant. Days highlighted in yellow indicate the ILI onset dates.

Supplementary Note 8: Data Availability

De-identified study data will be made available to qualified researchers on the Sage Synapse platform[5] in September 2020.

Supplementary references

1. Radin, J.M., Wineinger, N.E., Topol, E.J., and Steinhubl, S.R. (2020). Harnessing wearable device data to improve state-level real-time surveillance of influenza-like illness in the USA: a population-based study. The Lancet Digital Health 2, e85–e93. Available at: https://www.sciencedirect.com/science/article/pii/S2589750019302225/pdfft?md5=785c228a82

10aa6e9a68aaa482f2274d&pid=1-s2.0-S2589750019302225-main.pdf [Accessed June 2, 2020].

2. Bates, D., Mächler, M., Bolker, B., and Walker, S. (2015). Fitting Linear Mixed-Effects Models Using lme4. J. Stat. Softw. 67. Available at: http://www.jstatsoft.org/v67/i01/.

3. Lauer, S.A., Grantz, K.H., Bi, Q., Jones, F.K., Zheng, Q., Meredith, H.R., Azman, A.S., Reich, N.G., and Lessler, J. (2020). The Incubation Period of Coronavirus Disease 2019 (COVID-19) From Publicly Reported Confirmed Cases: Estimation and Application. Ann. Intern. Med. 172, 577–582. Available at: http://dx.doi.org/10.7326/M20-0504.

4. Wood, S.N. (2011). Fast stable restricted maximum likelihood and marginal likelihood estimation of semiparametric generalized linear models. Journal of the Royal Statistical Society: Series B (Statistical Methodology) 73, 3–36. Available at: https://rss.onlinelibrary.wiley.com/doi/abs/10.1111/j.1467-9868.2010.00749.x [Accessed June 2, 2020].
