# Supplementary material for: Characterizing COVID-19 and Influenza Illnesses in the Real World via Person-Generated Health Data
Source: Patterns (N Y). 2020 Dec 13;2(1):100188. doi: 10.1016/j.patter.2020.100188 (PMC7815963; doi:10.1016/j.patter.2020.100188)
Supplement: Document S2. Article plus Supplemental Information [file mmc3.pdf]

Article

Patterns

Characterizing COVID-19 and Influenza Illnesses in the Real World via Person-Generated Health Data

Highlights

- We use data from smartphones and wearables from ~7,000 people to compare flu and COVID-19
- While symptoms have some overlap, patients report longer COVID-19 illnesses than flu
- Elevated resting heart rate measures are more frequent around ILI symptoms onset
- It is important to consider flu as a confounder in COVID-19 real-world studies

Authors

Allison Shapiro, Nicole Marinsek, Ieuan Clay, ..., Yuedong Wang, Tim Althoff, Luca Foschini

Correspondence

luca@evidation.com

In Brief

Person-generated health data (PGHD), including data from smartphones and other connected sensors, has the potential to enable applications ranging from individual-level early warnings or population-level hotspot detection for COVID-19. We show that, when studied outside the clinic, COVID-19 and flu may have overlapping, yet distinct, symptom presentations. For COVID-19 PGHD applications to become a reality, our findings suggest that it is crucial to develop and validate them in the context of other potentially confounding respiratory illnesses, such as the flu.

Shapiro et al., 2021, Patterns 2, 100188
January 8, 2021 © 2021 The Authors.
https://doi.org/10.1016/j.patter.2020.100188

CellPress

Patterns

CellPress
OPEN ACCESS

Article

Characterizing COVID-19 and Influenza Illnesses in the Real World via Person-Generated Health Data

Allison Shapiro,[1,5] Nicole Marinsek,[1,5] Ieuan Clay,[1] Benjamin Bradshaw,[1] Ernesto Ramirez,[1] Jae Min,[1] Andrew Trister,[2] Yuedong Wang,[3] Tim Althoff,[4] and Luca Foschini[1,6,*]
[1]Evidation Health, Inc., San Mateo, CA 94401, USA
[2]Bill and Melinda Gates Foundation, Seattle, WA 98109, USA
[3]Department of Statistics and Applied Probability, University of California Santa Barbara, Santa Barbara, CA 93106, USA
[4]Allen School of Computer Science & Engineering, University of Washington, Seattle, WA 98115, USA
[5]These authors contributed equally
[6]Lead Contact
*Correspondence: luca@evidation.com
https://doi.org/10.1016/j.patter.2020.100188

THE BIGGER PICTURE In this study, we integrate longitudinal symptoms reports and continuous data from commercial wearables to compare and contrast flu and COVID-19 presentations. We found that, while symptoms constellation between COVID-19 and flu have large overlap, symptoms are significantly more prolonged and severe for COVID-19 than for flu. Similarly, physiological data from commercial wearables showed increased resting heart rate around symptoms onset date that were more severe for COVID-19, but present in milder form for flu as well.

Person-generated health data (PGHD), including data from smartphones and other connected sensors, has the potential to enable applications ranging from individual-level early warnings or population-level hotspot detection for COVID-19. However, for these applications to become a reality, our findings suggest that it is crucial to develop and validate them in the context of other potentially confounding respiratory illnesses, such as the flu.

1 2 3 4 5 **Proof-of-Concept:** Data science output has been formulated, implemented, and tested for one domain/problem

SUMMARY

The fight against COVID-19 is hindered by similarly presenting viral infections that may confound detection and monitoring. We examined person-generated health data (PGHD), consisting of survey and commercial wearable data from individuals' everyday lives, for 230 people who reported a COVID-19 diagnosis between March 30, 2020, and April 27, 2020 (n = 41 with wearable data). Compared with self-reported diagnosed flu cases from the same time frame (n = 426, 85 with wearable data) or pre-pandemic (n = 6,270, 1,265 with wearable data), COVID-19 patients reported a distinct symptom constellation that lasted longer (median of 12 versus 9 and 7 days, respectively) and peaked later after illness onset. Wearable data showed significant changes in daily steps and prevalence of anomalous resting heart rate measurements, of similar magnitudes for both the flu and COVID-19 cohorts. Our findings highlight the need to include flu comparator arms when evaluating PGHD applications aimed to be highly specific for COVID-19.

INTRODUCTION

The emergence of the novel SARS-CoV-2 (COVID-19) pandemic necessitates an understanding of symptom prevalence and progression among individuals with COVID-19, as well as how COVID-19 symptoms compare with those of other infectious diseases. Self-reported data collected at the point of care are being used to help answer key questions around the management of COVID-19 patients,[1] and real-world data collected via smartphone apps from individuals participating in COVID-19

This is an open access article under the CC BY license (http://creativecommons.org/licenses/by/4.0/).

syndromic surveillance programs[2–4] are being used to perform population-level hotspot detection,[5] and show promise in understanding symptom presentation outside clinic walls. In addition to self-report, data from commercial sensors may be used for large-scale surveillance of influenza-like illnesses (ILI), given that resting heart rate (RHR)[6–10] and temperature[11] change in the presence of an infection. Benefits may come from integrating different digital data sources. For example, a hotspot detection system, including smart thermometers and internet searches, has been shown to provide accurate early-warning indicators of increasing or decreasing state-level US COVID-19.[12] Syndromic surveillance based on symptom self-report has recently been shown to scale to tens of thousands of responses per day,[5] and wearables sensors, being worn by one in five Americans,[13] could further increase the volume of daily feeds of person-generated health data (PGHD) used at the aggregate level for syndromic surveillance and hotspot detection.

In addition to being used in aggregate form for population-level hotspot detection, PGHD is also being proposed as a candidate for individual-level applications, such as to support detection and monitoring of COVID-19 and other respiratory viruses.[14–18]

Along these lines, several efforts are currently underway to explore the potential of using wearable technology to detect COVID-19 onset,[19–21] and preliminary results have shown that wearables may be able to predict COVID-19 symptoms before onset,[22–24] with potential application to large-scale, low-sensitivity/high-frequency testing to enable reopening in the wait for a vaccine.[14–16]

However, the lack of a canonical COVID-19 symptom presentation, including how symptoms progress over time,[24,25] undermines our ability to track, predict, and control disease progression and manage critical care. In addition, to evaluate performances of any detection system, being that for individual-level early warnings or population-level hotspot detection, it is crucial to compare and contrast symptoms, behavioral, and physiological manifestation with other ILIs, especially flu. Most current COVID-19 research has been developed outside of flu season, but will have to withstand confounding from a surge of flu cases as flu season escalates. This remains true even in spite of the fact that the flu season is expected to be milder due to lockdown measures,[26] as lockdowns are merely bringing flu prevalence within the same order of magnitude as COVID-19 prevalence.

To address this challenge, we present PGHD contemporaneously shared by individuals who self-reported being diagnosed by a medical provider with either flu or COVID-19. We also present PGHD from a comparator group who were diagnosed with flu before the COVID-19 pandemic. The PGHD consists of self-reported surveys describing symptoms and experiences, and sensor-derived continuous data describing behavior and physiology. The PGHD were collected as part of a large-scale digital participatory surveillance study designed to monitor ILI over the 2019–2020 influenza season. Wearable sensor PGHD (including daily RHR, step counts, and nightly sleep hours), allows us to link continuously measured behavioral and physiological patterns to illness onset.

Our contributions are 2-fold: first, we examine the presentation of COVID-19 symptoms outside of strictly clinical settings both in terms of constellation and time course, and contextualize them with comparisons with seasonal influenza; second, by analyzing wearable data around symptoms onset we show that physiological signals, such as RHR change significantly near symptom onset, as do physical activity measures, such as step counts, although these changes appear to be similar in timing and magnitude across ILI and COVID-19 cohorts. Beyond furthering understanding of mild-to-moderate COVID-19 symptom presentation in the real world as compared with flu, our work suggests that applications leveraging PGHD for COVID-19 detection should be validated not only in cohorts comprised of COVID-19-positive and healthy cases, but also on flu cases, as intermingling the two may significantly increase false positive rates.

RESULTS

Data Collection and Cohort Definitions

We compare a cohort of self-reported diagnosed COVID-19 cases (n = 230) to two groups of diagnosed flu cases: non-COVID-19 flu cases (n = 426), which occurred in the same time frame as the COVID-19 cases, and pre-COVID-19 flu (n = 6,270), which occurred earlier in the 2019–2020 flu season before the outbreak of COVID-19. All cases were identified through curation of a data set of 194,401 responses to a longitudinal survey about ILI symptoms (Figure 1). The rationale of splitting flu comparators into two separate groups is to be able to account for behavioral and physiological confounding factors brought about by lockdown and other measures. In addition, these three primary cohorts were filtered to those participants in each cohort with wearable sensor data and a low fraction of missing data (dense data), focusing on Fitbit wearable sensors (all models). In the COVID-19 cohort, 33 have dense RHR, 35 have dense sleep data, 36 have dense step data, and 41 participants have dense Fitbit data in any of the data channels. In the non-COVID-19 flu cohort, 85 have any dense Fitbit data (60 RHR, 64 sleep, 80 steps), and in the pre-COVID-19 flu cohorts, 1,226 have dense data in any channel (1,025 in RHR, 979 in sleep, 1,193 in steps; Figure 2). The lower counts for RHR stem from the fact that some Fitbit models do not support RHR, while the lower counts for sleep are due to the fact that some participants do not wear the sensor while sleeping. The three primary cohorts, filtered to account for sensor data availability and density are used to estimate changes in wearable data in the neighborhood of self-reported symptoms.

Survey Results

Demographic Differences between COVID-19 and Flu Cases

A demographic summary of the three cohorts is provided in Table 1. A chi-square test of independence was performed for each demographic category to test for significant differences across cohorts. Age group and race differed significantly among the cohorts after applying a Bonferroni correction to adjust for performing five comparisons (age group, $p = 0.008$; race, $p < 0.001$), while gender, education, and body mass index (BMI) did not differ significantly. Compared with the pre-COVID-19 flu cohort, the COVID-19 cohort was less likely to be white/caucasian (63.9% versus 70.0%, follow-up two-proportion z test, $p = 0.047$) and more likely to be Asian or Pacific Islander (9.6% versus 4.6%, $p < 0.001$). A greater proportion of the

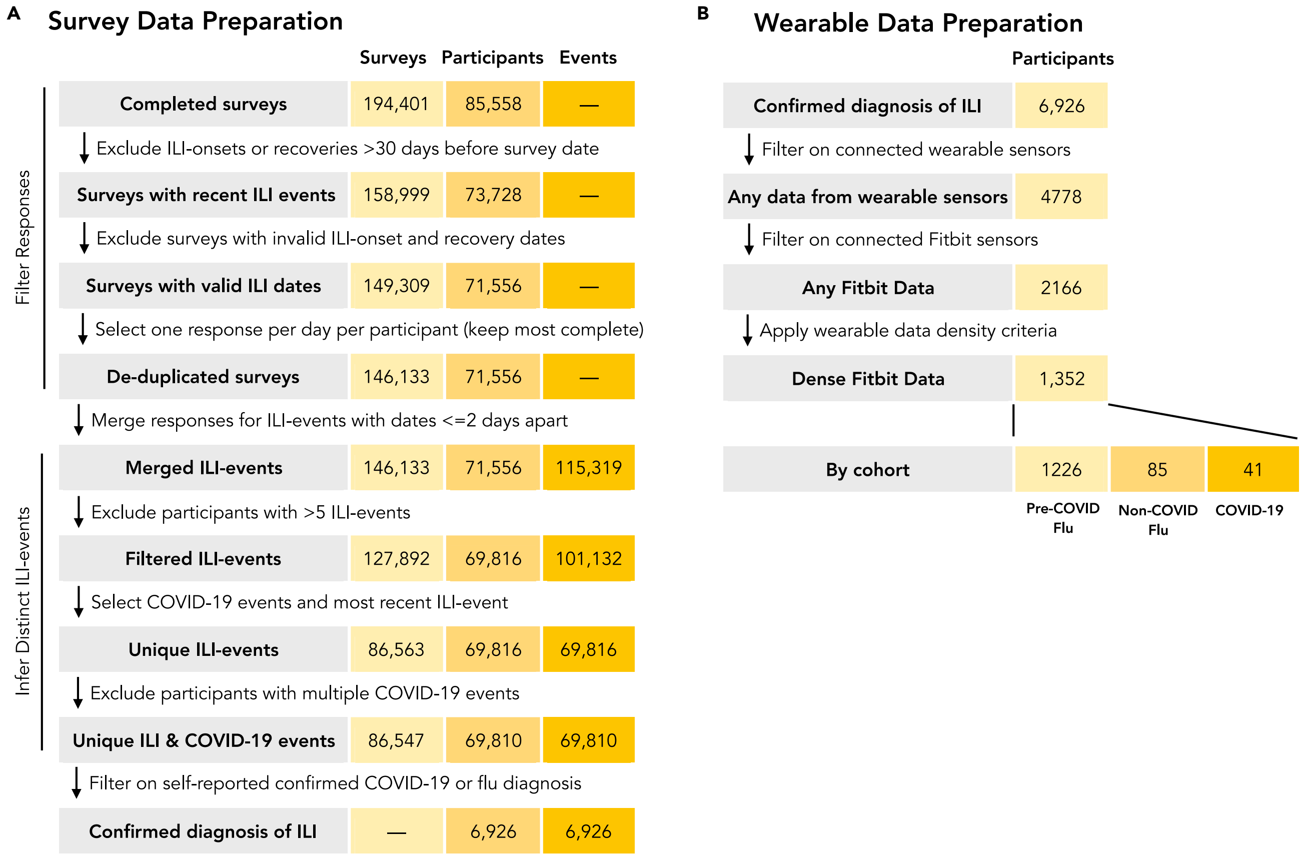

Figure 1. Data Preparation Schema
(A) Flow diagram for preparing survey data for analysis. Data preparation consisted of filtering survey responses and merging responses that correspond to the same ILI event.
(B) Flow diagram for preparing Fitbit wearable data. Participants with insufficiently dense data were filtered out.

non-COVID-19 flu cohort was aged 55 years or older compared with the pre-COVID-19 flu cohort (7.5% versus 3.8%, two-proportion z test, $p = 0.001$). The demographics of the analyzed cohorts tended to be younger and there were more females compared with those described in the literature for medically attended ILI events for the general US population,[27] and should be reweighed[28] before meaningful comparisons can be made with a target population with different demographic characteristics. The cohorts in this study are based on convenience sampling and are not representative of the US population.

Healthcare Interactions Differ between COVID-19 and Flu Cases

Although all patients had to report seeking medical care and being diagnosed by a medical provider with either flu or COVID-19 to be included the analyses, locations of medical care, hospitalization rates, and medication prescription rates differed significantly across the three cohorts (chi-square tests of independence with a Bonferroni correction, all $p < 0.001$; summarized in Table 2). Compared with non-COVID-19 flu and pre-COVID-19 flu patients, COVID-19 patients were less likely to seek care at a primary care clinic (37.4% versus 50.2% for non-COVID-19 flu, $p = 0.002$, versus 45.7% for pre-COVID-19 flu, $p < 0.001$) or urgent care facility (16.1% versus 23.5% for non-COVID-19 flu, $p = 0.026$, versus 39.1% for pre-COVID-19 flu, $p < 0.001$) and more likely to seek care in an emergency room (17.0% versus 8.2% for non-COVID-19 flu, $p < 0.001$, versus 6.9% for pre-COVID-19 flu, $p < 0.001$) or other location (37.4% versus 50.2% for non-COVID-19 flu, $p = 0.002$, versus 45.7% for pre-COVID-19 flu, $p < 0.001$). Informal review of the text responses provided for "other" locations indicated that COVID-19 patients were more likely to seek care via telehealth services.

COVID-19 patients were more likely to be hospitalized (36.1%) than non-COVID-19 flu (15.7%, $p < 0.001$) and pre-COVID-19 flu (7.1%, $p < 0.001$) patients. Interestingly, a greater proportion of patients with recent flu events were hospitalized than those with flu events earlier in the season (15.7% non-COVID-19 flu versus 7.1% pre-COVID-19 flu, $p < 0.001$). This result may relate to the fact that a higher proportion of individuals in the non-COVID-19 cohort were older than 55 years. In addition, provider behavior during the initial stages of the COVID-19 pandemic when rapid diagnostic testing was not widely available may have prompted medical providers to admit patients seeking care with ILI symptoms to hospitals at a higher rate than would have otherwise been observed.

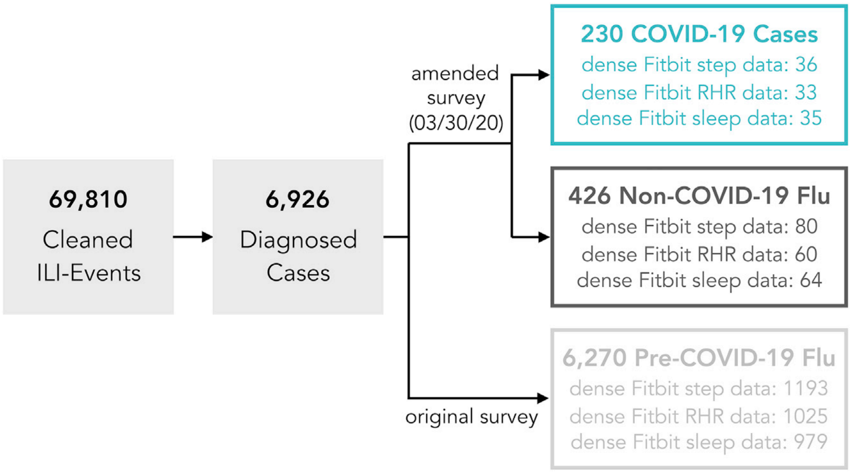

Figure 2. Definition of Analysis Cohorts

All participants included in the analyses reported that they sought medical care and were diagnosed with either flu or COVID-19 by a healthcare provider. Participants who indicated they were diagnosed with both flu and COVID-19 (n = 83) were assigned to the non-COVID-19 flu cohort.

The COVID-19 and non-COVID-19 flu cohorts were less likely to be prescribed medication (baloxavir marboxil, oseltamivir, zanamivir, antibiotics, and/or other) than the pre-COVID-19 flu cohort ($p < 0.001$ and $p < 0.001$), but the medication rates between COVID-19 and non-COVID-19 flu patients did not differ significantly ($p = 0.202$).

Differing Presentation of COVID-19 and Flu Symptoms

A summary of self-reported symptom prevalences for the COVID-19 and flu cohorts is reported in Table 3. The most common symptoms across all groups included cough, headache, body muscle ache, fatigue, and fever. Symptoms prevalence was significantly different across the three cohorts (chi-square test of independence, $p < 0.001$). All follow-up pairwise symptom comparisons were tested with two-proportion z tests and a Bonferroni correction was applied for performing 33 tests.

Compared with the non-COVID-19 flu cohort, patients with COVID-19 were significantly more likely to report experiencing cough (84.3% versus 71.6%, $p < 0.001$), loss of sense of smell (anosmia; 38.3% versus 15.5%, $p < 0.001$), persistent pain or pressure in the chest (49.6% versus 19.7%, $p < 0.001$), and shortness of breath or difficulty breathing (65.7% versus 24.2%, $p < 0.001$). These are generally accepted as the canonical symptoms of COVID-19.[29] Although it is important to note that, with the exception of cough, while these symptoms have moderate positive predictive value (higher relative prevalence in COVID-19 cases as compared with flu), they are still relatively insensitive markers of COVID-19 (low absolute prevalence in COVID-19 cases).

Compared with the pre-COVID-19 flu cohort, the COVID-19 cohort was significantly less likely to report experiencing body muscle ache, fever or feeling feverish, nasal congestion or runny nose, sneezing, chills or shivering, and sweats (all $p < 0.001$). Several symptoms (i.e., shortness of breath, anosmia, and chest pain) could not be compared between the COVID-19 and pre-COVID-19 flu cohorts because they were not included in the original survey.

With the exception of headache, all symptoms were significantly less prevalent in the non-COVID-19 flu cohort relative to the pre-COVID-19 flu cohort. One possible reason for the difference in symptom presentations in the two flu cohorts is that the 2019–2020 flu season consisted of two waves of different flu strains: strain B (Victoria lineage) appeared earlier on and was followed by strain A (H1N1-pdm09).[30] Vaccines for the 2019–2020 season were well-matched against circulating strain A but not as well-matched against strain B,[31] which could account for more mild symptom presentation in the recent flu cases in the non-COVID-19 flu cohort compared with cases in the pre-COVID-19 flu cohort.

We also examined the prevalence of co-occurring sets of symptoms for the COVID-19 and non-COVID-19 flu cohorts (Figure 3). The pre-COVID-19 flu cohort was excluded from this analysis for two reasons. First, comparing the COVID-19 and non-COVID-19 flu cohorts provides two cohorts that are comparable contemporaneously. Second, due to the fact that the pre-COVID-19 flu cohort utilized a survey requesting only a subset of symptoms, adding this cohort was of limited utility in assessing symptom constellations. For simplicity of illustration, we unioned only the five most prevalent symptom sets in each cohort, which resulted in a subset of seven individual symptoms: cough, headache, fever, fatigue, body muscle ache, chills or shivering, and shortness of breath. The two most common symptom sets consisted of all symptoms, which was predominated by COVID-19 cases, and all symptoms except for shortness of breath, which was predominated by non-COVID-19 flu cases. The symptom pair of shortness of breath and cough, and the set of all symptoms other than chills or shivering were also more indicative of COVID-19 than non-COVID-19 flu.

COVID-19 Symptoms Tend to Peak Later and Last Longer than Flu

The duration of each ILI event (illness period) in days was calculated from self-reported dates of illness onset and illness recovery (Figure 4). COVID-19 illnesses tended to last longer than flu illnesses, lasting a median of 12 days, compared with a median of 9 days for non-COVID-19 flu illnesses and 7 days for the pre-COVID-19 flu illnesses. Compared with the non-COVID-19 and pre-COVID-19 flu cohorts, most of the COVID-19 cohort experienced a longer duration of illness (Mood's median test, $p = 0.003$ and $p < 0.001$, respectively). The observed slightly longer duration of non-COVID-19 flu as compared with pre-COVID-19 flu, despite the overall milder symptoms of non-COVID-19 flu, may depend on potential contamination of the non-COVID-19 flu with COVID-19 cases, which have a longer duration on average, with symptoms mild enough and non-specific enough to not warrant a test, and thus get reported as flu.

Symptom prevalence across each cohort for each day after the date of self-reported illness onset is illustrated in Figure 5, and the days of peak symptom occurrence for each cohort are reported in Table 3. The peak days of symptom occurrence were significantly

Table 1. Demographic Summaries for the Full COVID-19 (COVID), Non-COVID-19 Flu (NCF), and Pre-COVID-19 Flu (PCF) Cohorts, as well as the Subset of Each Cohort with Dense Steps, RHR, and/or Sleep Data

	Full Cohorts			Sub-cohorts with Dense Sensor Data		
	COVID (n = 230), %	NCF (n = 426), %	PCF (n = 6,270), %	COVID (n = 41), %	NCF (n = 85), %	PCF (n = 1,226), %
Gender						
Female	70.0	74.4	78.2	80.5	76.5	82.8
Male	28.7	24.6	20.8	17.1	21.2	16.4
Other	0.4	0.7	0.3	0.0	1.2	0.2
Unavailable	0.9	0.2	0.6	2.4	1.2	0.7
Race and ethnicity						
White/caucasian	63.9	66.4	70.0	56.1	75.3	74.8
Hispanic or Latino	7.0	6.8	8.3	9.8	2.4	5.2
Black or African American	3.5	7.0	6.0	7.3	5.9	3.2
Asian or Pacific Islander	9.6	8.0	4.6	9.8	7.1	3.1
American Indian or Alaskan Native	1.3	0.7	0.8	0.0	1.2	0.6
Prefer not to answer	4.8	1.6	1.4	7.3	0.0	1.1
Unavailable	10.0	9.4	8.8	9.8	8.2	12.1
Education						
Did not finish high school	2.2	1.4	1.8	0.0	0.0	1.1
High school diploma or GED	8.7	11.5	12.2	7.3	15.3	9.9
Some college, no degree	21.3	24.2	23.0	19.5	20.0	22.0
Trade/technical/vocational training	4.3	5.4	5.3	7.3	7.1	5.6
College degree	34.8	30.3	36.9	29.3	31.8	39.0
Graduate degree	17.4	18.3	14.3	22.0	20.0	15.8
Doctorate degree or MD	3.0	3.1	1.5	2.4	0.0	1.6
Prefer not to answer	0.0	0.2	0.1	0.0	1.2	0.1
Unavailable	8.3	5.6	4.7	12.2	4.7	4.9
Age (years)						
<25	20.9	16.4	17.9	14.6	11.8	9.2
25–34	40.0	37.3	39.7	41.5	37.6	38.4
35–44	22.6	23.9	25.8	26.8	21.2	28.2
45–54	10.4	14.8	12.2	9.8	16.5	17.0
55+	5.2	7.5	3.8	4.9	12.9	6.7
Unavailable	0.9	0.0	0.5	2.4	0.0	0.4
BMI						
<18.5	3.5	4.2	2.6	2.4	4.7	2.4
18.5–24.9	27.0	21.8	24.0	22.0	21.2	21.9
25.0–29.9	24.3	22.3	24.3	22.0	28.2	24.6
30+	32.2	37.6	38.4	43.9	34.1	42.1
Unavailable	13.0	14.1	10.8	9.8	11.8	9.0

Note that table percentages may not add up to exactly 100% due to rounding applied during formatting.

different across the three cohorts for all symptoms (Mood's median test: $p < 0.001$). The peak days were significantly different for all symptoms when comparing non-COVID-19 flu and pre-COVID-19 flu cohorts as well as when comparing COVID-19 and pre-COVID-19 flu cohorts. Compared with the non-COVID flu cohort, the COVID-19 cohort had significantly different peak symptom days for fever ($p < 0.001$), cough ($p = 0.014$), nasal congestion ($p < 0.001$), fatigue ($p < 0.001$), sweats ($p = 0.011$), chest pain ($p = 0.007$), shortness of breath ($p = 0.006$), and anosmia ($p = 0.007$), but not for other symptoms. In general, day-by-day symptom prevalence peaked later for the COVID-19 cases compared with the two groups of flu cases. With the exception of shortness of breath for the non-COVID-19 flu cohort, all symptoms peaked 2–3 days after illness onset in both flu cohorts. In contrast, COVID-19 symptoms peaked 3–7 days after illness onset, with most symptoms peaking 4–5 days after illness onset. Some of the latest peaking symptoms are those that are most tightly associated with COVID-19, including fever, cough, nasal

Table 2. Summaries of Medical Care-Seeking Behaviors and Outcomes for the COVID-19 (COVID), Non-COVID-19 Flu (NCF), and Pre-COVID-19 Flu (PCF) Groups

	COVID (n = 230), %	NCF(n = 426), %	PCF (n = 6,270), %
Medical care location			
Primary care clinic	37.4	50.2	45.7
Urgent care facility	16.1	23.5	39.1
Emergency room	17.0	8.2	6.9
Ear, nose, and throat clinic	2.2	2.1	0.8
Infectious disease clinic	1.7	1.2	0.4
Other	10.9	4.7	4.3
Multiple locations	14.8	10.1	2.8
Hospitalized			
Yes	36.1	15.7	7.1
No	63.9	83.6	92.6
Unavailable	0.0	0.7	0.3
Prescribed medication			
Yes	62.2	67.1	79.4
No	37.0	30.8	19.2
Do not know/remember	0.9	1.4	1.1
Unavailable	0.0	0.7	0.3

Overall statistics are shown, including where medical care was sought, whether patients were hospitalized, and whether they were prescribed medication.

congestion, fatigue, shortness of breath, chest pain or pressure, and anosmia (Figures 5F–5M, respectively).[29]

Wearable Data Results

Demographics of Participants with Dense Wearable Sensor PGHD

Data from commercial Fitbit sensors were available for at least 1 day between 2019-11-01 and 2020-05-13 for approximately 31% of all participants. A smaller subset of these participants met the criteria for sensor data density (see Experimental Procedures) and were included in the analysis, including 41 (18%) COVID-19 patients, 85 (20%) non-COVID-19 flu patients, and 1,226 (20%) pre-COVID-19 flu patients. The demographics of the cohorts with dense sensor data are described in Table 1. We tested for demographic differences among participants with and without dense sensor data using the same two-step statistical testing procedure described previously. Pooling across the three cohorts, compared with participants without dense Fitbit data (n = 5,574), those with dense Fitbit data (n = 1,352) were more likely to be female ($p < 0.001$), white ($p < 0.001$), obese (30+ BMI, $p = 0.003$), and in an older age group (45–54, $p < 0.001$; 55+, $p < 0.001$).

COVID-19 Illness Onset Was Associated with Elevated RHR

Given the known association between elevated RHR and the inflammatory immune system response,[9,10] we examined the prevalence of elevated RHR around ILI events. RHR is computed by commercial Fitbit sensors. While the exact algorithm estimating RHR is proprietary to Fitbit,[32] it approximately coincides with heart rate observed during periods of deep sleep or inactivity. Following a previously described methodology,[8] we define an RHR measurement as elevated for a participant if they are 0 (minimally elevated), 0.5 (moderately elevated), or 1 (highly elevated) standard deviation(s) above mean RHR, where both mean and standard deviation are computed for each participant over the entire 189-day-long observation window, consisting of both the baseline and ILI event windows. Figure 6 illustrates the fraction of each cohort with elevated RHR, for different elevation thresholds, for each day relative to symptom onset for each of the three cohorts. In both the COVID-19 and pre-COVID-19 flu cohorts, the prevalence of highly elevated RHR was highest in the first days after illness onset. In particular, the percent of COVID-19 patients with highly elevated RHR was higher around the onset of COVID-19 from days −2 to 2 (25%), when infectivity is at its peak and isolation interventions could have maximum effectiveness,[16] as compared with days −10 to −5 (13%, two-proportion z test $p = 0.005$) when transmissibility is less likely.[16] Remarkably, the percentage of the COVID-19 cohort with highly

Table 3. Summary of Self-reported Symptoms for the COVID-19 (COVID), Non-COVID-19 Flu (NCF), and Pre-COVID-19 Flu (PCF) Cohorts

	Symptom Prevalence			Peak Symptom Day Relative to Illness Onset		
	COVID (n = 230), %	NCF (n = 426), %	PCF (n = 6,270), %	COVID	NCF	PCF
Cough	84.3	71.6	85.1	5	3	3
Headache	71.3	68.1	74.3	4	3	3
Body muscle ache	66.1	67.1	80.8	4	2	2
Shortness of breath	65.7	24.2	N/A	6	8	N/A
Fatigue	61.7	54.7	70.9	5	3	3
Fever	61.3	62.0	74.6	5	2	2
Chills or shivering	53.5	55.4	69.3	4	3	2
Sore throat	51.7	48.8	61.1	3	2	3
Nasal congestion	49.6	49.3	65.4	5	2	3
Chest pain/ pressure	49.6	19.7	N/A	6	2	N/A
Sweats	42.2	47.4	56.3	5	2	2
Anosmia	38.3	15.5	N/A	7	3	N/A
Sneezing	37.0	36.9	48.2	4	2	3

Symptom prevalence refers to the percentage of the cohort reporting the symptom at any time during the ILI event and symptoms are sorted by most (top) to least (bottom) prevalence in the COVID-19 cohort. The day of peak symptom occurrence relative to illness onset corresponds to the maximum of a centered 5-day rolling mean of day-by-day symptom prevalence for each cohort. Some symptoms (i.e., shortness of breath, chest pain/pressure, and anosmia) were only included in the updated survey and therefore are not available for the pre-COVID-19 flu cohort. N/A, not applicable.

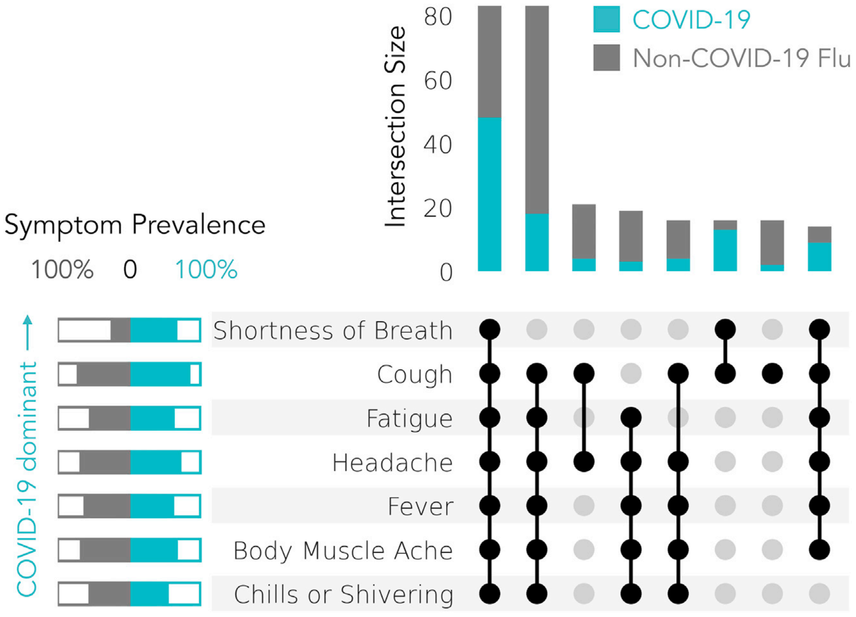

Figure 3. Co-occurrence of Self-Reported Symptoms in COVID-19 Cases or Non-COVID-19 Flu Cases

Only the top 5 most prevalent symptoms in each cohort are included in the symptom sets and only symptom sets that represent 2% or more of total COVID-19 (n = 230, blue) and non-COVID-19 flu cases (n = 426, gray) are plotted. Symptoms are sorted by their relative prevalence in COVID-19 (top) versus non-COVID-19 flu (bottom) cases.

elevated RHR around illness onset was greater than that of the non-COVID-19 cohort (16%, p = 0.026), but did not differ from that of the pre-COVID-19 cohort (22%, p = 0.454), which suggests that RHR elevation alone may not be a specific marker of infection. Non-COVID-19 flu appears to display a noticeable peak only for the moderately elevated RHR, which corroborates the hypothesis of milder symptom severity of non-COVID-19 flu as compared with pre-COVID-19 flu.

Activity Decrease during COVID-19 and Flu Illnesses

To quantify the impact of COVID-19 and flu on objective, sensor-based measures of behavior, we examined the extent to which daily steps and sleep deviated from expected measurements before and after illness onset. To this end, we first estimated for each participant what their expected measurement (total sleep duration, step counts) for a given day would be during illness *had they not been sick*, based on models fit on a pre- and post-illness baseline period (see Experimental Procedures for details). Subsequently, we computed excess activity during the ILI event as the difference between the unobserved estimated "healthy day" measurement and the observed one during a sick day. Generalized additive models were fit to the excess values separately for each cohort (mgcv package for R) and the resulting regression splines were used for visualizations. Figure 7 illustrates the impact of illness in terms of excess daily steps lost and additional minutes of sleep. Reductions in daily step counts were more marked for pre-COVID-19 flu than they were for non-COVID-19 flu, supporting the hypothesis of milder symptoms of non-COVID-19 flu, and the fact that mobility-reducing shelter-in-place measures during non-COVID-19 flu limit the maximum amount of lost step counts. Reduction in daily steps were also more marked and prolonged for COVID-19 patients as compared with non-COVID-19 flu and pre-COVID-19 flu patients. This may be explained by the adoption of more stringent self-imposed quarantine measures after a COVID-19 diagnosis (89% of the COVID-19 cohort reported being told by a medical provider to self-quarantine compared with only 57% of the non-COVID-19 flu cohort), but given the fact that excess is computed taking into consideration reduced mobility caused by shelter-in-place measures, the observed reduction could also be a reflection of the more prolonged illness durations in the COVID-19 patients as captured by self-reported symptoms and discussed in previous sections. In addition, the step reduction appears to persist beyond the 2-week default quarantine mandate period, and if confirmed on longer time horizons it could be an indication of COVID-19 patients experiencing "long COVID"[33]: a phenomenon in which symptoms persist for many weeks or months after the illness is meant to subside.

Sleep changes are largely inconclusive, as the post-onset total sleep time increase observed for pre-COVID-19 flu may be explained by changes in sleeping schedules during sick days that would be less prominent for the COVID-19 and non-COVID-19 flu cases, which are concurrent with widespread shelter-in-place measures. When directly comparing the non-COVID-19 flu and COVID-19 cohorts, post-onset sleep excess seems to persist for prolonged periods, hinting once again to a longer duration of symptoms and disruption of behavior brought about by a COVID-19 infection.

DISCUSSION

We present a report on PGHD, including longitudinal symptoms reports and linked physiologic and behavior data from commercial wearables collected remotely in real-life settings for 6,696 diagnosed flu and 230 diagnosed COVID-19 patients.

Chest pain, shortness of breath, and anosmia, as well as combinations of these symptoms (e.g., shortness of breath and coughing) were more prevalent in COVID-19 as compared with non-COVID-19 flu. Other symptoms, including fatigue and cough, were more prevalent later after illness onset for COVID-19 cases relative to flu cases. Similarly, patients reported longer illness duration for COVID-19 (median of 12 days) as compared with non-COVID-19 and pre-COVID-19 flu illnesses (9 and 7 days, respectively).

Differences in self-reported symptoms are supported by data from wearable sensors. We observed larger, more prolonged reductions in daily step counts for COVID-19 patients as compared with other groups. This is consistent with the observed longer illness durations for COVID-19 generally, sometimes lasting weeks or months in what is now being referred to as "long COVID."[33] Wearable sensors could be particularly useful in

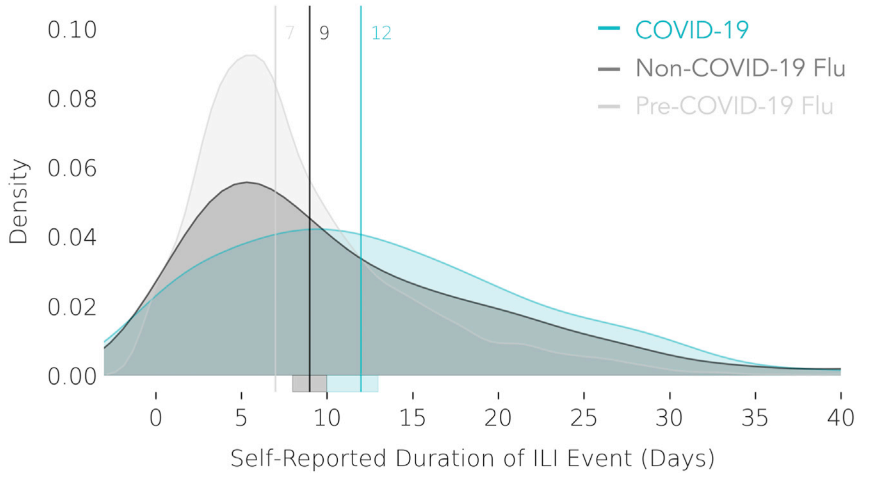

Figure 4. Self-Reported Illness Duration in Days for COVID-19, Non-COVID-19 Flu, and Pre-COVID-19 Flu Cases

Vertical lines denote the median illness duration. COVID-19 (n = 230, blue); non-COVID-19 flu (n = 426, gray); pre-COVID-19 flu (n = 6,270, light gray). Error bands around foot of vertical median lines represent bootstrapped 95% confidence intervals.

monitoring recovery from long COVID, as their unobtrusive nature may guarantee steady data collection for longer time horizons for which daily self-reporting of symptoms may become too burdensome for the participant.[34]

We observed a significantly increased fraction of participants with elevated RHR measurements in the 2 days surrounding ILI symptom onset. This has previously been observed for other ILIs[7] and is now also observed for COVID-19 patients. Several recent works have explored use of wearable data, including RHR, to detect symptoms of COVID before they appear toward applications that can prompt users to intervene in pre-symptomatic disease phases and curb the spread of infection (e.g., self-quarantine while waiting for a confirmatory test).[22–24,35] While these systems have shown moderate discriminative ability between COVID-19 patients versus healthy persons in retrospective cohorts, our findings suggest that the specificity of those systems should also be measured as compared with flu patients, as they will be the overwhelming majority as flu season starts. If specificity versus flu and other respiratory viruses cannot be demonstrated, early-warning systems triggered on wearable data should be considered as more non-specific "infection screening," and therefore be coupled with appropriate confirmatory testing mechanisms that can help to quickly relieve self-imposed quarantine of non-COVID-19 infections.[17,18]

From a methodological perspective, we note that we found it helpful to be able to compare two different flu comparator arms, one of which was contemporaneous with COVID-19, as it allows partial control for the society-scale shifts in behavior that the pandemic has brought about. For example, we noted that medication prescription rates were lower for the COVID-19 and non-COVID flu cohorts as compared with the pre-COVID flu cohorts. This may suggest that, during the pandemic, there may be additional barriers to treatment that have arisen due to lockdown measures and changes in attitudes toward risks in seeking care. Without two flu comparator arms, including one that is pre-pandemic, it would have been impossible to disentangle reduction of prescriptions coming from lockdown versus reduction coming from fewer available treatments for the novel COVID-19. While we do not have additional data to investigate these hypotheses, they may be worthwhile directions for further research.

Biases and Limitations

The studied cohorts come from convenience samples that are not representative of the US population at large. In particular, we note that African Americans and males, alongside older individuals, are underrepresented in our cohort, thus limiting the generalizability of our findings. Increasing access and usage of these tools in these risk groups is of critical importance.[36]

Differences in the rate of the COVID-19 occurrence across demographic groups and disease severity levels have received attention recently,[37–42] with increasing evidence that some racial and ethnic minority groups are being disproportionately affected by COVID-19, and preliminary findings point to possible differences with other ILIs as well.[43–45] Large-scale connected populations could be a key tool in examining the impact COVID-19 is having across demographic and geographic groups, helping to highlight vulnerable populations and target care delivery.[4] However, the cohort of individuals utilizing wearable data in this study may not reflect the heterogeneity of the general populace and, as such, results relying on wearable data may not generalize to all new populations.

Hospitalization rates for the COVID-19 and non-COVID-19 flu participants in this study were higher than national estimates from the same time frame,[46–48] which could be due to the fact that participants were required to report that they both sought medical attention *at a clinic or urgent care facility*, and were diagnosed by a medical provider to be included in the analysis. Due to shortages in testing availability and stringent testing criteria at the early stage of the pandemic and at the time of these surveys, our cohorts of diagnosed individuals may be composed of people who had more severe symptoms and thus were more likely to seek medical care, those with greater access to healthcare resources, or those who were already hospitalized when tested. Higher hospitalization rates in the post-pandemic cohorts may also be attributed to elevated monitoring and quarantining efforts in an attempt to control spreading. In addition, given that only individuals who sought medical care were included in the analysis, this may skew the symptom presentation toward more severe symptoms, such as shortness of breath or difficulty breathing, as opposed to less worrisome symptoms (e.g., sneezing). In parallel, the medication prescription rate is likely to vary by patient symptom presentation and severity upon healthcare visit and were not explored further by medication type or adherence in this study.

Another limitation comes from the fact that the analysis considers self-reported symptoms and self-reported diagnostic test confirmation. During the early period of the pandemic,

CellPress
OPEN ACCESS

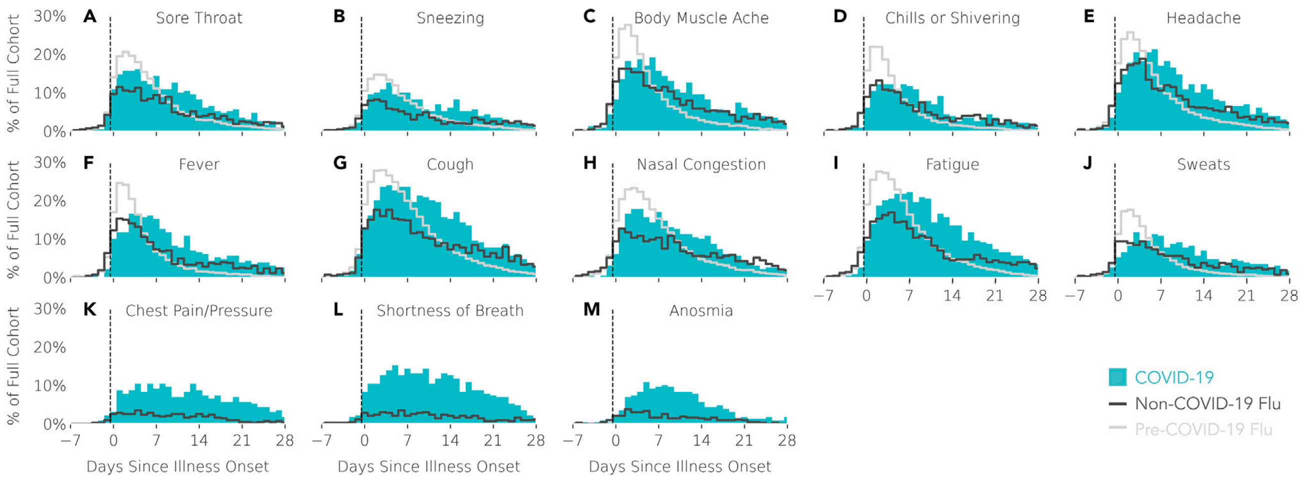

Figure 5. Self-Reported Symptom Prevalence over Time Relative to Illness Onset (Day 0; Also Self-Reported)

Figure360▷ For a Figure360 author presentation of this figure, see https://doi.org/10.1016/j.patter.2020.100188.

Prevalence is reported as a percentage of the full cohort of COVID-19 cases (n = 230; blue), non-COVID-19 flu cases (n = 426; dark gray), or pre-COVID-19 flu cases (n = 6,270; light gray trace). (A–M) Each subplot contains data for one symptom and symptoms are sorted by peak symptom occurrence (earliest to latest) for the COVID-19 cases. Note that negative values of "Days since illness onset" reflect the number of days preceding the self-reported illness onset.

diagnostic tests for COVID-19 suffered from highly heterogeneous administration policies and inaccuracies, which may have biased cohort composition. Finally, the surveys used to capture symptoms between the earlier pre-COVID flu cohort and the COVID-19 and non-COVID flu cohorts used slightly different symptom sets, making direct comparison of symptom prevalence infeasible in some cases.

Outlook

Using PGHD from self-reported symptoms, in combination with physiological and behavioral measures continuously and unobtrusively tracked by commercial wearable sensors, allows us to confirm and contextualize learnings that may be otherwise lost when considering each data stream in isolation. As more PGHD is collected and evidence is created, it is important to keep in mind that inclusion of additional data streams may increase utility, but does not immediately yield increased representativeness, especially when new streams continue to be digitally mediated and are thus biased toward access to digital technologies.[49] Making sure that PGHD generation is not restricted to a niche of the population is perhaps the current biggest limitation, and the most important agenda item on which we need to continue to make progress.

In the specific context of COVID-19, our findings support the case made by recent work that data from wearable sensors may provide low-sensitivity testing capability with daily frequency.[22–24,35] Low-sensitivity/high-frequency testing when combined with a low-delay confirmatory testing strategy has been shown by computation models to significantly reduce prevalence of spreading with minimal burden on pre-emptive quarantine for false positives.[14,16] Therefore, wearables could potentially support use cases, such as return to work and college reopening,[18] where most of the cohort can be asked to wear the sensors frequently.[17] To better understand feasibility, however, further research is needed. First, it is important to accurately quantify the sensitivity of wearable-based alert systems in prospective validation, and especially for asymptomatic/pre-symptomatic patients (who collectively seem to be responsible for more than 40% of the total infections).[50] To this end, an understanding of the ability of PGHD to detect pre-symptomatic and asymptomatic spreading cannot be derived from data based solely on symptoms. Therefore, studies designed to combine PGHD with direct measures of infectivity (e.g., PCR tests) constitute a necessary next step to understand sensitivity to asymptomatic/pre-symptomatic infections, and they are currently under development.[51]

Second, as highlighted by the current work, it is important to understand the specificity of any PGHD-based early-warning system as compared with other respiratory diseases, such as the seasonal flu, that may have similar physiological and behavioral fingerprint in addition to a large symptom overlap. We encourage researchers presenting results of COVID-19 early-warning systems based on PGHD in real-world settings to contextualize their findings, taking the confounding effect of flu into account, instead of assuming "non-COVID-19" to be a synonym of healthy controls. Despite the expectation of reduced impact due to lockdown measures, flu is still seen as a confounding factor for population-level estimates of the burden of respiratory illness when COVID-19 and flu coexist.[52] Analogously, flu must be taken into serious consideration as a confounding factor for any PGHD-based applications, being that at population or individual level. An accurate understanding of detection parameters (sensitivity, specificity, lag) in true real-world settings is crucial not only to understand feasibility, but also to understand how to shape the interaction between the system and its users. For example, we must consider how to incorporate confirmatory tests that test on multiple pathogens, not only COVID-19. The usability, including perceptions around effectiveness, burden, and privacy, will ultimately define adoption of such systems at scale.[49,53]

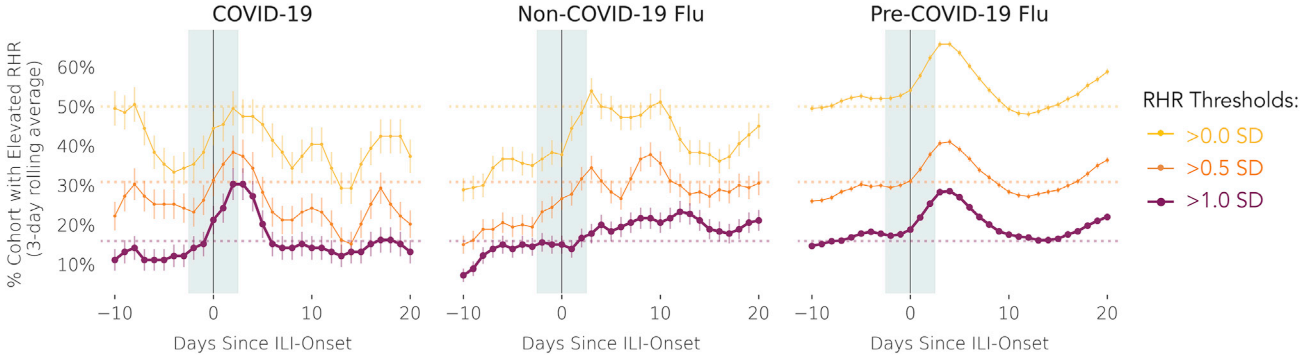

Figure 6. Fraction of Participants with Elevated Daily RHR on Days Surrounding Illness Onset (Day 0)
Elevated RHR is defined as being greater than 1, 0.5, or 0 standard deviations (SD) over all daily RHRs observed during the combined baseline and symptomatic periods. Cohorts refer to the sub-cohorts with dense RHR data: COVID-19 (n = 33; left), non-COVID-19 flu (n = 60; middle), and pre-COVID-19 flu (n = 1,025; right). Error bars represent the SD of the sample proportions.

As care becomes more decentralized and telehealth becomes more widespread,[54] PGHD can become a valuable tool on an individual level as patients transition in and out of care.[55] In addition to providing support to individual-level early-warning systems and population-level hotspot detections, PGHD could enable monitoring of recovery from symptoms, as the unobtrusive nature of sensor-based PGHD makes consistent monitoring possible over the weeks and months of long-COVID recovery.

The vast majority of learning about COVID-19 has come from real-world data sources, such as health records and claims.[56] PGHD can be a crucial addition, adding a large-scale understanding of early signals, several days before impact is seen at centers of care. As the COVID-19 pandemic continues to develop, and as future annual ILI waves arrive, understanding and correctly reacting to symptom presentation will be critically important. These results support not only an emerging picture that COVID-19 has a distinct presentation, but highlight the power of PGHD, digital health, and connected populations in broadly and remotely monitoring health status.

EXPERIMENTAL PROCEDURES

Resource Availability

Lead Contact

Luca Foschini, luca@evidation.com.

Materials Availability

The amended questionnaire is available in Note S2. The original questionnaire consisted of a subset of questions from the amended questionnaire; the original questionnaire included all questions except for questions 1, 8–9, 14–16, 18, 28–30, 32, 36–38.

Data and Code Availability

The completed coded curated study data can be requested by qualified researchers via the Sage Synapse platform[57] here: https://www.synapse.org/#!Synapse:syn22891469/.

Code for reproducing all analyses in the manuscript can be found here: https://github.com/evidation-datascience/COVID19_baseline_paper.

Data Collection

Achievement is a mobile consumer application that rewards and enables members to participate in research by completing questionnaires and sharing data from commercial-grade wearable sensors.[58–60] Since 2017, Achievement has been used to run a participatory ILI surveillance program, examining annual waves of influenza virus infections.[7] The 2019–2020 version of the program recruits individuals who have experienced ILI symptoms in the past 7 days to collect information on the date of illness onset and/or recovery, detailed symptoms, healthcare interactions and outcomes, medications, and household characteristics. The questionnaire was designed with inspiration from Flu Near You,[6] as well as input from public health and clinical infectious disease experts. On 2020-03-30, the questionnaire was updated to include questions that specifically address COVID-19, including questions about COVID-19 diagnosis, testing, and social distancing measures, and an expanded list of symptoms, including shortness of breath, chest pain, and anosmia. The contents of the original and updated questionnaires are included in Note S2.

Participants agreed to share survey responses and activity data from connected wearable sensors. Responses to the original and updated surveys, collected between 2019-12-02 and 2020-04-27, comprised the initial survey dataset and included a total of 194,401 responses from 85,558 unique participants. The sensor data analyzed in this project consisted of minute-by-minute step counts, RHR recordings, and sleep states from 2019-11-01 to 2020-05-13 for the subset of participants with Fitbit sensors connected to the Achievement platform.

Survey Preparation

Survey preparation methods are described in detail in Note S3, and summarized here. Survey cleaning reduced the initial dataset to 146,133 responses from 71,556 unique participants. Since participants could submit multiple survey responses for the same ILI event, distinct ILI events were inferred by merging survey responses from the same participant when the dates encompassing self-reported illness onset through recovery overlapped or were separated by no more than 2 days (chosen to account for potential misreporting of symptom onset date). After excluding participants who reported five or more distinct ILI events and participants who reported multiple distinct COVID-19 events (to remove participants with possible erroneous or fraudulent responses; removed 1,740 participants, bringing the sample to 69,816 among whom 49,397 reported one distinct ILI event throughout the flu season and 20,419 reported more than one ILI event—for whom the most recent ILI event was selected), the analysis set was reduced to a subset of 6,926 ILI events with self-reported clinical diagnoses. Note that applying the thresholding specified above 6 (2.5%) individuals were removed from the COVID-19 cohort. Survey responses were supplemented with demographic information from another survey, including gender, age, BMI, ethnicity, race, and pre-existing health conditions. This survey could have been completed at a different time than the ILI survey, and as such there may be slight discrepancies in time-variant demographic data, such as age, BMI, and health conditions.

Cohort Definition

To be included in the analysis, participants had to answer "yes" to survey question 10, indicating that they sought medical attention from a healthcare provider for their illness, and either question 12 or 14, indicating that the healthcare provider diagnosed them with either the flu or COVID-19 (see Note S2 for

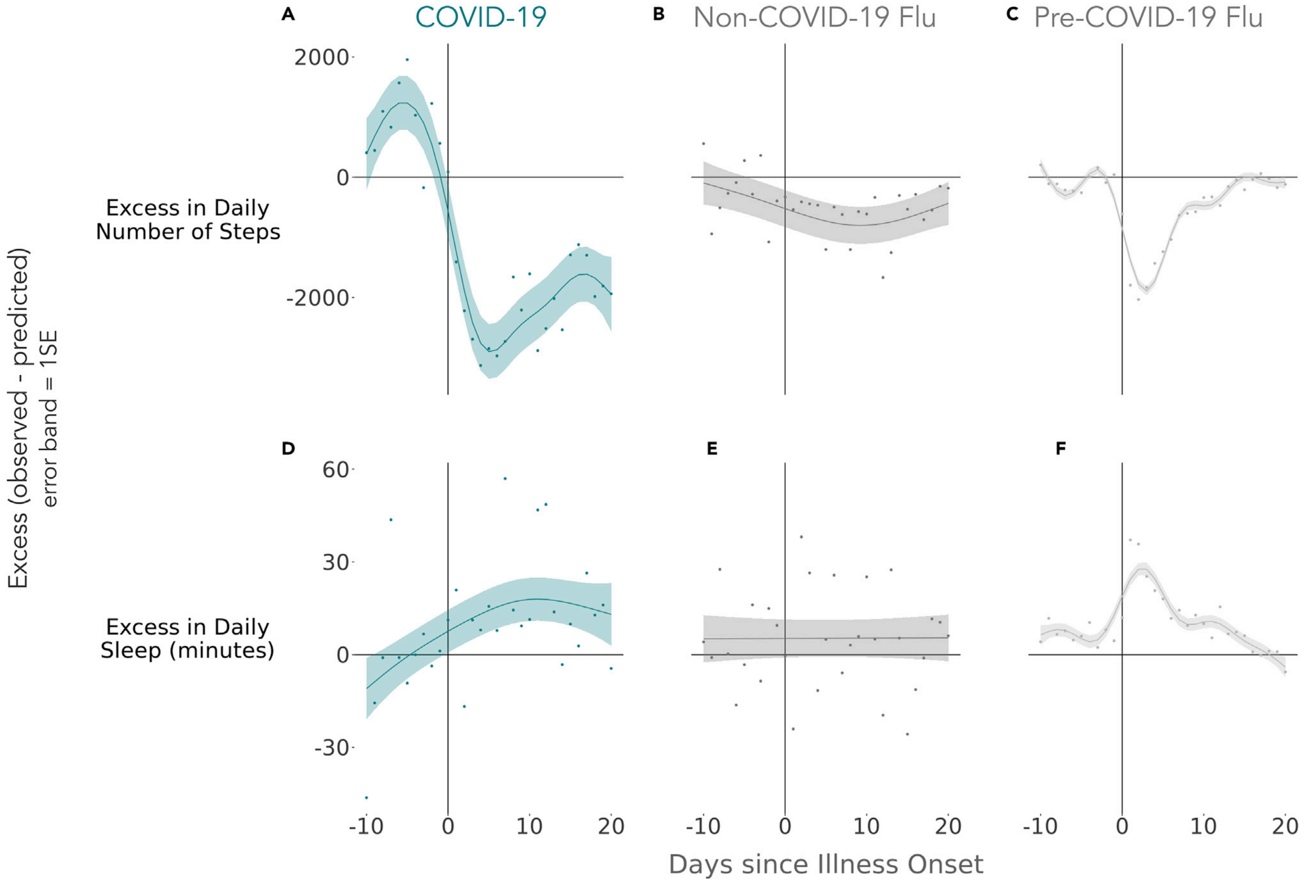

Figure 7. Deviations from Typical Healthy Behavior and Physiology Observed during ILI Events

Three measurement channels were studied: daily number of steps, daily mean RHR, and daily sleep minutes. Deviation from the norm was quantified as difference (excess) between observed values and estimates from a model fit only to symptom-free days (i.e., days outside the window of −10 through +20 days surrounding ILI onset). Greater excess indicates greater deviations from typical behavior. Sample sizes across cohorts and channels: steps analysis: (A) COVID-19, n = 36; (B) non-COVID-19, n = 80; (C) pre-COVID-19, n = 1,193; sleep analysis: (D) COVID-19, n = 35; (E) non-COVID-19, n = 64; (F) pre-COVID-19, n = 979. Error bands represent 1 SE.

question wordings). Participants who self-reported seeking medical care and being diagnosed with flu and/or COVID-19 by a healthcare provider (n = 6,926) were divided into three cohorts (Figure 2). Participants who completed the amended survey and self-reported being diagnosed with COVID-19 or flu were assigned to the COVID-19 cohort (n = 230) or non-COVID-19 flu cohort (n = 426), respectively. The non-COVID-19 flu cohort offers a comparison of COVID-19 and flu cases that is not confounded by time of year, survey content, time since survey completion, or recent large-scale societal changes, such as shelter-in-place orders or changes within the healthcare system. Participants who reported being diagnosed with flu in the original survey were assigned to the pre-COVID-19 flu cohort (n = 6,270). These cases spanned the 2019–2020 flu season before the COVID-19 outbreak and are included to provide a large comparison group of canonical flu events.

Wearable Sensor Data Preparation and Analysis

The pipeline for preparing and analyzing the sensor data is described in detail in Note S3 and summarized here. Of the 6,926 participants with diagnosed ILI events, 4,778 (69%) connected at least one wearable sensor to the Achievement platform: 2,582 (37%) participants connected Apple Watches, 2,166 (31%) connected Fitbit sensors, 420 (6%) connected Garmin sensors, 123 (2%) connected Withings sensors, and 17 (0.2%) connected Misfit sensors. We focused the analysis of sensor data on the subset of participants with connected Fitbit sensors, consisting of minute-by-minute steps, heart rate recordings, and sleep states. These data were collected from 2019-11-01 through 2020-05-13. Analyses focused on two different periods: an ILI-event period, conservatively defined as days −10 through +20 relative to self-reported symptom onset (day 0), and a baseline period—all other days before and after the ILI-event period. Given the sparse and often conflicting literature regarding the incubation period and illness duration for COVID-19 that was available at the time analysis was conducted,[61–64] the ILI-event period was intentionally wide to capture potential asymptomatic days during the incubation period of the virus (days [−10, −1]) and a potentially long recovery (days [0, 20]). Valid days were defined as those with 10 or more hours of sensor wear time or at least one main sleep period.[65] The analysis set was restricted to participants with "dense" sensor data, with at least 10% of valid days in the baseline period, and at least 50% of valid days within the ILI event. Dense sensor data were available for 41 COVID-19 patients (36 with steps, 33 with RHR, and 35 with sleep), 85 non-COVID-19 flu patients (80 with steps, 60 with RHR, and 64 with sleep), and 1,226 pre-COVID-19 flu patients (1,193 with steps, 1,025 with RHR, and 979 with sleep). For participants included in the dense wearable analysis cohort, the mean number of missing days was 10.2%, 9.5%, and 8.9% for steps, sleep, and heart rate data, respectively. Sensitivity analysis on the valid day thresholds was conducted and results did not change significantly when removing the requirement of having 10% of valid days for each day of the week, or lowering the percentage of individual valid days to as low as 30%. The pipeline for preparing the wearable data for analysis is illustrated in Figure 1B. While wear time is

estimated using minute-by-minute data, all analysis is conducted on day level variables consisting of daily RHR, daily step sum, and nightly sleep hours.

We used a mixed-effects regression model to estimate expected activity levels and adopted the model to impute gaps in RHR values for the RHR-related analysis and to provide counterfactual estimates of "typical" activity during ILI events for the excess analysis. The model specified fixed-effects for the week of the year to control for time of year effects (more specifically, this consisted of three terms for the first, second, and third expansions of an ordinal variable for week of flu season), a categorical fixed-effect for the day of the week to account for differences in activity patterns by day of week, a fixed-effect for the average daily activity level in the participants' state of residence to control for different state-wide shelter-in-place and social distancing measures, and a random-intercept for each participant's baseline activity level to control for individual differences in activity levels. Three models were specified to predict daily total steps, daily RHR, and nightly total sleep time:

$$feature = \beta_0 + \beta_1 * week + \beta_2 * week^2 + \beta_3 * week^3 + \beta_4 * day\ of\ week + \beta_5 * state\ mean + u_0 + \varepsilon.$$

We examined the fraction of each cohort with elevated RHR in the days preceding and following ILI onset.[8] First, missing RHR values were imputed by fitting the mixed-effects model described previously to all participant-days with an RHR recording. Model estimates were used for days when RHR was not recorded, and observed RHRs were used otherwise. Individualized thresholds for elevated RHR were defined as 1 standard deviation above each participant's mean RHR across all days. The fraction of each cohort with elevated RHR was computed for the days surrounding the ILI event (defined as 10 days before and 20 days after ILI onset). Two-proportion z tests were performed to determine (1) if a greater proportion of the COVID-19 cohort had elevated RHR in the days surrounding ILI onset (days −2 to 2) compared with baseline days before ILI onset (days −10 to −5) and (2) if the proportion of participants with elevated RHR surrounding ILI onset differed among cohorts. Sensitivity analysis was conducted for the elevated RHR analysis, with imputed days being entirely dropped. Results did not change materially, both with respect to the proportion of individuals with elevated RHR, and the trajectory of the elevated RHR traces.

To quantify how COVID-19 and flu impact physical activity, RHR, and sleep, we measured deviations from expected behavior during illness events. We generated individualized estimates of daily measurements that would have been recorded in the counterfactual scenario that the participant did not fall ill (that is, on a typical healthy day) by fitting the mixed-effects model to all participant-days *outside* of the ILI events (all days outside the range of −10 to 20 days from illness onset). Then we computed the *excess*, defined as the difference (observed − estimated), on the days within each ILI event. To visualize the time course of behavioral changes during COVID-19 and flu events (Figure 7), we fit generalized additive mixed models with spline smoothing functions (a thin plate regression spline for days since symptom onset with random effects for participants) to the daily excess time series for each cohort using the mgcv package for R.[66]

SUPPLEMENTAL INFORMATION

Supplemental Information can be found online at https://doi.org/10.1016/j.patter.2020.100188.

ACKNOWLEDGMENTS

This work was self-funded by Evidation Health, Inc. The authors thank Dr. Raghu Kainkaryam for technical input, Dr. Wei-Nchih Lee for input developing the survey instrument, and Christine Lemke and Stephanie Jones for feedback on the manuscript. The authors also thank Dr. Stephen Friend and the team at 4YouandMe for the invaluable partnership and discussions.

AUTHOR CONTRIBUTIONS

Conceptualization, E.R., B.B., and L.F.; Methodology, E.R., B.B., and L.F.; Investigation, E.R., B.B., and L.F.; Data Curation, N.M., A.S., E.R., I.C., J.M., and B.B.; Visualization, A.S., N.M., and B.B.; Writing – Original Draft, all authors; Writing – Review & Editing, E.R., I.C., J.M., B.B., A.S., A.T., and Y.W.; Supervision, E.R. and L.F.; Project Administration, I.C. and B.B. All authors contributed final approval of the published manuscript and are accountable for all aspects of the work, including accuracy and integrity of any part of the work.

DECLARATION OF INTERESTS

A.S., N.M., I.C., B.B., E.R., J.M., and L.F. are employees of Evidation, a company that runs research studies using person-generated health data in several therapeutic areas, including COVID-19.

All other authors declare no competing interests.

Received: July 22, 2020
Revised: November 19, 2020
Accepted: December 10, 2020
Published: December 13, 2020

REFERENCES

1. Friends of Cancer Research (2020). COVID-19 Evidence Accelerator. https://www.focr.org/covid19.
2. Massachusetts General Hospital, King's College London, and ZOE (2020). COVID symptom study—help slow the spread of COVID-19. www.covid.joinzoe.com.
3. Outbreaks Near Me (2020). Home. https://covidnearyou.org.
4. Evidation (2020). COVID-19 Pulse: Delivering regular insights on the pandemic from a 150,000+ person connected cohort. https://evidation.com/news/covid-19-pulse-first-data-evidation/.
5. Reinhart, A., and Tibshirani, R. (2020). COVID-19 Symptom Surveys through Facebook. https://delphi.cmu.edu/blog/2020/08/26/covid-19-symptom-surveys-through-facebook/.
6. Smolinski, M.S., Crawley, A.W., Baltrusaitis, K., Chunara, R., Olsen, J.M., Wójcik, O., Santillana, M., Nguyen, A., and Brownstein, J.S. (2015). Flu near you: crowdsourced symptom reporting spanning 2 influenza seasons. Am. J. Public Health *105*, 2124–2130.
7. Konty, K.J., Bradshaw, B., Ramirez, E., Lee, W.-N., Signorini, A., and Foschini, L. (2019). Influenza surveillance using wearable mobile health devices. Online J. Public Health Inform. *11*, https://doi.org/10.5210/ojphi.v11i1.9758.
8. Radin, J.M., Wineinger, N.E., Topol, E.J., and Steinhubl, S.R. (2020). Harnessing wearable device data to improve state-level real-time surveillance of influenza-like illness in the USA: a population-based study. Lancet Digital Health *2*, e85–e93.
9. Park, W.-C., Seo, I., Kim, S.-H., Lee, Y.-J., and Ahn, S.V. (2017). Association between resting heart rate and inflammatory markers (white blood cell count and high-sensitivity C-reactive protein) in healthy Korean people. Korean J. Fam. Med. *38*, 8–13.
10. Whelton, S.P., Narla, V., Blaha, M.J., Nasir, K., Blumenthal, R.S., Jenny, N.S., Al-Mallah, M.H., and Michos, E.D. (2014). Association between resting heart rate and inflammatory biomarkers (high-sensitivity C-reactive protein, interleukin-6, and fibrinogen) (from the Multi-Ethnic Study of Atherosclerosis). Am. J. Cardiol. *113*, 644–649.
11. Liautaud, P., Huybers, P., and Santillana, M. (2020). Fever and mobility data indicate social distancing has reduced incidence of communicable disease in the United States. arXiv http://arxiv.org/abs/2004.09911.
12. Kogan, N.E., Clemente, L., Liautaud, P., Kaashoek, J., Link, N.B., Nguyen, A.T., Lu, F.S., Huybers, P., Resch, B., Havas, C., et al. (2020). An early warning approach to monitor COVID-19 activity with multiple digital traces in near real-time. arXiv https://arxiv.org/abs/2007.00756.
13. Vogels, E.A. (2020). About one-in-five Americans use a smart watch or fitness tracker. https://www.pewresearch.org/fact-tank/2020/01/09/about-one-in-five-americans-use-a-smart-watch-or-fitness-tracker/.

CellPress
OPEN ACCESS

14. Hernandez, M., Milechin, L.E., Davis, S.K., DeLaura, R., Claypool, K.T., and Swiston, A. (2020). The Impact of Host-Based Early Warning on Disease Outbreaks. medRxiv. https://doi.org/10.1101/2020.03.06.20029793.

15. Bahloul, M.A., Chahid, A., and Laleg-Kirati, T.-M. (2020). Fractional-order SEIQRDP model for simulating the dynamics of COVID-19 epidemic. IEEE Open J. Eng. Med. Biol. *1*, 249–256.

16. Larremore, D.B., Wilder, B., Lester, E., Shehata, S., Burke, J.M., Hay, J.A., Tambe, M., Mina, M.J., and Parker, R. (2020). Test sensitivity is secondary to frequency and turnaround time for COVID-19 surveillance. medRxiv. https://doi.org/10.1101/2020.06.22.20136309.

17. Adans-Dester, C.P., Bamberg, S., Bertacchi, F.P., Caulfield, B., Chappie, K., Demarchi, D., Erb, M.K., Estrada, J., Fabara, E.E., Freni, M., et al. (2020). Can mHealth technology help mitigate the effects of the COVID-19 pandemic? IEEE Open J. Eng. Med. Biol. *1*, 243–248.

18. David Paltiel, A., Zheng, A., and Walensky, R.P. (2020). Assessment of SARS-CoV-2 screening strategies to permit the safe reopening of college campuses in the United States. JAMA Netw. Open *3*, e2016818.

19. Detect (2020). Home. https://www.detectstudy.org/.

20. SEA Lab (2020). TemPredict. https://www.sealab.ucsf.edu/tempredict.

21. Duke University (2020). CovIdentify. https://covidentify.covid19.duke.edu/.

22. Miller, D.J., Capodilupo, J.V., Lastella, M., Sargent, C., Roach, G.D., Lee, V.H., and Capodilupo, E.R. (2020). Analyzing changes in respiratory rate to predict the risk of COVID-19 infection. medRxiv. https://doi.org/10.1101/2020.06.18.20131417.

23. Mishra, T., Wang, M., Metwally, A.A., Bogu, G.K., Brooks, A.W., Bahmani, A., Alavi, A., Celli, A., Higgs, E., Dagan-Rosenfeld, O., et al. (2020). Pre-symptomatic detection of COVID-19 from smartwatch data. Nat. Biomed. Eng. *4*, 1208–1220.

24. Natarajan, A., Su, H.-W., and Heneghan, C. (2020). Assessment of physiological signs associated with COVID-19 measured using wearable devices. NPJ Digit. Med. *3*, 156.

25. Allen, W.E., Altae-Tran, H., Briggs, J., Jin, X., McGee, G., Shi, A., Raghavan, R., Kamariza, M., Nova, N., Pereta, A., et al. (2020). Population-scale longitudinal mapping of COVID-19 symptoms, behaviour and testing. Nat. Hum. Behav. *4*, 972–982.

26. The Economist. (2020). The southern hemisphere skipped flu season in 2020. https://www.economist.com/graphic-detail/2020/09/12/the-southern-hemisphere-skipped-flu-season-in-2020.

27. Centers for Disease Control and Prevention (2020). Estimated influenza illnesses, medical visits, hospitalizations, and deaths in the United States—2018–2019 influenza season. https://www.cdc.gov/flu/about/burden/2018-2019.html.

28. Centers for Disease Control and Prevention (2020). National Health and Nutrition Examination Survey Module 3: Weighting. https://wwwn.cdc.gov/nchs/nhanes/tutorials/module3.aspx.

29. Menni, C., Valdes, A.M., Freidin, M.B., Sudre, C.H., Nguyen, L.H., Drew, D.A., Ganesh, S., Varsavsky, T., Cardoso, M.J., El-Sayed Moustafa, J.S., et al. (2020). Real-time tracking of self-reported symptoms to predict potential COVID-19. Nat. Med. *26*, 1037–1040.

30. Centers for Disease Control and Prevention (2020). Influenza Positive Tests Reported to CDC by U.S. Clinical Laboratories 2019–2020 Season. https://www.cdc.gov/flu/weekly/weeklyarchives2019-2020/data/whoAllregt_cl17.html.

31. Centers for Disease Control and Prevention (2020). Frequently Asked Influenza (Flu) Questions: 2019–2020 Season. https://www.cdc.gov/flu/season/faq-flu-season-2019-2020.htm.

32. Developer (2020). Heart Rate. https://dev.fitbit.com/build/reference/web-api/heart-rate/.

33. Weerahandi, H., Hochman, K.A., Simon, E., Blaum, C., Chodosh, J., Duan, E., Garry, K., Kahan, T., Karmen-Tuohy, S., Karpel, H., et al. (2020). Post-discharge health status and symptoms in patients with severe COVID-19. medRxiv. https://doi.org/10.1101/2020.08.11.20172742.

34. Rimmer, A. (2020). Covid-19: impact of long term symptoms will be profound, warns BMA. BMJ *370*, m3218.

35. Quer, G., Radin, J.M., Gadaleta, M., Baca-Motes, K., Ariniello, L., Ramos, E., Kheterpal, V., Topol, E.J., and Steinhubl, S.R. (2020). Wearable sensor data and self-reported symptoms for COVID-19 detection. Nat. Med. https://doi.org/10.1038/s41591-020-1123-x.

36. Yancy, C.W. (2020). COVID-19 and African Americans. JAMA *323*, 1891–1892.

37. Office of Disease Prevention and Health Promotion (2020). Social Determinants of Health. https://www.healthypeople.gov/2020/topics-objectives/topic/social-determinants-of-health.

38. Stokes, E.K., Zambrano, L.D., Anderson, K.N., Marder, E.P., Raz, K.M., El Burad Felix, S., Tie, Y., and Fullerton, K.E. (2020). Coronavirus disease 2019 case surveillance—United States, January 22–May 30, 2020. MMWR Morb. Mortal. Wkly. Rep. *69*, 759–765.

39. Killerby, M.E., Link-Gelles, R., Haight, S.C., Schrodt, C.A., England, L., Gomes, D.J., Shamout, M., Pettrone, K., O'Laughlin, K., Kimball, A., et al. (2020). Characteristics associated with hospitalization among patients with COVID-19—Metropolitan Atlanta, Georgia, March–April 2020. MMWR Morb. Mortal. Wkly. Rep. *69*, 790–794.

40. Gold, J.A.W., Wong, K.K., Szablewski, C.M., Patel, P.R., Rossow, J., da Silva, J., Natarajan, P., Morris, S.B., Fanfair, R.N., Rogers-Brown, J., et al. (2020). Characteristics and clinical outcomes of adult patients hospitalized with COVID-19—Georgia, March 2020. MMWR Morb. Mortal. Wkly. Rep. *69*, 545–550.

41. Price-Haywood, E.G., Burton, J., Fort, D., and Seoane, L. (2020). Hospitalization and mortality among black patients and white patients with Covid-19. N. Engl. J. Med. *382*, 2534–2543.

42. Millett, G.A., Jones, A.T., Benkeser, D., Baral, S., Mercer, L., Beyrer, C., Honermann, B., Lankiewicz, E., Mena, L., Crowley, J.S., et al. (2020). Assessing differential impacts of COVID-19 on black communities. Ann. Epidemiol. *47*, 37–44.

43. Shah, N.H., Callahan, A., Fries, J.A., Gombar, S., and Patel, B. (2020). Profiling presenting symptoms of patients screened for SARS-CoV-2. Medium https://medium.com/@nigam/an-ehr-derived-summary-of-the-presenting-symptoms-of-patients-screened-for-sars-cov-2-910ceb1b22b9.

44. Rentsch, C.T., Kidwai-Khan, F., Tate, J.P., Park, L.S., King, J.T., Jr., Skanderson, M., Hauser, R.G., Schultze, A., Jarvis, C.I., Holodniy, M., et al. (2020). Covid-19 by Race and Ethnicity: A National Cohort Study of 6 Million United States Veterans. medRxiv. https://doi.org/10.1101/2020.05.12.20099135.

45. Centers for Disease Control and Prevention (2020). Health Equity Considerations and Racial and Ethnic Minority Groups. https://www.cdc.gov/coronavirus/2019-ncov/need-extra-precautions/racial-ethnic-minorities.html.

46. Centers for Disease Control and Prevention (2020). Laboratory-Confirmed COVID-19-Associated Hospitalizations. https://gis.cdc.gov/grasp/covidnet/COVID19_3.html.

47. Johns Hopkins University & Medicine (2020). Cumulative Cases. https://coronavirus.jhu.edu/data/cumulative-cases.

48. Centers for Disease Control and Prevention (2020). 2019–2020 U.S. Flu Season: Preliminary In-Season Burden Estimates. https://www.cdc.gov/flu/about/burden/preliminary-in-season-estimates.htm.

49. Patel, M.S., Foschini, L., Kurtzman, G.W., Zhu, J., Wang, W., Rareshide, C.A.L., and Zbikowski, S.M. (2017). Using wearable devices and smartphones to track physical activity: initial activation, sustained use, and step counts across sociodemographic characteristics in a national sample. Ann. Intern. Med. *167*, 755–757.

50. Oran, D.P., and Topol, E.J. (2020). Prevalence of asymptomatic SARS-CoV-2 infection: a narrative review. Ann. Intern. Med. *173*, 362–367.

51. Federal Laboratory Consortium for Technology Transfer (2020). BARDA, Evidation Health partner on COVID-19 self-monitoring study. https://

federallabs.org/news/barda-evidation-health-partner-on-covid-19-self-monitoring-study.

52. Flannery, B., Meece, J.K., Williams, J.V., Martin, E.T., Gaglani, M., Jackson, M.L., and Talbot, H.K. (2020). Systematic testing for influenza and COVID-19 among patients with respiratory illness. Clin. Infect. Dis. https://doi.org/10.1093/cid/ciaa1023.
53. Williams, S.N., Armitage, C.J., Tampe, T., and Dienes, K. (2020). Public attitudes towards COVID-19 contact tracing apps: a UK-based focus group study. medRxiv. https://doi.org/10.1101/2020.05.14.20102269.
54. Goldsack, J.C., Izmailova, E.S., Menetski, J.P., Hoffmann, S.C., Groenen, P.M.A., and Wagner, J.A. (2020). Remote digital monitoring in clinical trials in the time of COVID-19. Nat. Rev. Drug Discov. *19*, 378–379.
55. Mahmood, S., Hasan, K., Colder Carras, M., and Labrique, A. (2020). Global preparedness against COVID-19: we must leverage the power of digital health. JMIR Public Health Surveill. *6*, e18980.
56. COVID-19 Research Database (2020). COVID-19 research database. https://covid19researchdatabase.org/.
57. SAGE Bionetworks (2020). Synapse. https://www.synapse.org/.
58. Achievement (2020). Achievement. https://www.myachievement.com/.
59. Deering, S., Grade, M.M., Uppal, J.K., Foschini, L., Juusola, J.L., Amdur, A.M., and Stepnowsky, C.J. (2019). Accelerating research with technology: rapid recruitment for a large-scale web-based sleep study. JMIR Res. Protoc. *8*, e10974.
60. Kumar, S., Tran, J.L., Lee, W., Bradshaw, B., Foschini, L., and Juusola, J. (2018). Longitudinal data from activity trackers show that those with greater inconsistency in activity levels are more likely to develop more severe depression. Value Health *21*, S191.
61. Backer, J.A., Klinkenberg, D., and Wallinga, J. (2020). Incubation period of 2019 novel coronavirus (2019-nCoV) infections among travellers from Wuhan, China, 20–28 January 2020. Eurosurveillance *25*, https://doi.org/10.2807/1560-7917.ES.2020.25.5.2000062.
62. McAloon, C., Collins, Á., Hunt, K., Barber, A., Byrne, A.W., Butler, F., Casey, M., Griffin, J., Lane, E., McEvoy, D., et al. (2020). Incubation period of COVID-19: a rapid systematic review and meta-analysis of observational research. BMJ Open *10*, e039652.
63. Tan, W.Y.T., Wong, L.Y., Leo, Y.S., and Toh, M.P.H.S. (2020). Does incubation period of COVID-19 vary with age? A study of epidemiologically linked cases in Singapore. Epidemiol. Infect. *148*, https://doi.org/10.1017/S0950268820001995.
64. Wang, Y., Wang, Y., Chen, Y., and Qin, Q. (2020). Unique epidemiological and clinical features of the emerging 2019 novel coronavirus pneumonia (COVID-19) implicate special control measures. J. Med. Virol. *92*, 568–576.
65. Tudor-Locke, C., Johnson, W.D., and Katzmarzyk, P.T. (2011). U.S. population profile of time-stamped accelerometer outputs: impact of wear time. J. Phys. Act. Health *8*, 693–698.
66. Wood, S.N. (2011). Fast stable restricted maximum likelihood and marginal likelihood estimation of semiparametric generalized linear models. J. R. Stat. Soc. Ser. B Stat. Methodol. *73*, 3–36.

PATTER, Volume *2*

Supplemental Information

Characterizing COVID-19 and Influenza Illnesses in the Real World via Person-Generated Health Data

Allison Shapiro, Nicole Marinsek, Ieuan Clay, Benjamin Bradshaw, Ernesto Ramirez, Jae Min, Andrew Trister, Yuedong Wang, Tim Althoff, and Luca Foschini

Supplementary Note 1: Institutional Review Board

This study received expedited review and IRB approval from Solutions IRB (Protocol ID #2018/11/8). Waiver of informed consent was granted by the IRB. Prior to each questionnaire, participants were notified about how their survey responses and behavioral data will be used for research purposes through a disclosure.

Supplementary Note 2: Questionnaire

Weekly 1-Click Item

1. Have you experienced flu-like symptoms in the past 7 days (such as fever, chills, cough, shortness of breath, and/or headache)? If you had flu-like symptoms in the past 7 days, but have recovered, please still answer YES.

    (a) Yes [Symptom Experience Survey]

    (b) No [Infection Risk Factors Survey]

Symptom Experience Survey

1. What is your current zip code? (Where you live and spend the majority of your time) *If you are currently staying in a different location for an extended period of time, please enter your current zip code*.

    (a) numeric 5-digit entry

2. When did you first begin experiencing flu-like symptoms? If you don't recall the exact date, please provide the best estimate.

    (a) calendar date selection

3. As of today, do you feel that you have completely recovered from your illness?

    (a) Yes

    (b) No

[IF Q3 = A, THEN Q4]
[IF Q3 = B, THEN THEN SKIP TO Q5]

4. When did you feel you were completely recovered from your illness? If you don't recall the exact date, please provide the best estimate.

    (a) calendar date selection

5. We'd like to know more about the symptoms you experienced. Looking back over the past 7 days, did you have any of the following symptoms? Please select all that apply.

    (a) Cough

    (b) Body/Muscle Ache

    (c) Fever or feeling feverish

    (d) Chills or shivering

(e) Sweats
(f) Headache
(g) Sore throat or itchy/scratchy throat
(h) Feeling more tired than usual
(i) Nasal congestion or runny nose
(j) Sneezing
(k) I did not experience any flu-like symptoms
(l) Other

[IF Q4 = K, SURVEY END]

6. We'd like to know more about the symptoms you experienced. Looking back over the past 7 days, please indicate on which days you felt the following symptoms.

    (a) Matrix carry forward symptoms; check all that apply: Today, Yesterday, 2 days ago, 3 days ago, 4 days ago, 5 days ago, 6 days ago

7. Looking back over the past 7 days, did you have any of the additional symptoms below

    (a) Shortness of breath and/or difficulty breathing
    (b) Persistent pain or pressure in the chest
    (c) Loss of sense of smell
    (d) None of the above

[IF Q7 = A-C, THEN Q8]
[IF Q7 = D, THEN THEN SKIP TO Q9]

8. Looking back over the past 7 days, please indicate on which days you felt the following additional symptoms.

    (a) Matrix carry forward other symptoms; check all that apply: Today, Yesterday, 2 days ago, 3 days ago, 4 days ago, 5 days ago, 6 days ago

9. Thinking about your flu-like symptoms over the last 7 days, on what day did you feel the worst?

    (a) Today
    (b) Yesterday
    (c) 2 days ago
    (d) 3 days ago
    (e) 4 days ago
    (f) 5 days ago
    (g) 6 days ago

10. Did you seek medical attention from a healthcare provider at a clinic or urgent care facility for this flu or flu-like illness?

    (a) Yes
    (b) No

[IF Q10 = A, THEN Q11]
[IF Q10 = B, THEN SKIP TO Q23]

11. Where did you seek care from a healthcare provider?

    (a) Primary care clinic (e.g. family medicine, internal medicine)
    (b) Urgent care facility
    (c) Emergency room (ER)
    (d) Ear, nose, and throat (otolaryngology) clinic
    (e) Infectious disease clinic
    (f) Other

12. Did the healthcare provider diagnose you as having the flu?

    (a) Yes
    (b) No
    (c) I don’t know / I can’t remember

13. Did the healthcare provider perform any of the following tests? Select all that apply.

    (a) Nasal swab
    (b) Throat swab
    (c) Symptoms only (no lab test)
    (d) I don’t know / I can’t remember
    (e) Other (please specify)

14. Did the healthcare provider diagnose you as having coronavirus disease (also known as COVID-19)?

    (a) Yes
    (b) No
    (c) I am waiting for my diagnosis
    (d) I don’t know / I can’t remember

15. Did you take any of the following tests for your coronavirus diagnosis? Select all that apply.

    (a) Nasal swab
    (b) Throat swab (c) Blood test
    (d) Spit test / kit
    (e) Symptoms only (no lab test)
    (f) I don’t know / I can’t remember
    (g) Other (please specify)

16. Where did you take the COVID-19 diagnostic test?

    (a) In a clinic or hospital
    (b) At a drive through testing facility
    (c) At home testing kit
    (d) Other (please specify)
    (e) None of the above

17. Were you hospitalized as a consequence of this flu or flu-like illness? *Hospitalization is when you leave the emergency room (ER) and are admitted to the inpatient hospital based on a doctor's order. Even if you stayed overnight in the ER, this is not considered a hospitalization*.

    (a) Yes (b) No

18. Were you told to self-quarantine (stay in your home without leaving for any reason) by a medical professional?

    (a) Yes

    (b) No

    (c) I don't know / I can't remember

19. Did a healthcare provider prescribe any medications to treat or manage your current symptoms?

    (a) Yes

    (b) No

    (c) I don't know / I can't remember

[IF Q19 = A, THEN Q20]

[IF Q19 = B, THEN SKIP TO Q23]

20. Which of the following medications were you prescribed to treat or manage your symptoms? Select all that apply.

    (a) Xofluza (baloxavir marboxil)

    (b) Tamiflu (oseltamivir)

    (c) Relenza (zanamivir)

    (d) Antibiotics (Z-pak, amoxicillin, Augmentin, doxycycline) (e) Other

21. When did you take your first dose of [CARRY FORWARD MEDICATION NAME]? Please enter the date in MM/DD/YYYY format.

    (a) Date entry

22. Did you ever miss any doses or decide not to take [CARRY FORWARD MEDICATION NAME]? a.

    (a) I missed at least one dose of this medication

    (b) I did not take any doses of this medication

    (c) I did not miss any doses of medication

    (d) I don't know / I can't remember

23. Did you take any over-the-counter (non-prescription) medications to treat or manage your current symptoms in the past 7 days?

    (a) Yes

    (b) No

    (c) I don't know / I can't remember

[IF Q23 = A, THEN Q24]

[IF Q23 = B, THEN SKIP TO Q25]

24. Which of the following over-the-counter (non-prescription) medications did you personally decide to take to treat or manage your current symptoms in the past 24 hours? Select all that apply.

(a) Fever reducers or pain relievers (ibuprofen, aspirin, Advil, Tylenol, Aleve, acetaminophen)

(b) Cough suppressants (Delsym, Robitussin, dextromethorphan)

(c) Chest or mucus decongestants (Mucinex, guaifenesin)

(d) Nasal decongestants (Sudafed, Sudafed PE, Afrin, Flonase, phenylephrine, pseudoephedrine, fluticasone propionate)

(e) I don't know / can't remember

(f) Other

25. How many people (other than yourself) live in your household?

(a) 0

(b) 1

(c) 2

(d) 3

(e) 4 (f) 5

(g) 6

(h) 7

(i) 8

(j) 9

(k) 10

(l) >10

26. Have any members of your household (other than yourself) experienced flu-like illness this flu season?

(a) Yes

(b) No

(c) I live alone

[IF Q26 = A, THEN Q27]

[IF Q26 = B or C, THEN SKIP TO Q29]

27. How many members of your household, by age group listed below, have experienced flu-like symptoms during this flu season (September 2019 to today)? If no household member in your household experienced symptoms within an age group please enter 0. [numeric entry]

(a) Number of household members 0-4 years old experiencing flu-like symptoms

(b) Number of household members 5-17 years old experiencing flu-like symptoms

(c) Number of household members 18-49 years old experiencing flu-like symptoms

(d) Number of household members 50-64 years old experiencing flu-like symptoms

(e) Number of household members 65+ years old experiencing flu-like symptoms

28. Have any members of your household been diagnosed with coronavirus disease (also known as COVID-19?

(a) Yes

(b) No

29. Have you been in close contact with anyone outside your household (e.g., family members, friends, coworkers, acquaintances) who has experienced flu-like symptoms recently? *Close contact can include*

direct physical contact, face-to-face contact for longer than 15 minutes, exchange of bodily fluids, or being within 6 feet of the person for more than 15 minutes.

(a) Yes, within the last 7 days
(b) Yes, within the last 14 days
(c) Yes, over 14 days ago
(d) No
(e) I don't know / I'm not sure

30. Have you recently been in contact with someone who was diagnosed with coronavirus? *Close contact can include direct physical contact, face-to-face contact for longer than 15 minutes, exchange of bodily fluids, or being within 6 feet of the person for more than 15 minutes*.

    (a) Yes, within the last 7 days
    (b) Yes, within the last 14 days
    (c) Yes, over 14 days ago
    (d) No
    (e) I don't know / I'm not sure

31. Did you miss school or work due to your illness?

    (a) No, I did not miss any school or work during my illness
    (b) I missed 1 day of school or work
    (c) I missed 2 days of school or work
    (d) I missed 3 days of school or work
    (e) I missed more than 3 days of school or work
    (f) Illness occurred on a weekend or other day(s) off
    (g) I am retired and/or school or work days don't apply to me
    (h) I don't know / I don't remember

32. Looking back over the past 7 days, which days have you practiced social distancing or isolation behaviors (e.g., working remotely, limited the time spent in crowds, increasing the amount of time spent at home)? Please select all that apply.

    (a) Today
    (b) Yesterday
    (c) Two days ago
    (d) Three days ago
    (e) Four days ago
    (f) Five days ago
    (g) Six days ago
    (h) I did not practice social distancing in the last 7 days

33. Did you receive the flu vaccine (sometimes called the flu shot) this flu season (September 2019 to today)?

    (a) Yes
    (b) No

(c) I don't know / I can't remember

34. Did you receive the flu vaccine last flu season? (September 2018 - March 2019)

   (a) Yes
   (b) No
   (c) I don't know / I can't remember

35. Please select the statement below that describes whether you typically get a flu shot (or another form of flu vaccine).

   (a) I never have gotten a flu shot
   (b) I rarely get a flu shot
   (c) I get a flu shot every year
   (d) I sometimes get a flu shot

36. Have you recently traveled on an airplane?

   (a) Yes, within the last 7 days
   (b) Yes, within the last 14 days
   (c) Yes, over 14 days ago
   (d) No

37. Have you recently participated in any large public gatherings of over 250 people (e.g., concerts, sporting events, amusement parks)?

   (a) Yes, within the last 7 days
   (b) Yes, within the last 14 days
   (c) Yes, over 14 days ago
   (d) No

38. Are you or one of your household members a healthcare worker (i.e., doctor, dentist, nurse, nurse's aid, paramedic, physician's assistant, home healthcare aid, hospital worker, pharmacist, or other type of healthcare worker)? Please select all that apply.

   (a) I am, and I am currently working
   (b) I am, but I am NOT currently working
   (c) One of my household members is, and they are currently working
   (d) One of my household members is, but they are NOT currently working
   (e) No one in my household is a healthcare worker

39. Were you diagnosed by a healthcare provider with any of the following health problems either during your flu illness or since you recovered from your flu illness? Please select all that apply. [CHECKBOX]

   a. Chest infection (pneumonia, acute lung injury)
   b. Worsening of asthma or COPD
   c. Bloodstream infection (sepsis)
   d. Ear infection (otitis media)
   e. Sinus infection (sinusitis)
   f. Brain inflammation (encephalitis or encephalopathy)

g. Stroke
h. Inflammation of the heart or the covering of the heart (myocarditis and/or pericarditis)
i. Worsening of heart failure
j. Heart attack (myocardial infarction)
k. Muscle inflammation (myositis or rhabdomyolysis)
l. No, I was not diagnosed with any of these conditions

[IF Q38 = YES TO ANY → GO TO Q39 for each complication selected
IF Q38 = "l" —> end survey]]

40. Approximate date that the [CARRY FORWARD COMPLICATION SELECTIONS] first occurred. Please enter the date in MM/DD/YYYY format.
    a. Date text entry

Supplementary Note 3: Methods

Survey Filtering.

Survey responses with self-reported illness onset dates or recovery dates that occurred 30 or more days before the survey completion date were excluded, leaving 158,999 survey responses from 73,728 unique participants. Survey responses with invalid illness onset and/or recovery dates (defined as dates occurring after the survey date or responses in which the illness recovery date occurred before the illness onset date) were also removed, leaving 149,309 survey responses from 71,556 unique individuals. Finally, the set of survey responses was restricted to one survey per participant per day. If one participant attempted more than one survey in a given day, the less complete survey was excluded.

Inference of Distinct ILI Events.

Participants could submit new survey responses as frequently as once per week, with no maximum limit. Therefore, individual symptom trajectories for an ILI event had to be inferred by concatenating and reconciling multiple surveys responses, for example, if the participant were midway through their illness when they submitted their first survey their next survey could describe the second half of their illness.

We inferred ILI events by merging multiple surveys from the same participant with date ranges encompassing symptoms onset and recovery that overlapped or were separated by no more than 2 days. Participants with more than 5 ILI events were removed, eliminating 16,878 surveys and 1,639 participants, and leaving 126,014 survey responses, corresponding to 99,604 distinct ILI events and 69,034 participants.

This gives the set of discrete ILI events per participant, from which we will select only 1 for analysis. If a participant has a diagnosed COVID-19 ILI event, that event is selected, otherwise the most recent ILI event is selected. This process removes a further 40,357 surveys and 30,567 distinct ILI events, and 0 participants. Participants reporting multiple non-overlapping

diagnosed COVID-19 events were then excluded (excluding 7 survey responses, 6 distinct ILI events, and 3 participants).

Reconciliation of Merged Survey Responses.

At this point, we have one ILI event per participant, corresponding to 85,650 surveys for 69,031 distinct ILI events across 69,031 participants. We then reconcile responses to derive a single value per item. For example, the date of onset and recovery are taken as the earliest and latest reported date for that ILI event, respectively.
Participants who reported being diagnosed with both flu and COVID-19 (N=83) were assigned to the COVID-19 cohort, under the rationale that some individuals may consider COVID-19 to be a type of flu, and the relative order in the questionnaire (flu preceding COVID-19). Among the 41 COVID-19 cases with dense Fitbit data, 14 also reported having the flu (12 with dense HR data, 12 with dense sleep data, 11 with dense steps data.
Flu events drawing from multiple surveys responses may have differing symptoms reports for the same calendar date. Such day-level values (e.g., symptoms reported for a specific day) were collapsed if identical, and if not, the survey submitted on the date closest to the calendar date was used. Participants were also allowed to report annotations, for example "the worst day", during a given event. These are highly subjective, thus all responses were retained, with a given date coded as "one of the worst days" if the participant indicated as such in any survey. For event-level categorical features, the algorithm described in Figure S1 was used to collapse surveys to a single response. Numerical event-level features, for example the number of household members who have experienced ILI symptoms, were aggregated by taking the maximum value reported. All other features which could not be reconciled were simply aggregated as concatenated unique values.

In our selection of ILI events,we select COVID-19 events or the most recent ILI event for each participant. This biases our analysis towards later calendar dates when sensor data is most affected by social distancing. For this reason, we have included a chronologically parallel group of Non-COVID-19 Flu patients. A second issue is that we could be missing participants’ most severe ILI events, which could have happened earlier in the season. We will continue to monitor symptomatic and behavioral changes associated with COVID-19 and non-COVID-19 ILIs as more events are captured and as guidance on social distancing and stay-at-home measures are relaxed. Further analysis will focus on how strongly these measures confound our observations.

Statistical Testing.

A two-step statistical testing procedure was used to test for differences in demographics, healthcare care-seeking behavior, medical outcomes, and symptoms among the three cohorts. First, for each sub-analysis (i.e., demographics, medical care-seeking, and symptom prevalence), a series of chi-squared tests of independence were performed to test for an association between the three cohorts and the different possible outcomes for each category. A Bonferroni correction was applied to adjust for running multiple chi-squared tests in each sub-analysis. Second, follow-up two-proportion z-tests were performed to test for differences in proportions for each outcome and each pair of cohorts. These follow-up tests were only

performed for the categories with significant cohort differences as determined by the chi-squared tests.

Wearable Sensor Data Preparation.

The pipeline for preparing the surveys for analysis is described in detail in Supplementary Note 3 and summarized here.

Of the 6,926 participants with diagnosed ILI events, 4,778 (69%) have shared at least one wearable device connected to the Achievement platform: 2,582 (37%) participants had connected Apple Watches, 2,166 (31%) had connected Fitbit devices, 420 (6%) had connected Garmin devices, 123 (2%) had connected Withings devices, and 17 (0·2%) had connected Misfit devices. We focus the analysis of sensor data on the subset of participants with connected Fitbit devices, consisting of minute-by-minute steps, heart rate recordings, and sleep states, available for a subset of study participants. This data was collected from 2019-11-01 through 2020-05-13 and analyzed to investigate the impact of COVID-19 and flu on everyday behavior and physiology.

Since the sensor data was collected passively in real-world settings, daily sensor wear-time varied across participants and study days. We implemented a three step procedure to enforce adequate data density around each ILI event prior to analysis. First, we estimated if the sensor was worn for each participant for each minute in the study period. Periods of non-wear-time were defined as 180 or more consecutive minutes of zero steps or null heart rate recordings. Second, days with 10 or more hours of sensor wear-time were tagged as valid for analysis. For the sleep data, days with at least one main sleep period recorded by Fitbit were considered valid. Third, the analysis set was restricted to only include participants with 1) at least 10% of valid days for each day of the week in the baseline period (defined as all participant-days that occurred outside the window of 10 days prior to and 20 days after illness onset) and 2) at least 50% valid days in the time period surrounding the ILI event (defined as all days within the window of 10 days prior to 20 days after illness onset). Dense sensor data was available for 41 COVID-19 patients (36 with steps, 33 with RHR, and 35 with sleep), 85 Non-COVID-19 Flu patients (80 with steps, 60 with RHR, and 64 with sleep), and 1226 Pre-COVID-19 Flu patients (1193 with steps, 1025 with RHR, and 979 with sleep). Sensitivity analysis on the valid day thresholds was conducted and results did not change significantly when removing the requirement of having 10% of valid days for each day of week or lowering the percentage of individual valid days to as low as 30%. The pipeline for preparing the wearable data for analysis is illustrated in Supplementary Figure S1 (b).

Elevated RHR Prevalence.

Similarly to previous work,[1] we examined the fraction of each cohort with elevated RHR in the days preceding and following ILI onset. First, days without RHR recordings were imputed in order to ensure that the cohorts were the same across days of interest. Imputed RHR values were generated from predictions of a mixed effects regression model that was fit to all participant-days that RHR was recorded. The model specified fixed effects for the week of the year to control for time of year effects (more specifically, this consisted of three terms for the

1st, 2nd, and 3rd expansions of an ordinal variable for week of flu season), a categorical fixed effect for the day of the week to account for differences in activity patterns by day of week, a fixed effect for the average activity level in the participants' state of residence to control for different state-wide shelter-in-place and social distancing measures, and a random intercept for each participant's baseline activity level to control for individual differences in activity levels. The model was fit to all participant-days with a RHR recording using the lme4 package for R.[2] Note that the imputed values were used only to fill days when RHR was not recorded, the observed value was used on all other days .

Next, in order to account for individual differences in RHR when defining thresholds for elevated RHR, RHR values were converted to z-scores using each participant's RHR mean and standard deviation across all days. The fraction of each cohort with elevated RHR was computed for the days surrounding the ILI event, defined as 10 days prior to 20 days after ILI onset. Elevated RHR was defined as being greater than 1 standard deviation above the participant's mean RHR. Two-proportion z-tests were performed to answer the following two questions: 1. Does a greater fraction of the COVID-19 cohort have elevated RHR in the days surrounding ILI onset compared to days prior to ILI onset and 2. Does the fraction of participants with elevated RHR surrounding ILI onset differ between COVID-19 and Flu cohorts? The time window surrounding ILI onset was defined as starting two days prior to self-reported illness onset and ending two days afterward (Days -2 to 2). We conservatively allowed 2 days for physiological changes before any symptom was reported, and 2 days after the onset of symptoms as the time horizon within which actions could be taken that would not otherwise be taken without information from the wearable device. For the purpose of the first statistical test, the time period prior to ILI onset was defined as Days -10 to -5 relative to self-reported illness onset. This time window was selected because it fell within the time period that data density was enforced, it was the same duration as the time window surrounding ILI onset, and the median time from exposure to COVID-19 to the development of symptoms is 5 days[3].

Behavioral and Physiological Changes During ILI Events.

In order to characterize daily changes associated with COVID-19 and flu events, we measured deviations from typical healthy measurements (RHR, step count, sleep hours) that occurred while participants were ill. We used a model on symptom-free days (conservatively assumed all days excluding the 10 days before symptoms onset and within 20 days after symptoms onset) to generate individualized estimates of daily measurements that would have been recorded in the counterfactual scenario that the participant did not fall ill, and then computed the excess, defined as the difference (observed - estimated), on the days surrounding symptoms onset, and finally report the excess as a measure of deviations from expected typical measurements. The symptom-free day model was a mixed effects regression model with the same specification as what was used to impute missing RHR values in the previous analysis. The key difference in this analysis was that, in order to generate estimates based only on assumed symptom-free days, we excluded all data within 10 days before symptoms onset and within 20 days after symptoms onset when fitting the model. In order to visualize the time course of behavioral changes during COVID-19 and flu events, we fit generalized additive mixed models with spline

smoothing functions and random intercepts to the daily excess time series for each cohort using the mgcv package for R.[4] This procedure was performed three separate times, for each of the channels considered: daily total step counts, daily RHR, and total daily sleep minutes.

Supplementary Note 4: Comorbidity Prevalence

Table S1 describes self-reported comorbidities observed in our ILI cohorts.

	COVID-19	Non-COVID-19 Flu	Pre-COVID-19 Flu
Anxiety	65 (28.3%)	122 (28.6%)	1915 (30.5%)
Depression	62 (27.0%)	104 (24.4%)	1868 (29.8%)
Asthma	56 (24.3%)	79 (18.5%)	1247 (19.9%)
Migraines	50 (21.7%)	75 (17.6%)	1225 (19.5%)
Chronic Pain	30 (13.0%)	40 (9.4%)	572 (9.1%)
Hypertension	17 (7.4%)	46 (10.8%)	718 (11.5%)
PCOS	16 (7.0%)	16 (3.8%)	315 (5.0%)
GERD	15 (6.5%)	46 (10.8%)	599 (9.6%)
Mental Health (Excluding Depression/Anxiety)	15 (6.5%)	32 (7.5%)	499 (8.0%)
Insomnia	15 (6.5%)	37 (8.7%)	541 (8.6%)
Sleep Apnea	11 (4.8%)	20 (4.7%)	335 (5.3%)
Restless Leg Syndrome	10 (4.3%)	12 (2.8%)	231 (3.7%)
Type 2 Diabetes	10 (4.3%)	10 (2.3%)	254 (4.1%)
Hypo- or Hyperthyrodism	9 (3.9%)	32 (7.5%)	418 (6.7%)
Fibromyalgia	9 (3.9%)	16 (3.8%)	215 (3.4%)
High Cholesterol	9 (3.9%)	21 (4.9%)	352 (5.6%)
Gestational Diabetes	8 (3.5%)	13 (3.1%)	187 (3.0%)
Cancer	6 (2.6%)	13 (3.1%)	165 (2.6%)

Arrhythmia	6 (2.6%)	5 (1.2%)	165 (2.6%)
Psoriasis	5 (2.2%)	14 (3.3%)	147 (2.3%)
Type 1 Diabetes	4 (1.7%)	6 (1.4%)	63 (1.0%)
Rheumatoid Arthritis	4 (1.7%)	7 (1.6%)	135 (2.2%)
Stroke	3 (1.3%)	3 (0.7%)	31 (0.5%)
Heart Attack	2 (0.9%)	3 (0.7%)	28 (0.4%)
IBS or IBD	2 (0.9%)	9 (2.1%)	94 (1.5%)
COPD	2 (0.9%)	4 (0.9%)	61 (1.0%)
Seasonal Allergies	2 (0.9%)	5 (1.2%)	109 (1.7%)
Lupus	1 (0.4%)	0 (0.0%)	7 (0.1%)
Coronary Heart Disease	1 (0.4%)	0 (0.0%)	13 (0.2%)
Multiple Sclerosis	1 (0.4%)	4 (0.9%)	30 (0.5%)
Alzheimer's Disease	1 (0.4%)	1 (0.2%)	3 (0.0%)
Heart Failure	0 (0.0%)	5 (1.2%)	27 (0.4%)
Neurodegenerative	0 (0.0%)	1 (0.2%)	4 (0.1%)
Arthritis	0 (0.0%)	3 (0.7%)	37 (0.6%)
Osteoporosis	0 (0.0%)	6 (1.4%)	51 (0.8%)

Table S1. Prevalence of self-reported co-morbidities for the COVID-19 (N=230), Non-COVID-19 Flu (N=426), and Pre-COVID-19 Flu (N=6270) cohorts.

Supplementary Note 5: Symptom Labels

Table S2 describes the labels and associated descriptions used in our surveys for this work.

Symptom Label	Symptom Description in Survey
Cough	Cough
Headache	Headache

Body Muscle Ache	Body/Muscle Ache
Fatigue	Feeling more tired than usual
Fever	Fever or feeling feverish
Chills or Shivering	Chills or shivering
Sore Throat	Sore throat or itchy/scratchy throat
Nasal Congestion	Nasal congestion or runny nose
Sweats	Sweats
Sneezing	Sneezing
Chest Pain/Pressure	Persistent pain or pressure in the chest
Shortness of Breath	Shortness of breath and/or difficulty breathing
Anosmia	Loss of sense of smell

Table S2. Full symptom descriptions included in the survey for each abbreviated symptom label. The Chest Pain/Pressure, Shortness of Breath, and Anosmia symptoms were only included in the updated survey.

Supplementary Note 6: Symptom Reporting

Figure S1 describes the percentage of each ILI cohort reporting daily symptoms between one week prior and 4 weeks post symptom onset. Figure S2 describes the percentage of observed symptom reporting for hospitalized and non-hospitalized COVID-19 cohorts, between one week prior and 4 weeks post symptom onset.

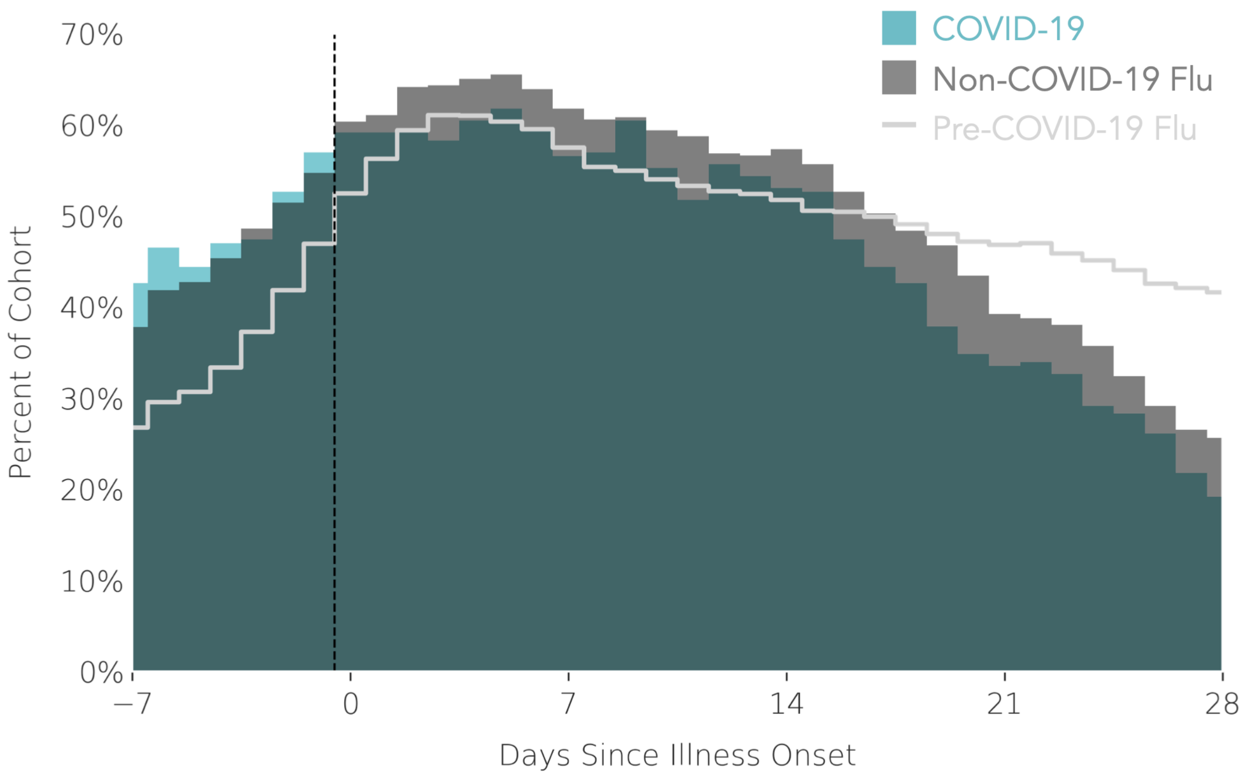

Fig. S1. Percentage of COVID-19 (N=230; blue), Non-Covid Flu (N=426; gray), and Pre-Covid Flu (N=6270, light gray trace) cohorts with symptom reports for days -7 to 28 since illness onset.

We note that our approach may underestimate disease severity, due to participants not reporting symptoms, not wearing sensors in days when symptoms are most severe, or during hospitalization events (see Figure S3).

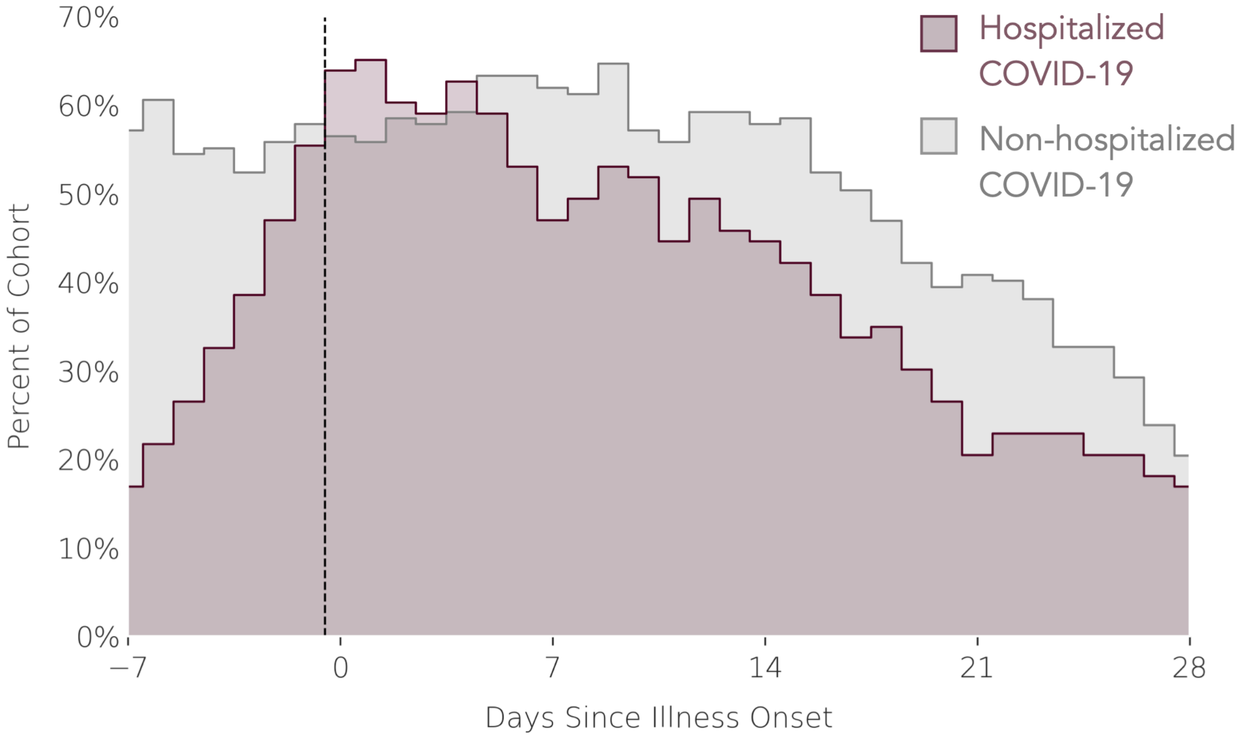

Fig. S2. Percentage of the Hospitalized (N=83, purple) and Non-hospitalized (N=147, gray) COVID-19 sub-cohorts with symptom reports for days -7 to 28 since illness onset.

Supplementary Note 7: Sensor Data Coverage

A summary of coverage of wearable sensor data over the course of the study is visualized in Figure S3.

We recognize that our analyses do not immediately translate to real-time implementation of COVID-19 monitoring, due to lag in data collection that comes from sensor and data synchronization. Lags in our dataflow are nevertheless small compared to the gains in symptom detection and reporting compared to canonical practice.

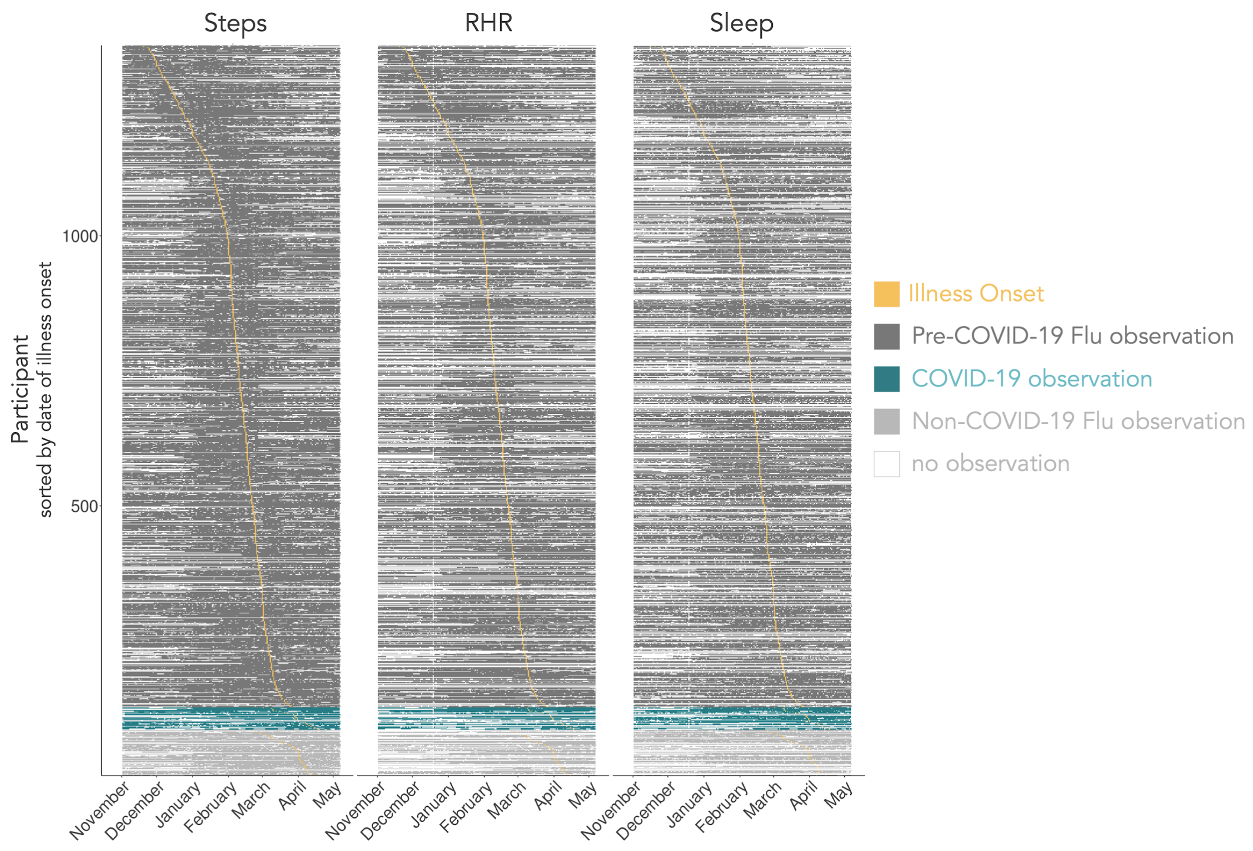

Fig. S3. Coverage of Fitbit steps, sleep, and RHR data on each calendar date of the study, color-coded by cohort. Each row is one participant (ordered by date of ILI-onset) and each column is one calendar date. Shaded days indicate that wearable data was recorded on that day from that participant. Days highlighted in yellow indicate the ILI onset dates.

Supplementary Note 8: Data Availability

De-identified study data will be made available to qualified researchers on the Sage Synapse platform[5] in September 2020.

Supplementary references

1. Radin, J.M., Wineinger, N.E., Topol, E.J., and Steinhubl, S.R. (2020). Harnessing wearable device data to improve state-level real-time surveillance of influenza-like illness in the USA: a population-based study. The Lancet Digital Health 2, e85–e93. Available at: https://www.sciencedirect.com/science/article/pii/S2589750019302225/pdfft?md5=785c228a82

10aa6e9a68aaa482f2274d&pid=1-s2.0-S2589750019302225-main.pdf [Accessed June 2, 2020].

2. Bates, D., Mächler, M., Bolker, B., and Walker, S. (2015). Fitting Linear Mixed-Effects Models Using lme4. J. Stat. Softw. 67. Available at: http://www.jstatsoft.org/v67/i01/.

3. Lauer, S.A., Grantz, K.H., Bi, Q., Jones, F.K., Zheng, Q., Meredith, H.R., Azman, A.S., Reich, N.G., and Lessler, J. (2020). The Incubation Period of Coronavirus Disease 2019 (COVID-19) From Publicly Reported Confirmed Cases: Estimation and Application. Ann. Intern. Med. 172, 577–582. Available at: http://dx.doi.org/10.7326/M20-0504.

4. Wood, S.N. (2011). Fast stable restricted maximum likelihood and marginal likelihood estimation of semiparametric generalized linear models. Journal of the Royal Statistical Society: Series B (Statistical Methodology) 73, 3–36. Available at: https://rss.onlinelibrary.wiley.com/doi/abs/10.1111/j.1467-9868.2010.00749.x [Accessed June 2, 2020].
